# HapFABIA: Identification of very short segments of identity by descent characterized by rare variants in large sequencing data

**DOI:** 10.1093/nar/gkt1013

**Published:** 2013-10-29

**Authors:** Sepp Hochreiter

**Affiliations:** Institute of Bioinformatics, Johannes Kepler University, Linz, Austria

## Abstract

Identity by descent (IBD) can be reliably detected for long shared DNA segments, which are found in related individuals. However, many studies contain cohorts of unrelated individuals that share only short IBD segments. New sequencing technologies facilitate identification of short IBD segments through rare variants, which convey more information on IBD than common variants. Current IBD detection methods, however, are not designed to use rare variants for the detection of short IBD segments. Short IBD segments reveal genetic structures at high resolution. Therefore, they can help to improve imputation and phasing, to increase genotyping accuracy for low-coverage sequencing and to increase the power of association studies. Since short IBD segments are further assumed to be old, they can shed light on the evolutionary history of humans. We propose HapFABIA, a computational method that applies biclustering to identify very short IBD segments characterized by rare variants. HapFABIA is designed to detect short IBD segments in genotype data that were obtained from next-generation sequencing, but can also be applied to DNA microarray data. Especially in next-generation sequencing data, HapFABIA exploits rare variants for IBD detection. HapFABIA significantly outperformed competing algorithms at detecting short IBD segments on artificial and simulated data with rare variants. HapFABIA identified 160 588 different short IBD segments characterized by rare variants with a median length of 23 kb (mean 24 kb) in data for chromosome 1 of the 1000 Genomes Project. These short IBD segments contain 752 000 single nucleotide variants (SNVs), which account for 39% of the rare variants and 23.5% of all variants. The vast majority—152 000 IBD segments—are shared by Africans, while only 19 000 and 11 000 are shared by Europeans and Asians, respectively. IBD segments that match the Denisova or the Neandertal genome are found significantly more often in Asians and Europeans but also, in some cases exclusively, in Africans. The lengths of IBD segments and their sharing between continental populations indicate that many short IBD segments from chromosome 1 existed before humans migrated out of Africa. Thus, rare variants that tag these short IBD segments predate human migration from Africa. The software package HapFABIA is available from Bioconductor. All data sets, result files and programs for data simulation, preprocessing and evaluation are supplied at http://www.bioinf.jku.at/research/short-IBD.

## INTRODUCTION

A DNA segment is ‘identical by state (IBS)’ in two or more individuals if they have identical nucleotide sequences in this segment. An IBS segment is ‘identical by descent (IBD)’ in two or more individuals if they have inherited it from a common ancestor, that is, the segment has the same ancestral origin in these individuals. Rare variants can be used for distinguishing IBD from IBS without IBD because independent origins are highly unlikely for such variants. In other words, IBS generally implies IBD for rare variants, which is not true for common variants [(1), Ch. 15.3, p. 441].

Current IBD methods reliably detect long IBD segments because many minor alleles in the segment are concordant between the two haplotypes under consideration. However, many cohort studies contain unrelated individuals, which share only short IBD segments. Short IBD segments contain too few minor alleles to distinguish IBD from random allele sharing by recurrent mutations, which corresponds to IBS, but not IBD. New sequencing techniques provide rare variants, which facilitate the detection of short IBD segments. Rare variants convey more information on IBD than common variants because random minor allele sharing is less likely for rare variants than for common variants (2).

Short IBD segments resolve genetic structures on a fine scale and, therefore, provide important additional information for various applications in genetics. For example, the imputation of missing single nucleotide variants (SNVs) in genotype data (3,4) and haplotype phasing could be improved (5). Short IBD segments that are characterized by rare variants can increase genotyping accuracy obtained from low-coverage sequencing (6–10). The low power of association tests between diseases and rare variants (11,12) can be increased by using short IBD segments. They can serve to group SNVs to reduce the number of hypotheses or be directly used to test for an association (13–19). Moreover, short IBD segments can be assumed to be old compared with long IBD segments, which provides valuable insights in the field of population genetics. Sharing of short IBD segments between populations and the distribution of their lengths allow to investigate the evolutionary and the demographic history of humans (20,21).

Most IBD detection methods are based on hidden Markov models (HMMs) in which, at each DNA locus, a hidden state indicates presence or absence of IBD. HMM-based IBD methods are implemented in software tools like PLINK (22), RELATE (23) and BEAGLE (24). The phasing method fastPHASE (25) internally constructs IBD segments by using HMMs. The fastIBD method of the BEAGLE software package (26) uses HMMs for scoring matching alleles between two haplotypes. FastIBD first detects hot spots of matching DNA regions and then extends them, which is the basic idea of the previously published very fast IBD detection method GERMLINE (27). For related individuals, IBD detection methods can be enhanced by using pedigree information, where IBD segment sharing can be found in more than two individuals (28–30). Most IBD methods are not robust against genotyping errors and are computationally expensive for larger cohorts, as they must test all pairs of individuals for IBD. However, the main problem with current IBD detection methods is that they reliably detect long IBD segments (longer than 1 cM), but fail to distinguish IBD from identity by state (IBS) without IBD at short segments.

To detect short IBD segments, both the information supplied by rare variants and the information from IBD segments that are shared by more than two individuals should be used (2). The probability of a segment being IBD is typically computed via the probabilities of randomly sharing single alleles within the segment, where linkage disequilibrium (LD) may be taken into account (for an investigation to what extend LD helps to identify short IBD segments see the Supplementary Information, Section S7). The probability of randomly sharing a segment depends (a) on the allele frequencies within the segment, where lower frequency means lower probability of random sharing, and (b) on the number of individuals that share the allele, where more individuals result in lower probability of random segment sharing. The shorter the IBD segments, the higher the likelihood that they are shared by more individuals (see Supplementary Information, Section S4). Therefore, we focus on short IBD segments. There exists a trade-off between low minor allele frequency (MAF) versus many individuals having a segment (see Supplementary Information, Section S5). Consequently, a segment that contains rare variants and is shared by more individuals has higher probability of representing IBD (31,32). These two characteristics are our basis for detecting short IBD segments.

IBD detection using multiple individuals has been proposed for genotyping data with pedigree information (13,31,33). For IBD detection without pedigrees, the extensions of standard HMM algorithms that consider multiple individuals are computationally intractable due to the large state spaces (34). DASH (35) integrates multiple individuals into IBD clusters, which are found by clustering IBD detection results from GERMLINE. However, the performance of DASH depends mainly on the preceding IBD detection, which fails for short IBD segments. Only Moltke *et al.*’s (34) Markov Chain Monte Carlo-based method (MCMC) considers multiple individuals simultaneously during IBD detection. Moltke *et al.* (34) showed that multiple individuals improve IBD detection, as the MCMC method determined IBD segment break points more precisely and found shorter IBD segments with higher accuracy than other IBD methods. However, the MCMC method is based on a sampling technique and is therefore computationally expensive.

**Figure 1.**
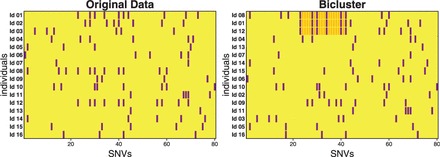
Biclustering of a genotyping matrix. Left: original genotyping data matrix, where rows give the individuals and columns consecutive SNVs. If at least one minor allele is present, then this is indicated by a violet bar for each individual–SNV pair, otherwise the bar is yellow. Right: after reordering the rows, a bicluster can be seen at the top three individuals. They contain the same IBD segment (in gold) and, therefore, are similar to each other by sharing minor alleles of SNVs within the segment (the tagSNVs).

We propose biclustering (36) to detect very short IBD segments that are shared among multiple individuals. Biclustering simultaneously clusters rows and columns of a matrix. In particular, it clusters row elements that are similar to each other on a subset of column elements. A genotype matrix has individuals (unphased) or chromosomes (phased) as row elements and SNVs as column elements. Entries in the genotype matrix usually count how often the minor allele of a particular SNV is present in a particular individual. Alternatively, minor allele likelihoods or dosages may be used (see Supplementary Information, Section S6). Individuals that share an IBD segment are similar to each other because they also share minor alleles of SNVs (tagSNVs) within the IBD segment (see Figure 1). Individuals that share an IBD segment represent a bicluster. Identifying a bicluster means identifying tagSNVs (column bicluster elements) that tag an IBD segment and, simultaneously, identifying individuals (row bicluster elements) that possess the IBD segment.

In contrast to standard IBD detection methods for unrelated individuals (except the MCMC method), biclustering considers multiple individuals. Biclustering performs well even if few individuals are similar to each other, e.g. for few occurrences of the minor allele of tagSNVs or, equivalently, for rare variants. Analogously, biclustering works well for few tagSNVs, i.e. for very short IBD segments. In contrast to standard clustering, biclustering allows for SNVs or individuals that do not belong to any cluster or that belong to more than one bicluster. Multi-cluster membership is advantageous for IBD detection because diploid individuals can have two IBD segments at one locus and an SNV may tag more than one IBD segment. An SNV that belongs to a bicluster tags the according IBD segment and an individual that belongs to a bicluster possesses this IBD segment. In summary, biclustering is well suited for detecting very short IBD segments in multiple individuals that are tagged by rare variants.

We have developed HapFABIA for identifying very short IBD segments. HapFABIA first applies Factor Analysis for Bicluster Acquisition (FABIA) biclustering to genotype data to detect identity by state (IBS) and then extracts IBD segments from FABIA results by distinguishing IBD from IBS without IBD. In contrast to other biclustering models, FABIA models are able to represent homozygous regions (same IBD segment in both chromosomes) and overlapping IBD segments (a different IBD segment in each chromosome at a locus). We compared HapFABIA with other IBD detection methods using artificial and simulated genotype data with implanted IBD segments and applied HapFABIA to sequencing data from the 1000 Genomes Project.

## MATERIALS AND METHODS

We present the HapFABIA method, which extracts short IBD segments that are tagged by rare variants from large sequencing data. The following two subsections describe the two stages of the HapFABIA method. In the first stage, HapFABIA applies FABIA biclustering to phased or unphased genotype data. Biclustering extracts individuals that share minor alleles (are similar to each other), that is, it detects identity by state (IBS). In the second stage, HapFABIA extracts IBD segments from FABIA models by distinguishing IBD from IBS without IBD. Finally, HapFABIA prunes spuriously correlated SNVs from the extracted IBD segments and joins segments.

### FABIA for genotype data

We propose identifying similarities between individuals by biclustering. Biclustering simultaneously clusters rows and columns of a matrix. More specifically, it clusters row elements that are similar to each other on a subset of column elements. An IBD segment corresponds to a bicluster because individuals that possess the IBD segment are similar to each other at this segment. The similarity is given by identical minor alleles of tagSNVs. Figure 1 depicts a bicluster identified in genotype data.

**Figure 2.**
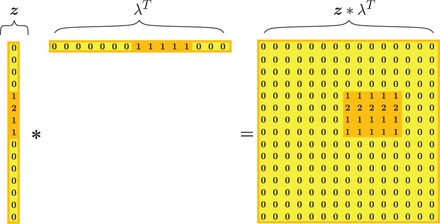
The outer product 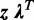 of vectors 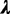 and 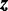. 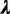 indicates IBD segment tagSNVs and 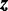 how many chromosomes of an individual contain the IBD segment. The row containing 2s indicates a homozygous region represented by 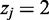 (two times the same IBD segment in individual *j*).

We use the ‘FABIA’ biclustering model (36). In contrast to other biclustering methods such as BIMAX (37) and QUBIC (38), FABIA can represent homozygous regions where the same IBD segment may be present in one diploid individual two times. As described below and depicted in Figure 2, the FABIA model has a variable that describes zygosity, i.e. how many chromosomes of an individual contain a particular IBD segment. FABIA can be applied to discrete phased or unphased genotype data, but also to real values that correspond to minor allele likelihoods or to minor allele dosages (see comparisons of results based on genotype, haplotype, likelihood and dosage in the Supplementary Information, Section S6). We use FABIA not only because it is well suited for genotyping data, but also because it outperformed other biclustering methods in extensive comparisons on different data sets (36).

### FABIA describes genotype data by IBD segments

FABIA describes an IBD segment in genotype data 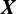 by an outer product 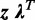 of two vectors 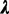 and 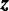, where the vector 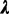 indicates tagSNVs by nonzero values and the vector 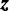 indicates individuals that possess the IBD segment. FABIA can represent a homozygous region of an IBD segment by *z* = 2, that is, two occurrences of an IBD segment in one diploid individual. Figure 2 visualizes this description of a genotype matrix by one IBD segment as an outer product.

A diploid individual may also possess two different IBD segments at a particular locus where genotyping sums up the occurrences of minor alleles. This fact is reflected by the FABIA model, which sums up bicluster contributions. If we assume genotyping errors that are accounted for by a noise term 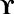, the FABIA model for genotype data 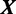 is
(1)
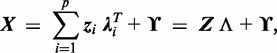

where 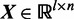 is the genotyping data; 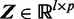 is the matrix that indicates which individuals possess an IBD segment; 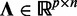 indicates IBD segment tagSNVs; 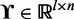 is an additive noise term; *n* is the number of SNVs; *l* is the number of individuals (or chromosomes for phased genotypes); *p* is the number of IBD segments; 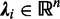 is the tagSNV indicator vector for the *i*-th IBD segment (the *i*-th row of 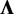); and 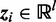 corresponds to the number of times each of the *l* individuals contains the *i*-th IBD segment (the *i*-th column of 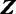). The additive noise 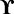 not only covers genotyping errors but also genotypes that cannot be explained by IBD segments. Such unexplained genotypes may arise from recently acquired SNVs, IBD segments observed in only one individual and IBD segments that are missed.

As illustrated in Figure 2, both the vector 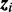 and the vector 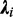 should be sparse to describe an IBD segment. Sparse 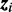 means that only few individuals possess the IBD segment, which implies rare tagSNVs. Sparse 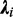 means that only few SNVs are tagSNVs, which implies short IBD segments. See Supplementary Information, Section S2, for the interpretation of 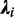 and 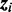 in the context of identifying short IBD segments in genotype data. In contrast to standard factor analysis, FABIA’s model selection is tailored to sparse factors and sparse parameters (36), which are essential for IBD detection. Sparseness in the FABIA model is obtained by a component-wise independent Laplace distribution both for the prior on the parameters 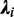 and for the distribution of the counts 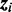. However, the Laplace distribution of the counts 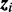 leads to an analytically intractable likelihood and posterior. Therefore, the model selection of FABIA is performed by variational expectation maximization (36,39–43). See Supplementary Information, Section S2, for more details on the FABIA method.

The number *p* of bicluster need not be determined a priori if *p* is chosen large enough. The sparseness constraint will remove a spurious bicluster *i* by setting 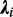 to the zero vector. In this way, FABIA automatically determines the number of biclusters.

### Adaptation of FABIA for IBD detection

Since an entry in the genotype matrix 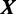 reports how often the minor allele is present, FABIA must explain occurrences of minor alleles by IBD segments.

- Nonnegativity constraints: The genotype matrix 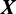 is nonnegative. The indicator matrix of tagSNVs 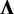 is 1, if the corresponding SNV is a tagSNV, and 0 otherwise. Thus, 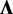 is also nonnegative. The matrix 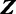 counts the number of occurrences of IBD segments in individuals or chromosomes. Consequently, 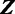 is nonnegative too. FABIA biclustering does not regard these nonnegativity constraints. For HapFABIA, we modified FABIA to ensure that the tagSNV indicator matrix 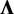 is nonnegative, which also implies that 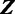 is nonnegative. See Supplementary Information, Section S2, for more details.
- Sparse matrix algebra for efficient computations: We exploit the sparsity of the genotype vectors (mostly the major allele is observed) and the sparsity of the indicator matrix 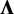 to speed up computations and to allow IBD segment detection in large sequencing data. We developed a specialized sparse matrix algebra that only stores and computes with nonzero values.
- Iterative biclustering for efficient computations: To further speed up the computation, we extended FABIA to an iterative version, where, in each iteration, *p* biclusters are detected. These *p* biclusters are removed from the genotype matrix 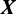 before starting the next iteration. The computational complexity of FABIA is 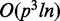, which means it is linear in the number of SNVs *n* and in the number of chromosomes or individuals *l*, but cubic in the number of biclusters *p*. The iterative version can extract 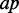 biclusters in 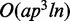 time instead of 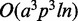 time of the original version of FABIA. For the 1000 Genomes Project, we used *a* = 40, which gave a speed up of 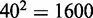.

### Extraction of IBD segments from FABIA models

FABIA biclustering detects identity by state (IBS) by finding individuals that are similar to each other by sharing minor alleles. In the second stage, HapFABIA distinguishes IBD from IBS without IBD. The idea is to find local accumulations of IBS SNVs, which indicate short IBD segments. IBD SNVs are within short IBD segments and, therefore, have small mutual physical distances. Then IBD segments are disentangled, pruned from spurious SNVs and finally joined if they are part of a long IBD segment.

For the separation of IBD from random IBS, it is important that the SNVs extracted by FABIA (the SNVs that are IBS) are independent of their physical location and their temporal order. Only if this independence assumption holds, statistical methods for identifying local SNV accumulations are justified. FABIA biclustering complies with this independence assumption because it does not regard the order of SNVs and random shuffling of SNVs does not change the results. Therefore, randomly correlated SNVs that are found by FABIA (SNVs that are IBS without IBD) would be uniformly distributed along the chromosome. However, SNVs that are IBS because they tag an IBD segment agglomerate locally in this segment. Deviations from the null hypothesis of uniformly distributed SNVs can be detected by a binomial test for the number of expected SNVs within an interval if the MAF of SNVs is known. A low *P*-value hints at local agglomerations of bicluster SNVs stemming from an IBD segment.

We propose a four-step procedure to extract IBD segments from FABIA models:

1. Identify local accumulations of IBS SNVs (SNVs detected by the FABIA model) by a binomial test since these accumulations distinguish random IBS from IBS caused by IBD;
2. Disentangle IBD segments and reassign individuals or chromosomes to IBD segments;
3. Prune IBD segments off SNVs with spuriously correlations based on an exponential test for long physical distances;
4. Join similar IBD segments stemming from long IBD segments that were divided at the first step during identifying accumulations.

**Step 1:** FABIA model selection is independent of the order of the SNVs. Therefore, spuriously correlated SNVs are unlikely to agglomerate at a DNA locus, whereas tagSNVs do, as they are within an IBD segment. To detect agglomerations, we compute histogram counts of FABIA model SNVs within bins that overlap by half of their length. Bins with large counts are assumed to contain IBD segments. For computing the histogram of counts of FABIA model SNVs, we consider those SNVs for which the FABIA model parameter 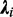 is largest (threshold ‘Lt’, with 10% being the default value). Large 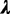 values ensure IBD segments that are shared by multiple individuals. These segments are, therefore, more reliable. The HapFABIA parameter ‘IBDsegmentLength’ determines the typical length of IBD segments. The histogram bin size in number of SNVs (all SNVs and not only model SNVs) is computed from ‘IBDsegmentLength’ using the average physical distance between adjacent SNVs.

The histogram bins with more model SNVs than expected by chance are assumed to contain IBD segments. We select bins for which the model SNV count exceeds the expected value by a binomial test for random counts. We need to compute how many model SNVs are expected to be in a bin if they are IBS, but not IBD. Thus, we have to compute the probability of observing *k* or more bin counts by chance. Let *p* be the probability of a random minor allele match between *t* individuals. If *n* SNVs are in a bin, the probability of observing *k* model SNVs by chance is given by
(2)
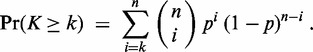



If *q* is the MAF for one SNV, the probability *p* of observing the minor allele of this SNV in all *t* individuals is 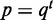. We assumed that all SNVs have the same MAF *q*—in the experiments we used the average MAF. For *b* bins, the probability of observing *k* or more counts of model SNVs by chance in at least one bin is
(3)
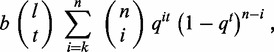

where *l* is the number of individuals and 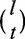 is the number of possibilities to chose *t* individuals from the *l* individuals. If the probability in Equation (3) is below the threshold ‘thresCount’, the according bin is selected for IBD segment extraction because more FABIA model SNVs are in this bin than expected by chance. If 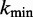 is the minimum *k* for which Equation (3) is below the threshold ‘thresCount’, then all bins with model SNV counts 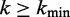 are selected. In our experiments, we allow for IBD segments that are observed in only two individuals (standard IBD), and therefore set *t* = 2.

If a bin is selected, SNVs and individuals must be assigned to it. Bicluster memberships of FABIA biclusters cannot be used directly because they include all bins and therefore different IBD segments. First, model SNVs are assigned to the selected bin if they contributed to its count. Then individuals or chromosomes are assigned to the selected bin if they possess a minor allele at one or more SNVs that have been assigned to the bin. Individuals are only chosen from the top *z*-values of the FABIA model to ensure that assigned individuals are similar to each other. The parameter ‘Zt’ (default 20%) gives the percentage of top *z*-scores that are considered.

In this step, we automatically distinguish between identity by state (IBS) and IBD. In particular, IBD can be distinguished from IBS without IBD by sharing of rare alleles because two independent origins are unlikely for them, so IBS generally implies IBD, which is not true for common alleles [(1), Ch. 15.3, p. 441]. The probability of IBS without IBD is given by (a) the probability of randomly observing minor allele sharing plus (b) the probability of observing recombined segments. In case (b), recombinations may be missed if a segment is broken via meiosis in one generation and then put together in later generations. Recombinations may also be missed if mother and father both have the same DNA segment. In both variants of case (b), IBS sharing in a segment is observed after intervening recombination and, therefore, this segment is not considered as a single IBD segment (44). For case (b), the lengths of IBS segments do not reflect their age because they are not IBD and, therefore, would misguide subsequent analyses. However, the case (b) has low probability if rare variants are considered. If the tagSNVs have low MAF, then the tagged segments cannot be observed frequently. The probability of observing a recombined segment is proportional to the MAF squared, which is 0.0025 for 5% MAF and 0.0001 for 1% MAF. This false-positive rate due to undetected recombinations is tolerable. Therefore, we only consider case (a) of random allele sharing. The probability of randomly observing *k* or more tagSNVs at *t* individuals simultaneously (IBS without IBD). This probability is given by Equation (3) without the factor *b*. Therefore, we distinguish IBS from IBD in this step.

If minimizing Equation (3), we observe a trade-off between small *q* and large *t* because 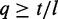. This trade-off is discussed in the Supplementary Information, Section S5. For rare variants, more individuals make random minor allele sharing (IBS without IBD) less likely.

**Step 2:** In this step, IBD segments in a selected bin are disentangled, where only SNVs and individuals are considered that have been assigned to the bin. An IBD segment is initialized by two core individuals that are identical at *m* or more minor alleles. The number *m* is computed as 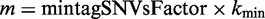, where 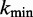 is computed in Step 1 and mintagSNVsFactor is a parameter with default value 3/4. All individuals that are identical in at least *m* minor alleles to one of the two IBD core individuals are classified as possessing the IBD segment. The tagSNVs of this IBD segment are model SNVs that have their minor allele in at least two individuals that possess the IBD segment.

Step 2 is repeated after removing the current IBD segment by deleting the segment’s tagSNVs until no more core individuals are found.

**Step 3:** This step prunes IBD segment borders of SNVs that have spurious correlations to the IBD segment. Spurious correlations may still be present in a bin leading to an overestimation of the segment length. Such SNVs can be identified by deviations of their MAFs from those of other tagSNVs. However, this criterion is not reliable for rare SNVs. Therefore, we identify SNVs with spurious correlations to an IBD segment on the basis of unusually large distances to other tagSNVs. The deviation from an expected distance is quantified by means of an exponential distribution with the median distance between tagSNVs as parameter. SNVs with distances leading to *P*-values below 1e-3 are removed. The two furthest upstream and the two furthest downstream tagSNVs are tested for their distances to other tagSNVs. If the second-furthest up- or downstream tagSNV is removed, then the furthest up- or downstream tagSNV is removed, too.

**Step 4:** IBD segments that are similar to each other are merged. In this way, long IBD segments that were divided by the bins into smaller parts are reconstructed. IBD segments greater than given by ‘IBDsegmentLength’ can be detected. To compute similarities, we assess how many tagSNVs and individuals of the smaller IBD segment are explained by the larger IBD segment. This criterion is expressed by the ‘overlap coefficient’
(4)
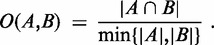



Using the overlap coefficient for both tagSNVs and individuals, we define a distance-like measure between IBD segments 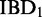 and 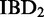 by
(5)
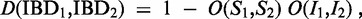

where *S_i_* and *I_i_* are the tagSNVs that tag IBD segment 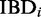 and individuals possessing IBD segment 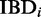, respectively. Using the measure *D*, IBD segments are clustered by hierarchical clustering using complete linkage. IBD segments are merged if their segments are clustered together below a cutting height of 0.8.

## RESULTS AND DISCUSSION

We first compare IBD detection methods on artificial and simulated sequencing genotype data sets where IBD segments are tagged by rare variants. The first data set contains artificial data. The second data set is based on genotype data from the 1000 Genomes Project into which real DNA segments are implanted to construct IBD segments. The third data set is based on genotype data obtained via a forward-time simulation into which IBD segments are implanted. Finally, we test IBD segment detection of HapFABIA on genotype data from the 1000 Genomes Project.

For all experiments and all compared methods the detailed command line calls, parameter settings, result filters and additional results can be found in the Supplementary Information, Section S8.

### Artificial and simulated genotype data

To compare IBD detection methods on artificial and simulated data, we first choose evaluation criteria that are described in the next subsection. Each of the following three subsections is devoted to comparisons on an artificial or simulated genotype data set. In each subsection, we first describe the data generation process and then report the results.

### Evaluation criteria

**Figure 3.**
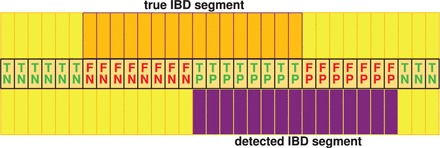
Evaluation of IBD detection methods. Each column is an SNV. The upper row shows a true IBD segment and the lower row a detected IBD segment. The middle row indicates TP, FP, TN and FN.

The primary measures used to evaluate IBD segment detection methods are power (sensitivity, true-positive rate, recall), false discovery rate (FDR) (1—precision) and computational complexity (2). Power can be increased by increasing the number of detections at the cost of a higher FDR and vice versa. Therefore, neither power nor FDR should be considered separately. A measure that combines both power and FDR is the F1 score. The F1 score is a standard performance measure in the field of information retrieval for measuring search performance, e.g. for finding documents. IBD segment detection is analogous to a document search, in which true IBD segments correspond to relevant documents and false IBD segments to nonrelevant documents. The F1 score is the harmonic mean of precision (1—FDR) and recall (power). Its maximal value of 1 is achieved for optimal detection, while its minimal value of 0 means that precision or recall were 0. We assess power, FDR and F1 score at the SNV (marker) level to take into account whether IBD segment lengths are under- or overestimated (2). Consequently, for each individual, SNVs that belong to an IBD segment are positives and all other SNVs are negatives. Analogously, SNVs that belong to a predicted IBD segment are predicted positives and all other SNVs are predicted negatives. Figure 3 shows true positives (TPs), false positives (FPs), true negatives (TNs) and false negatives (FNs) for a chromosome with a true IBD segment and a detected IBD segment. A perfect IBD detection method would detect all true IBD segments with correct break points and would not detect false IBD segments, thereby, yielding only TP and TN (100% power, 0% FDR and F1 score equal to 1). IBD detection methods as described in the introduction, except DASH, detect an IBD segment in a pair of chromosomes. For these methods, an IBD segment is detected in a chromosome if this segment is detected at least once (for at least one pair of chromosomes). Therefore, pairwise IBD detection methods are not penalized if they do not detect all IBD segments in all pairs of chromosomes.

We compare IBD detection methods on genotype data with known true IBD segments to evaluate the methods based on the ground truth. For both assessing the FDR and assessing the power of IBD detection methods, it is essential to know the positives, the true IBD segments (2).

Power, FDR and F1 score are given as the median over 100 experiments together with the *P*-value of a Wilcoxon rank sum test with the null hypothesis that HapFABIA and another method yield the same value. For reporting the results, the median and the Wilcoxon rank sum test are chosen because the results are in general not normally distributed (Shapiro-Wilk tests for normality). In contrast to normal assumptions, deviations of the results from their mean values are large because IBD segments are missed or falsely detected. The means (instead of the medians) of power, FDR and F1 score are reported in the Supplementary Information, Section S14.2.

### Artificial genotype data with IBD segments

First, we tried to simulate genotyping data by coalescent and forward population genetic modeling. However, current software packages (45–48) were not able to generate short IBD segments that are tagged by rare variants. Such short IBD segments do exist in real data—we could detect them in data of the 1000 Genomes Project (49) as well as in data of the Korean Personal Genome Project. We explored a wide range of different parameters including migration, population split, population join and different growth assumptions. Since standard genotype simulation models did not yield short IBD segments that are tagged by rare variants, we implanted IBD segments into genotype data.

For the first data set, we generated phased genotype data with rare variants (MAF <5%). Chromosomes are generated artificially, where alleles are in linkage equilibrium. To consider IBD detection with LD, in later experiments chromosomes are generated by forward simulation (see Subsection ‘Forward Simulation Genotype Data with Implanted IBD Segments’).

For the randomly generated chromosomes, the statistical characteristics (minor allele frequencies and distances between SNVs) were chosen to match the genotyping data from the 1000 Genomes Project. Minor alleles were chosen randomly according to the MAF. We implanted short IBD segments that are tagged by rare variants. The artificial genotype data consist of 100 and 1000 diploid individuals (200 and 2000 chromosomes) and 10 000 SNVs. The lengths of IBD segments were chosen to be very short, containing 100–200 SNVs on average, which corresponds to a length of 10–20 kb. This was motivated by the lengths of haplotype blocks (50,51). For example, Gabriel *et al.* (52) found that common haplotype blocks have an average length of 9 kb in Africans (AFR) and 18 kb in Europeans (EUR) and East Asians (ASN). Each IBD segment possesses a particular number of tagSNVs and is implanted in a certain number of chromosomes. More details on how the data are constructed can be found in the Supplementary Information, Section S3.1.

Table 1 provides the following information for each artificial genotype data set: the number of implanted IBD segments, the number of tagSNVs for an IBD segment, the number of chromosomes possessing an IBD segment, the minimal overlap of the implanted IBD segments between chromosomes (as they are broken at the end and beginning) and the number of mismatches that simulate genotyping errors.

**Table 1.** Overview of artificial data sets of phased genotype data

Data set	#I	L	#S	F	O	#M	#I
artA100	100	200	50	6	50	0	1
artA	1000	200	50	6	50	0	1
artAMis	100	200	50	6	50	6	1
artB100	100	200	20	10	100	0	1
artB	1000	200	20	10	100	0	1
artBMis	100	200	20	10	100	6	1
artC100	100	200	25	10	100	0	5
artC	1000	200	25	10	100	0	5
artCMis	100	200	25	10	100	6	5
artD100	100	100	20	10	50	0	20
artD	1000	100	20	10	50	0	20
artDMis	100	100	20	10	50	6	20

‘#I’ provides the number of diploid individuals, ‘L’ the length of the IBD segments in terms of the number of SNVs, ‘#S’ the number of tagSNVs for an IBD segment, ‘F’ shows how many chromosomes contain the IBD segment, column ‘O’ gives the minimal overlap of IBD segments between chromosomes, ‘#M’ lists the number of mismatches per IBD segment in a chromosome and ‘#I’ reports the number of different IBD segments that were implanted.

First, we assess the computational complexity of IBD detection methods. We recorded the computation times for different data sets and the following methods: HapFABIA, fastIBD (26), PLINK (22), GERMLINE (27), DASH (35), fastPHASE (25), RELATE (23) and MCMC (34). Table 2 reports the computational times. fastPHASE, RELATE and MCMC are not feasible for IBD detection in large data sets because of their extremely high computational complexity. To extract IBD segments from chromosome 1 in the data of the 1000 Genomes Project, RELATE would require 6.5 CPU years, and both fastPHASE and MCMC even more. GERMLINE and HapFABIA are the fastest IBD detection methods.

**Table 2.** Computation time in hours [h], minutes [min], seconds [s] required by IBD detection methods on data sets of various sizes

Number of individuals	100	100	1000	1000
Number of SNVs	10 000	100 000	10 000	200 000
Method				
HapFABIA	*31 s*	*5 min 43 s*	**6 min 12 s**	*3 min 2 s*
fastIBD	52 s	8 min 2 s	43 min 57 s	10 h 29 min
PLINK	1 min 47 s	18 min 12 s	2 h 59 min	67 h 14 min
GERMLINE	**5 s**	**52 s**	*8 min 17 s*	**36 s**
DASH	*22 s*	44 min 17 s	52 min 32 s	5 min 17 s
fastPHASE1	46 min 23 s	5 h 43 min	7 h 45 min	na
fastPHASE2	98 h 50 min	>490 h	>490 h	na
RELATE	53 min 2 s	10 h 43 min	89 h 12 min	na
MCMC	>564 h	Na	na	na

Computation times were recorded on a Linux machine with a 2.27 GHz Intel® Xeon® CPU. fastPHASE was called without fixing the number of clusters (fastPHASE) and with -K400 to fix 400 clusters (fastPHASE2)—DNA intervals that contain >400 IBD segments were often found in the 1000 Genomes Project. The first three data sets are artificial as explained above. The last data set (1000/200 000) is ‘simAlong’ from the experiments (see below). simAlong has a high proportion of private SNVs, which results in larger differences in run times. GERMLINE and HapFABIA are the fastest IBD detection methods. The Time of the fastest method per data set is given in bold face while the follow up time is in italics.

We compared the computationally feasible IBD detection methods HapFABIA, BEAGLE/fastIBD, PLINK, GERMLINE and DASH using the artificial data sets listed in Table 1. For fastIBD, we used calling thresholds of 1e-10 [fastIBD-1, this threshold was reported to give a small FDR (26)] and 1e-13 (fastIBD-2, our optimized value) instead of the default threshold of 1e-8 to reduce the FDR. For GERMLINE (with calling parameter bits = 30), we kept segments containing ≥150 SNVs (GERMLINE-1) and containing ≥200 SNVs (GERMLINE-2) to reduce the FDR—these lengths are found to be optimal for the implanted IBD segments. PLINK was called with segment-length of 0.1 and segment-snp of 20 to allow detection of short IBD segments. We optimized the parameters of the different methods, except for HapFABIA (see the Supplementary Information, Section S8). For HapFABIA, default parameters and the average MAF in the data set were used.

The average power of each method is given in Table 3. HapFABIA has significantly lower power than the methods with highest power. The reason is that HapFABIA is designed to have a low FDR, which is achieved at the cost of lower power. The average FDR of each method is given in Table 4. The FDRs of all methods, except for HapFABIA, are strikingly high. Hence, these methods are not appropriate for detecting short IBD segments. For example, in data set artA, PLINK (the second best method after HapFABIA) has an FDR of 0.996, which means 4 correct IBD segments out of 1000 detected, while HapFABIA has an FDR of 0.0679, which means 993 correct IBD segments out of 1000. artA is the data set, which is supposed to most closely resemble the 1000 Genomes Project data. To combine power and FDR in one value, the average F1 score of each method is reported in Table 5. The performance of HapFABIA did not decrease if sequencing errors are present. HapFABIA clearly and significantly outperformed the other methods due to its low FDR (Wilcoxon rank sum test was used to test significant performance differences).

**Table 3.** Comparison of IBD detection methods for short segments on artificial genotype data (phased) in terms of power (true-positive rate, sensitivity or recall)

Method	artA100		artB100		artC100		artD100	
	Median	*P*	Median	*P*	Median	*P*	Median	*P*
HapFABIA	0.87		0.72		0.79		0.56	
fastIBD-1	1.00	6e-16	0.17	6e-12	0.27	5e-18	0.20	4e-18
fastIBD-2	1.00	4e-13	0.00	3e-15	0.10	4e-18	0.05	4e-18
PLINK	0.98	4e-18	0.99	5e-18	0.99	4e-18	0.97	4e-18
GERMLINE-1	0.28	4e-18	0.84	9e-06	0.77	1e-01	0.32	4e-18
GERMLINE-2	0.13	4e-18	0.55	5e-02	0.49	7e-18	0.17	4e-18
DASH	0.20	4e-18	0.75	4e-18	0.71	4e-18	0.27	4e-18

Method	artAMis		artBMis		artCMis		artDMis	
	Median	*P*	Median	*P*	Median	*P*	Median	*P*
HapFABIA	0.86		0.64		0.81		0.56	
fastIBD-1	1.00	7e-17	0.21	1e-07	0.30	4e-18	0.19	4e-18
fastIBD-2	1.00	2e-12	0.00	3e-13	0.10	4e-18	0.06	4e-18
PLINK	0.98	2e-17	0.98	4e-18	0.99	4e-18	0.97	4e-18
GERMLINE-1	0.28	6e-18	0.81	8e-09	0.75	3e-02	0.32	4e-18
GERMLINE-2	0.12	4e-18	0.53	5e-01	0.46	5e-18	0.16	4e-18
DASH	0.22	4e-18	0.76	4e-08	0.69	4e-18	0.26	4e-18

Method	artA		artB		artC		artD	
	Median	*P*	Median	*P*	Median	*P*	Median	*P*
HapFABIA	0.91		0.50		0.83		0.53	
fastIBD-1	1.00	5e-18	1.00	6e-18	1.00	4e-18	0.99	4e-18
fastIBD-2	1.00	2e-13	0.99	4e-13	0.98	3e-17	0.93	4e-18
PLINK	0.99	1e-17	0.99	5e-18	0.99	4e-18	0.99	4e-18
GERMLINE-1	0.52	4e-13	0.87	6e-13	0.83	1e+00	0.51	3e-02
GERMLINE-2	0.31	4e-16	0.62	3e-03	0.62	2e-16	0.33	4e-18
DASH	0.47	4e-18	0.81	4e-18	0.79	4e-18	0.47	4e-18

Columns labeled ‘median’ show the median power over 100 experiments. Columns labeled ‘*P*’ provide the *P*-values of a Wilcoxon rank sum test over the 100 experiments with the null hypothesis that HapFABIA and another method yield the same value for the power. HapFABIA has significantly lower power than the methods with highest power. PLINK has the highest power.

**Table 4.** Comparison of IBD detection methods for short segments on artificial genotype data (phased) in terms of FDR (1—precision)

Method	artA100		artB100		artC100		artD100	
	Median	*P*	Median	*P*	Median	*P*	Median	*P*
HapFABIA	0.03		0.00		0.06		0.14	
fastIBD-1	0.98	4e-18	0.99	6e-18	0.95	4e-18	0.95	4e-18
fastIBD-2	0.91	4e-18	1.00	5e-18	0.86	4e-18	0.92	4e-18
PLINK	0.73	4e-18	0.63	6e-18	0.42	4 e-18	0.54	4 e-18
GERMLINE-1	0.999	4 e-18	0.997	4 e-18	0.99	4e-18	0.99	4e-18
GERMLINE-2	0.999	4e-18	0.997	4e-18	0.98	4e-18	0.99	4e-18
DASH	0.999	4e-18	0.997	4e-18	0.99	4e-18	0.99	4e-18

Method	artAMis		artBMis		artCMis		artDMis	
	Median	*P*	Median	*P*	Median	*P*	Median	*P*
HapFABIA	0.06		0.00		0.05		0.14	
fastIBD-1	0.98	4e-18	0.99	4e-18	0.95	4e-18	0.95	4e-18
fastIBD-2	0.88	4e-18	1.00	2e-18	0.87	4e-18	0.91	4e-18
PLINK	0.72	5e-18	0.62	4e-18	0.41	4e-18	0.53	4e-18
GERMLINE-1	0.999	4e-18	0.997	4e-18	0.99	4e-18	0.99	4e-18
GERMLINE-2	0.999	4e-18	0.996	4e-18	0.99	4e-18	0.99	4e-18
DASH	0.999	4e-18	0.997	4e-18	0.99	4e-18	0.99	4e-18

Method	artA		artB		artC		artD	
	Median	*P*	Median	*P*	Median	*P*	Median	*P*
HapFABIA	0.0679		0.0050		0.5059		0.5400	
fastIBD-1	0.9998	4e-18	0.9995	4e-18	0.9977	4e-18	0.9956	4e-18
fastIBD-2	0.9972	4e-18	0.9949	4e-18	0.9782	4e-18	0.9721	4e-18
PLINK	0.9960	4e-18	0.9919	4e-18	0.9611	4e-18	0.9302	4e-18
GERMLINE-1	0.9999	4e-18	0.9998	4e-18	0.9991	4e-18	0.9989	4e-18
GERMLINE-2	0.9999	4e-18	0.9998	4e-18	0.9991	4e-18	0.9990	4e-18
DASH	0.9999	4e-18	0.9998	4e-18	0.9991	4e-18	0.9990	4e-18

Columns labeled ‘median’ show the median FDR over 100 experiments. Columns labeled ‘*P*’ provide the *P*-values of a Wilcoxon rank sum test over the 100 experiments with the null hypothesis that HapFABIA and another method yield the same value for the FDR. HapFABIA has significantly lower FDRs. The FDRs of all methods, except for HapFABIA, are strikingly high.

**Table 5.** Comparison of IBD detection methods for short segments on artificial genotype data (phased) in terms of the F1 score

Method	artA100		artB100		artC100		artD100	
	Median	*P*	Median	*P*	Median	*P*	Median	*P*
HapFABIA	0.90		0.82		0.82		0.67	
fastIBD-1	0.04	4e-18	0.02	6e-16	0.09	4e-18	0.09	4e-18
fastIBD-2	0.19	4e-18	0.00	1e-15	0.11	4e-18	0.06	4e-18
PLINK	0.44	4e-18	0.54	8e-03	0.73	4e-14	0.63	2e-06
GERMLINE-1	0.00	4e-18	0.01	6e-16	0.03	4e-18	0.02	4e-18
GERMLINE-2	0.00	4e-18	0.01	6e-16	0.03	4e-18	0.02	4e-18
DASH	0.00	2e-17	0.01	6e-16	0.03	4e-18	0.02	4e-18

Method	artAMis		artBMis		artCMis		artDMis	
	Median	*P*	Median	*P*	Median	*P*	Median	*P*
HapFABIA	0.89		0.73		0.83		0.67	
fastIBD-1	0.04	4e-18	0.02	2e-15	0.09	4e-18	0.09	4e-18
fastIBD-2	0.17	4e-18	0.00	4e-15	0.11	4e-18	0.07	4e-18
PLINK	0.42	5e-18	0.54	1e-01	0.74	7e-13	0.63	3e-08
GERMLINE-1	0.00	4e-18	0.01	4e-15	0.03	4e-18	0.02	4e-18
GERMLINE-2	0.00	4e-18	0.01	4e-15	0.03	4e-18	0.02	4e-18
DASH	0.00	4e-18	0.01	4e-15	0.03	4e-18	0.02	4e-18

Method	artA		artB		artC		artD	
	Median	*P*	Median	*P*	Median	*P*	Median	*P*
HapFABIA	0.8466		0.5383		0.6166		0.4860	
fastIBD-1	0.0005	7e-18	0.0009	2e-13	0.0046	4e-18	0.0087	4e-18
fastIBD-2	0.0055	7e-18	0.0101	1e-13	0.0426	4e-18	0.0543	4e-18
PLINK	0.0079	7e-18	0.0160	2e-13	0.0748	4e-18	0.1305	4e-18
GERMLINE-1	0.0001	7e-18	0.0003	2e-13	0.0018	4e-18	0.0022	4e-18
GERMLINE-2	0.0000	7e-18	0.0003	2e-13	0.0018	4e-18	0.0019	4e-18
DASH	0.0001	7e-18	0.0003	2e-13	0.0018	4e-18	0.0021	4e-18

Columns labeled ‘median’ show the median F1 score over 100 experiments. Columns labeled ‘*P*’ provide the *P*-values of a Wilcoxon rank sum test over the 100 experiments with the null hypothesis that HapFABIA and another method yield the same value for the F1 score. The F1 score is the harmonic mean of precision (1—FDR) and power and has an optimal value of 1 for perfect IBD detection. HapFABIA performs significantly better than all other methods on all data sets except artBMis for which the performance of PLINK is not significantly worse.

### Sequencing data with implanted IBD segments

The second data set was constructed by implanting IBD segments into real phased sequencing data from chromosome 1 of the 1000 Genomes Project. Following (26), we destroyed existing IBD segments to assess false-positive rates. We implanted and then tried to rediscover short IBD segments of 10 or 20 kb. To ensure that discoveries other than the implanted IBD segments are false discoveries, we destroyed all IBD segments with a length of 5 kb or larger. For the same reason, for all methods detected, IBD segments that are <5 kb are discarded. Therefore, detected IBD segments are either implanted IBD segments (TPs) or false discoveries (FPs). For destroying IBD segments >5 kb, we divided chromosome 1 into blocks of 5 kb and then shuffled the sequential order of these blocks. The 5 kb blocks from the original data ensure that local LD still exists. Following (26,53), we copied IBD segments of one individual onto several other individuals. In contrast to previous experiments, we implanted very short IBD segments with a length of ∼0.01 cM (10 kb) and 0.02 cM (20 kb). Shuffling cannot be done completely at random because then, by chance, blocks that were close in the original chromosome could still be together in the shuffled chromosome. Methods can detect dependencies between such blocks, as their SNVs are correlated (e.g. LD exists). Therefore, we require blocks that were close in the original chromosome to be as far apart in the shuffled chromosome as possible. Similarly, we require that blocks that are close in the shuffled chromosome were far apart in the original chromosome. To achieve this, we applied a specific shuffling procedure, which is described in detail in the Supplementary Information, Section S3.2. In previous simulations (26,53), random segments were copied from one individual onto another individual. However, this procedure is not applicable because we must ensure that short IBD segments are tagged by rare variants to allow their detection (see ‘Introduction’ section). Another problem with previous simulations is that methods that consider multiple individuals may detect a strong IBD signal within a 5-kb block of an implanted segment. If multiple individuals are considered, a minor allele sharing (among many individuals) within a 5-kb block of an implanted segment may convey more information than a minor allele sharing (among few individuals) along the whole implanted segment. The procedure how segments are implanted into the shuffled chromosome is described in the Supplementary Information, Section S3.2.

We randomly selected 1000 individuals from the 1000 Genomes Project, selected implanted segments of length 10 and 20 kb and then shuffled the blocks of the chromosomes. From the shuffled chromosomes, we randomly selected a region with 10 000 SNVs. Into this region, we randomly implanted the IBD segments that were previously extracted from the original chromosome. We varied the length of the implanted IBD segment, the number of individuals that possess the IBD segment and the number of IBD segments that were implanted. Table 6 lists IBD segment lengths, numbers of chromosomes sharing the IBD segments and the number of implanted IBD segments for all data sets. For each data set, we generated 100 experiments, i.e. shuffled chromosomes with implanted IBD segments were randomly generated 100 times.

**Table 6.** Overview of phased sequencing data with implanted IBD segments

	L	F	#I		L	F	#I
impA	20	10	1	impD	10	10	20
impB	10	10	1	impE	20	10	5
impC	20	6	1				

‘L’ gives the length of the IBD segments in kb, ‘F’ shows how many chromosomes contain the IBD segment, ‘#I’ reports the number of different IBD segments that were implanted.

We applied the IBD detection methods to these data sets of shuffled real sequencing data with implanted IBD segments. Details on the parameters used for the methods are provided in the Supplementary Information, Section S3.2. For all methods, detected IBD segments that are <5 kb are discarded to assess the false detection rate (FDR). The power of each method is given in Table 7. PLINK has extremely high power followed by HapFABIA, GERMLINE-1 and DASH. Again HapFABIA trades high power against a lower FDR. The FDR of each method is given in Table 8. The FDR of HapFABIA is higher than in previous experiment because it detects IBD segments in the 5-bp blocks, which are overestimated due to random SNV correlations. Since many SNVs are rare, random correlations are more likely to be observed, which, in turn, leads to this overestimation. HapFABIA still has significantly lower FDRs than other methods. The FDRs of other methods but HapFABIA are too large to be feasible for IBD detection. The precision (1-FDR) of HapFABIA is >150 times larger than the precision of other methods. For data set impA, the best competitors had, on average, 4 correct IBD segments in 10 000 detections, while HapFABIA had 1242. The number of correct detections of the best competitors as compared with HapFABIA were as follows: for impB, HapFABIA detected 3549 out of 100 000 IBD segments, while the best competitor detected only 16; for impC, HapFABIA detected 726 out of 10 000 IBD segments, while the best competitor detected only 36; for impD, HapFABIA detected 5423 out of 10 000 IBD segments, while the best competitor detected only 33; for impE, HapFABIA detected 3907 out of 10 000 IBD segments, while the best competitor detected only 20. Data sets impD and impE are the most realistic data sets because they have multiple IBD segments. In these data sets, HapFABIA correctly detected 55 and 40% of the IBD segments. Again we combine power and FDR by the F1 score, which is reported for each method in Table 9. HapFABIA clearly and significantly outperformed the other methods due to its low FDR (Wilcoxon rank sum test was used to test for significant performance differences).

**Table 7.** Comparison of IBD detection methods for short segments on real phased sequencing data with implanted IBD segments in terms of power

Method	impA		impB		impC		impD		impE	
	Median	*P*	Median	*P*	Median	*P*	Median	*P*	Median	*P*
HapFABIA	0.8210		0.5062		0.7228		0.4112		0.6083	
fastIBD-1	0.1000	3e-11	0.0341	6e-08	1.0000	3e-14	0.0787	4e-18	0.1197	5e-18
fastIBD-2	0.0000	3e-14	0.0000	4e-10	1.0000	2e-09	0.0313	4e-18	0.0221	4e-18
PLINK	1.0000	2e-17	1.0000	2e-17	1.0000	4e-18	0.9893	4e-18	1.0000	4e-18
GERMLINE-1	0.7135	8e-01	0.4000	5e-01	0.6667	4e-02	0.4170	4e-01	0.6623	1e-02
GERMLINE-2	0.2399	6e-11	0.1000	8e-08	0.1077	8e-09	0.1544	5e-18	0.2698	2e-17
DASH	0.6621	3e-03	0.3743	3e-02	0.6233	6e-03	0.3686	4e-02	0.6222	4e-01

Columns labeled ‘median’ show the median power over 100 experiments. Columns labeled ‘*P*’ provide the *P*-values of a Wilcoxon rank sum test over the 100 experiments with the null hypothesis that HapFABIA and another method yield the same value for the power. PLINK has an extremely high power, followed by HapFABIA, GERMLINE-1 and DASH.

**Table 8.** Comparison of IBD detection methods for short segments on real phased sequencing data with implanted IBD segments in terms of FDR

Method	impA		impB		impC		impD		impE	
	Median	*P*	Median	*P*	Median	*P*	Median	*P*	Median	*P*
HapFABIA	0.8758		0.96451		0.92735		0.4577		0.6093	
fastIBD-1	0.9997	3e-14	0.99994	3e-08	0.99805	7e-10	0.9971	4e-18	0.9980	4e-18
fastIBD-2	1.0000	2e-14	1.00000	4e-10	0.99642	2e-10	0.9978	4e-18	0.9993	4e-18
PLINK	0.9996	8e-14	0.99984	8e-06	0.99981	9e-10	0.9967	4e-18	0.9984	4e-18
GERMLINE-1	0.9996	8e-14	0.99988	6e-06	0.99977	7e-10	0.9976	4e-18	0.9982	4e-18
GERMLINE-2	0.9997	6e-14	0.99993	4e-08	0.99993	2e-11	0.9980	4e-18	0.9984	4e-18
DASH	0.9999	2e-14	1.00000	5e-10	1.00000	2e-12	0.9985	4e-18	0.9991	4e-18

Columns labeled ‘median’ show the median FDR over 100 experiments. Columns labeled ‘*P*’ provide the *P*-values of a Wilcoxon rank sum test over the 100 experiments with the null hypothesis that HapFABIA and another method yield the same value for the FDR. HapFABIA has significantly lower FDRs than other methods. The FDRs of other methods than HapFABIA are too large for feasible IBD detection. The precision (1—FDR) of HapFABIA is >150 times larger than the precision of other methods.

**Table 9.** Comparison of IBD detection methods for short segments on real phased sequencing data with implanted IBD segments in terms of the F1 score

Method	impA		impB		impC		impD		impE	
	Median	*P*	Median	*P*	Median	*P*	Median	*P*	Median	*P*
HapFABIA	0.2124		0.0663		0.1337		0.4687		0.4707	
fastIBD-1	0.0006	6e-14	0.0001	3e-08	0.0039	7e-10	0.0055	4e-18	0.0039	4e-18
fastIBD-2	0.0000	2e-14	0.0000	3e-10	0.0071	2e-10	0.0041	4e-18	0.0014	4e-18
PLINK	0.0006	2e-13	0.0003	8e-06	0.0004	9e-10	0.0066	4e-18	0.0031	4e-18
GERMLINE-1	0.0008	2e-13	0.0002	6e-06	0.0005	7e-10	0.0048	4e-18	0.0036	4e-18
GERMLINE-2	0.0006	8e-14	0.0001	4e-08	0.0002	2e-11	0.0040	4e-18	0.0032	4e-18
DASH	0.0001	2e-14	0.0000	5e-10	0.0000	2e-12	0.0030	4e-18	0.0018	4e-18

Columns labeled ‘median’ show the median F1 score over 100 experiments. Columns labeled ‘*P*’ provide the *P*-values of a Wilcoxon rank sum test over the 100 experiments with the null hypothesis that HapFABIA and another method yield the same value for the F1 score. HapFABIA performs significantly better than all other methods on all data sets.

### Forward-simulation genotype data with implanted IBD segments

The third data set was constructed by implanting IBD segments into genotype data that has been generated by forward-time simulations. The data is phased per construction, as the forward-time simulation provides the chromosomes for each diploid individual. We compare the IBD detection methods also on long- and medium-sized IBD segments (0.5, 1 and 2 Mb).

In contrast to the previous data sets, the forward-time simulation ensures LD on a larger scale and evolutionary relationships between chromosomes. With the forward-time simulator SFS_CODE (45), we generated 147 DNA chunks of 300 kb length each. Following (54), we modeled a demographic history, which includes an ancient African expansion (∼177 thousand years ago = 177 kya), an out-of-Africa bottleneck (∼62 kya), a founding of Europe bottleneck (∼28 kya), an initial phase of exponential growth within Europe and a recent explosive growth phase (starting ∼5 kya). See more details in the Supplementary Information, Section S3.3.

Joining the 147 DNA chunks led to a DNA strand with 44 094 874 bases. We sampled 5000 individuals from the final population, which yielded 1 148 822 SNVs. For generating genotype data, we sampled 1000 individuals from the 5000, which gave, on average, 418 000 SNVs and an average distance of 105 bp between SNVs (for the 1000 Genomes Project, this distance is 78 bp). Next we selected an interval containing 10 000 SNVs (∼1 Mb length) for short IBD segments (10 and 20 kb) and an interval containing 200 000 SNVs (∼20 Mb length) for long- and medium-sized IBD segments (0.5, 1 and 2 Mb). Then we implanted IBD segments into the selected genotype data interval, where the IBD segments were taken from individuals that do not belong to the sampled 1000 individuals. Implantation was performed analogously to the previous experiment ‘Sequencing Data with Implanted IBD Segments’. We implanted IBD segments that had at least 8 tagSNVs. An IBD segment of length 1 Mb resulted in 140–250 tagSNVs and a length of 8000–10 000 SNVs. Table 10 gives an overview of the data sets that are characterized by the length of implanted IBD segments, number of different IBD segments implanted and how many chromosomes possess a particular IBD segment.

**Table 10.** Overview of data sets based on forward-time simulation

	L	F	#I		L	F	#I
simA	20	10	1	simAlong	1000	6	1
simB	10	10	1	simBlong	1000	2	1
simC	20	6	1	simClong	2000	6	1
simD	10	10	20	simDlong	2000	2	1
simE	20	10	5	simElong	500	6	1
				simFlong	500	2	1

‘L’ gives the length of the IBD segments in kb, ‘F’ shows how many chromosomes contain the IBD segment, ‘#I’ reports the number of different IBD segments that were implanted.

First we analyzed the data sets with implanted short IBD segments (simA–simE) by IBD detection methods. For details on the parameters used for the methods, see Supplementary Information, Section S8. For 10 kb long implanted IBD segments, we called GERMLINE with bits = 80 (seed in SNVs) and filtered: GERMLINE-1 with minimal length of 70 SNVs and GERMLINE-2 with 90 SNVs. For 20 kb long implanted IBD segments, we called GERMLINE with bits = 170 and filtered: GERMLINE-1 with minimal length of 150 SNVs and GERMLINE-2 with 180 SNVs. The average power of each method is given in Table 11. GERMLINE-1, DASH and HapFABIA have a high power. GERMLINE-2 has low power, as almost all implanted IBD segments are filtered out, though the filter is only slightly larger than the initial seed length. The high power of GERMLINE-1 and DASH is traded against a high FDR as shown in Table 12, which lists the average FDR for all methods. The FDR of HapFABIA is zero for 10 kb long IBD segments (simB and simD), but >50% for data sets into which only one 20 kb long IBD segment was implanted (simA and simC). HapFABIA has higher FDR for 20 kb long IBD segments because it often overestimated IBD segment lengths. HapFABIA still has significantly and considerably lower FDRs than other methods. Again the FDRs of all methods, except HapFABIA, are too large to be feasible for IBD detection. Again we combine power and FDR by the average F1 score, which is reported for each method in Table 13. HapFABIA clearly and significantly outperformed the other methods owing to its low FDR (Wilcoxon rank sum test was used to test for significant performance differences).

**Table 11.** Comparison of IBD detection methods for short segments on forward simulation data with implanted IBD segments in terms of power

Method	simA		simB		simC		simD		simE	
	m	*P*	m	*P*	m	*P*	m	*P*	m	*P*
HapFABIA	0.81		0.83		0.86		0.56		0.72	
fastIBD-1	0.10	5e-16	0.10	4e-12	0.50	2e-07	0.15	4e-18	0.15	4e-18
fastIBD-2	0.00	4e-16	0.00	4e-13	0.17	5e-14	0.06	4e-18	0.06	4e-18
PLINK	0.36	4e-08	0.04	3e-12	0.28	9e-11	0.12	4e-18	0.37	1e-17
GERMLINE-1	0.95	2e-11	0.92	2e-04	0.96	2e-10	0.78	6e-18	0.94	8e-18
GERMLINE-2	0.00	2e-15	0.00	2e-12	0.00	3e-15	0.00	4e-18	0.00	4e-18
DASH	0.94	9e-10	0.92	2e-04	0.93	4e-07	0.76	6e-18	0.91	2e-16

Columns labeled ‘m’ show the median power over 100 experiments. Columns labeled ‘*P*’ provide the *P*-values of a Wilcoxon rank sum test over the 100 experiments with the null hypothesis that HapFABIA and another method yield the same value for the power. GERMLINE-1, DASH and HapFABIA have a high power.

**Table 12.** Comparison of IBD detection methods for short segments on forward simulation data with implanted IBD segments in terms of FDR

Method	simA		simB		simC		simD		simE	
	Median	*P*	Median	*P*	Median	*P*	Median	*P*	Median	*P*
HapFABIA	0.5019		0.0000		0.6439		0.0000		0.1673	
fastIBD-1	0.9999	8e-17	0.9999	7e-18	0.9998	2e-17	0.9986	4e-18	0.9994	4e-18
fastIBD-2	1.0000	2e-16	1.0000	3e-18	0.9998	6e-17	0.9986	4e-18	0.9994	4e-18
PLINK	0.9994	7e-17	0.9997	6e-18	0.9997	2e-17	0.9864	4e-18	0.9969	4e-18
GERMLINE-1	0.9998	6e-17	0.9999	7e-18	0.9999	2e-17	0.9980	4e-18	0.9991	4e-18
GERMLINE-2	1.0000	2e-15	1.0000	9e-20	1.0000	5e-17	1.0000	2e-18	1.0000	9e-18
DASH	0.9998	6e-17	1.0000	9e-19	0.9999	2e-17	0.9984	4e-18	0.9991	4e-18

Columns labeled ‘median’ show the median FDR over 100 experiments. Columns labeled ‘*P*’ provide the *P*-values of a Wilcoxon rank sum test over the 100 experiments with the null hypothesis that HapFABIA and another method yield the same value for the FDR. HapFABIA has significantly lower FDRs than other methods. The FDRs of other methods than HapFABIA are too large to allow feasible IBD detection.

**Table 13.** Comparison of IBD detection methods for short segments on forward simulation data with implanted IBD segments in terms of the F1 score

Method	simA		simB		simC		simD		simE	
	Median	*P*	Median	*P*	Median	*P*	Median	*P*	Median	*P*
HapFABIA	0.5794		0.8421		0.3723		0.7136		0.7470	
fastIBD-1	0.0002	2e-16	0.0001	4e-12	0.0005	4e-16	0.0027	4e-18	0.0012	4e-18
fastIBD-2	0.0000	4e-16	0.0000	4e-12	0.0004	1e-15	0.0028	4e-18	0.0012	4e-18
PLINK	0.0012	2e-15	0.0005	8e-13	0.0006	3e-16	0.0245	4e-18	0.0062	4e-18
GERMLINE-1	0.0004	2e-16	0.0002	3e-11	0.0002	4e-16	0.0039	4e-18	0.0019	4e-18
GERMLINE-2	0.0000	2e-15	0.0000	2e-12	0.0000	3e-15	0.0000	4e-18	0.0000	4e-18
DASH	0.0004	2e-16	0.0002	3e-11	0.0002	4e-16	0.0032	4e-18	0.0018	4e-18

Columns labeled ‘median’ show the median F1 score over 100 experiments. Columns labeled ‘*P*’ provide the *P*-values of a Wilcoxon rank sum test over the 100 experiments with the null hypothesis that HapFABIA and another method yield the same value for the F1 score. HapFABIA performs significantly better than all other methods on all data sets.

In the last set of experiments, we implanted long- and medium-sized IBD segments of length 0.5, 1 and 2 Mb into the simulated genotype data. For details on the parameters used for the methods see Supplementary Information, Section S8. The average power of each method is given in Table 14. All methods have high power and are able to detect long IBD segments. Table 15 lists the average FDR for all methods. The FDR of PLINK is large, while all other methods have a low FDR for 1 and 2 Mb long IBD segments. For medium-sized IBD segments of 0.5 Mb, GERMLINE has a large FDR too. For medium-sized IBD segments, HapFABIA has significantly lower average FDR than other methods. Table 16 reports the average F1 score of the compared methods. PLINK performs significantly worse than other methods. GERMLINE performs better than fastIBD on long IBD segments but worse for medium-sized 0.5 Mb long IBD segments because of its large FDRs. For data sets simAlong and simClong (20 kb IBD segment length), there is no significant performance difference between the methods. However, for simBlong, simDlong, simElong and simFlong, HapFABIA has a significantly higher F1 score than other methods owing to its low FDR.

**Table 14.** Comparison of IBD detection methods for long segments on forward simulation data with implanted IBD segments in terms of power

Method	simAlong		simBlong		simClong		simDlong		simElong		simFlong	
	m	*P*	M	*P*	m	*P*	m	*P*	m	*P*	m	*P*
HapFABIA	0.98		0.98		0.99		0.99		0.97		0.96	
fastIBD-1	0.96	6e-03	0.81	1e-14	0.97	6e-06	0.83	1e-16	0.96	6e-01	0.80	3e-10
fastIBD-2	0.92	3e-11	0.62	2e-15	0.94	3e-14	0.70	8e-17	0.85	7e-02	0.70	3e-12
PLINK	1.00	2e-15	1.00	3e-16	1.00	9e-17	1.00	2e-16	1.00	6e-18	1.00	4e-18
GERMLINE-1	0.94	2e-10	0.93	5e-11	0.96	2e-09	0.95	8e-13	0.90	1e-06	0.88	3e-10
GERMLINE-2	0.94	2e-10	0.93	3e-10	0.96	2e-09	0.95	8e-13	0.91	4e-04	0.88	8e-10
DASH	0.93	2e-12			0.94	3e-12			0.00	4e-18		

Columns labeled ‘m’ show the median power over 100 experiments. Columns labeled ‘*P*’ provide the *P*-values of a Wilcoxon rank sum test over the 100 experiments with the null hypothesis that HapFABIA and another method yield the same value for the power. PLINK has an extremely high power, followed by HapFABIA. In summary, all methods have high power.

**Table 15.** Comparison of IBD detection methods for long segments on forward simulation data with implanted IBD segments in terms of FDR

Method	simAlong		simBlong		simClong		simDlong		simElong		simFlong	
	m	*P*	m	*P*	m	*P*	m	*P*	m	*P*	m	*P*
HapFABIA	0.000		0.00		0.000		0.000		0.00		0.000	
fastIBD-1	0.057	7e-02	0.02	2e-11	0.032	5e-02	0.003	7e-10	0.30	5e-18	0.577	2e-17
fastIBD-2	0.035	3e-01	0.00	1e-00	0.019	8e-02	0.000	1e-00	0.09	5e-14	0.005	4e-08
PLINK	0.987	4e-18	0.99	4e-18	0.975	4e-18	0.992	4e-18	1.00	4e-18	0.999	4e-18
GERMLINE-1	0.001	6e-02	0.00	1e-00	0.002	3e-01	0.000	1e-00	0.76	4e-18	0.910	4e-18
GERMLINE-2	0.001	6e-02	0.00	1e-00	0.002	4e-01	0.000	1e-00	0.96	4e-18	0.986	4e-18
DASH	0.000	3e-02			0.000	2e-02			0.00	1e-00		

Columns labeled ‘m’ show the median FDR over 100 experiments. Columns labeled ‘*P*’ provide the *P*-values of a Wilcoxon rank sum test over the 100 experiments with the null hypothesis that HapFABIA and another method yield the same value for the FDR. The FDR of PLINK is too large to be feasible for IBD detection. All other methods have a low FDR for long IBD segments (simAlong - simDlong). For medium-sized IBD segments (simElong, simFlong) GERMLINE has a large FDR. HapFABIA has significantly lower FDR for the medium-sized IBD segments (simElong and simFlong).

**Table 16.** Comparison of IBD detection methods for long segments on forward simulation data with implanted IBD segments in terms of the F1 score

Method	simAlong		simBlong		simClong		simDlong		simElong		simFlong	
	m	*P*	m	*P*	m	*P*	m	*P*	m	*P*	m	*P*
HapFABIA	0.98		0.99		0.99		0.99		0.98		0.98	
fastIBD-1	0.95	2e-01	0.83	5e-14	0.97	8e-02	0.89	1e-13	0.80	2e-17	0.54	4e-17
fastIBD-2	0.94	4e-02	0.74	5e-15	0.96	5e-02	0.82	3e-14	0.91	9e-11	0.80	2e-13
PLINK	0.03	6e-18	0.01	2e-17	0.05	4e-18	0.02	6e-18	0.00	4e-18	0.00	5e-18
GERMLINE-1	0.96	6e-01	0.97	4e-10	0.98	9e-01	0.97	2e-11	0.38	4e-18	0.16	5e-18
GERMLINE-2	0.96	6e-01	0.97	2e-09	0.98	9e-01	0.97	2e-11	0.08	4e-18	0.03	5e-18
DASH	0.96	3e-01			0.97	6e-01			0.00	4e-18		

Columns labeled ‘m’ show the median F1 score over 100 experiments. Columns labeled ‘*P*’ provide the *P*-values of a Wilcoxon rank sum test over the 100 experiments with the null hypothesis that HapFABIA and another method yield the same value for the F1 score. PLINK performs significantly worse than other methods. For data sets simAlong and simClong (20 kb IBD length), there is no significant performance difference between the methods. HapFABIA has slightly better results than GERMLINE, which in turn has slightly better results than fastIBD. However, for simBlong, simDlong, simElong and simFlong, HapFABIA has a significantly higher F1 score than other methods.

### IBD segments in data from the 1000 Genomes Project

We used HapFABIA to extract short IBD segments from the 1000 Genomes Project genotyping data (49), more specifically, the phase 1 integrated variant call set (version 1) containing phased genotype calls for SNVs, short indels and large deletions. This data set consists of 1092 individuals [246 AFR, 181 Admixed Americans (AMR), 286 East Asians and 379 EUR], 36.6 million SNVs, 3.8 million short indels and 14 000 large deletions. IBD detection was restricted to chromosome 1 to comply with the Ft. Lauderdale agreement for use of unpublished data for method development. Chromosome 1 contains 3 201 157 SNVs that are on average 78 bp apart and have an average MAF of 0.06. In all, 1 920 833 (60%) SNVs are rare 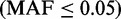, 684 171 (21.4%) are private (minor allele is observed only once), 15 124 (0.47%) have an MAF of zero and 581 029 (18.2%) are common (MAF > 0.05). We kept only the rare SNVs for IBD detection and excluded private ones.

Chromosome 1 was divided into intervals of 10 000 SNVs with adjacent intervals overlapping by 5000 SNVs. After removing common and private SNVs, we applied HapFABIA to these intervals. We used the same parameters as in the artificial and simulated data sets, but with more iterations (iter = 40) because more IBD segments were found per interval; the probability *q* from Equation (3) was estimated from the 1000 Genomes Project data. For more details on the parameters, see Supplementary Information, Section S8.

HapFABIA found 160 588 different very short IBD segments on chromosome 1. These contained 751 592 rare variants, which amounts to 39% of the rare variants and 23.5% of all SNVs. The distance between IBD segments had a median of 653 bp and a mean of 1.55 kb and ranged from 0 (overlapping IBD segments) to several Mb. The number of tagSNVs for an IBD segment ranged from 9 to 266, with a median of 11 and a mean of 15.5. The number of chromosomes that shared the same IBD segment was between 2 and 185, with a median of 6 and a mean of 13.5. The length of IBD segments ranged from 34 bp to 21 Mb, with a median of 23 kb and a mean of 24 kb. IBD lengths are computed as described in the Supplementary Information, Section S10.1, to match the assumptions for the distribution of IBD segment lengths as derived in other publications (24,33,55,56). A 20 kb long IBD segment corresponds to a common ancestor 50 kya (see Supplementary Information, Section S13.1, for the relation between IBD segment length and years from present). Therefore, the median length of 23 kb of IBD segments corresponds to 43.5 kya. That rare SNVs can be old is supported by a recent publication (57), which reports that the average origin of SNVs is 34.2 kya in EUR and 47.6 kya in AFR, while the SNVs shared between European Americans and African Americans date back 104.4 and 115.8 kya, respectively.

Next we characterize IBD segments with respect to their possible effects on biological functions. The ANNOVAR (58) software was used to annotate IBD segments as being within coding or promoter regions. In all, 13 796 IBD segments overlap with exons, 249 are near splice sites, 86 164 are intronic, 12 645 overlap with promoter regions (1 kb region upstream of the transcription start site), 111 998 are intergenic and the remaining are downstream, non-coding RNA (ncRNA) related or untranslated region 3 (UTR3)/UTR5 related. Out of the 13 796 exonic IBD segments, 30 contain a frameshift deletion, 171 a frameshift insertion, 2 a frameshift substitution, 9870 contain a nonsynonymous SNV, 179 a stopgain SNV, 12 a stoploss SNV and 9230 a synonymous SNV. An IBD segment can have more than one SNV of any of these categories. The tendency of observing more short IBD segments in introns or intergenic regions than in exons may be caused by a higher recombination rate in introns and intergenic regions. This would confirm other results on recombination rates (59). DNA regions close to exons may be subject to natural selection, which leads to less recombinations than in other regions.

We were interested in the distribution of IBD segments among different populations. The main population groups are AFR, ASN, EUR and AMR, where AMR consist of Colombian, Puerto Rican and Mexican individuals. Table 17 lists the number of IBD segments that are shared between particular populations. The vast majority (152 120) of the detected IBD segments are shared by AFR (at least one African possesses the segment), of which 93 197 are exclusively found in AFR. Only 19 062 and 10 645 IBD segments are shared by EUR and ASN, respectively. A total of 1191 IBD segments are exclusively found in EUR and 2522 exclusively in ASN. AMR share 384 IBD segments with ASN, but 1900 with EUR, which can be explained by the AMR admixture. If we additionally consider sharing with AFR, we obtain the same figures: 8322 IBD segments have AFR/AMR/EUR sharing, while only 1196 IBD segments have AFR/AMR/ASN sharing. According to results of the 1000 Genomes Project, individuals with African ancestry carry much more rare variants than those of European or Asian ancestry (49), supporting our finding that most IBD segments are shared by AFR. We found that few IBD segments are shared between two populations (Table 17 ‘Pairs of Populations’) confirming recently published results (49,60) (see also Supplementary Information, Section S12.1). The relatively large number of shared IBD segments between AFR and EUR was due to many shared IBD segments between the AFR subgroup ASW (Americans with African ancestry) and EUR. This tendency was also observed in the 1000 Genomes Project via the fixation index 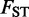 estimated by Hudson ratio of averages and via shared haplotype length around *f*_2_ variants (49). The high content of European DNA segments in ASW is consistent with the finding that in African Americans a median proportion of 18.5% is European (61). We conclude that IBD segments that are shared across continental populations, in particular by AFR, date back to a time before humans moved out of Africa. Consequently, the rare variants that tag these short IBD segments arose before the time humans migrated out of Africa. See Supplementary Information, Section S11, for a discussion of the question whether rare variants are recent or old.

**Table 17.** Number of IBD segments that are shared by particular populations

Single population					All populations
AFR	AMR	ASN	EUR		AFR/AMR/ASN/EUR
93 197	981	2522	1191		4132
Pairs of populations				Triplets of populations	
AFR/AMR	AFR/ASN	AFR/EUR		AFR/AMR/ASN	AFR/AMR/EUR
42 631	615	1720		1196	8322
AMR/ASN	AMR/EUR	ASN/EUR		AFR/ASN/EUR	AMR/ASN/EUR
384	1901	556		307	933

AFR = Africans (246), AMR = Admixed Americans (181), ASN = East Asians (286) and EUR = Europeans (379).

Since short IBD segments are thought to be ancient, we wondered whether some IBD segments match bases of primate genomes, such as chimpanzee and orangutan, or archaic genomes, such as Neandertal and Denisova. Ancient short IBD segments may reveal gene flow between archaic genomes and ancestors of modern humans and, thereby, shed light on different out-of-Africa hypotheses (62). Bases of the ancestral chimpanzee and orangutan genomes were given as additional information in the 1000 Genomes Project data. For the Denisova genome, sequencing data with a coverage of 31x was provided by the Max Planck Institute for Evolutionary Anthropology (63). Again we restricted our analysis to chromosome 1 to comply with the Ft. Lauderdale agreement for use of unpublished data for method development. Denisova bases were called by the software package SAMtools (64). Considering only the SNVs of the 1000 Genomes Project, 0.3% of the Denisova bases were not determined, 89.7% matched bases of the human reference and 10% matched either the human minor allele or were different from human alleles. The Neandertal genome (65) sequencing files were obtained from the European Bioinformatics Institute. Neandertal bases were again called by SAMtools but based on data with 1× coverage, resulting in lower quality than for the Denisova genome. At SNV loci of the 1000 Genomes Project, 33% of the Neandertal bases were not determined, 61% matched the human reference and 6% matched the human minor allele or were different from human alleles.

We tested whether IBD segments that match particular archaic genomes to a large extent are found more often in certain populations than expected randomly. For each IBD segment, we computed two values: The first value was the proportion of tagSNVs that match a particular archaic genome, which we call ‘genome proportion’ of an IBD segment (e.g. ‘Denisova proportion’). The second value was the proportion of individuals that possess an IBD segment and are from a certain population as opposed to the overall number of individuals that possess this IBD segment. We call this value the ‘population proportion’ of an IBD segment (e.g. ‘Asian proportion’). Consider the following illustrative examples. If an IBD segment has 20 tagSNVs of which 10 match Denisova bases with their minor allele, then we obtain 10/20 = 0.5 = 50% as the Denisova proportion. If an IBD segment is observed in six individuals of which four are AFR and two EUR, then the African proportion is 4/6 = 0.67 = 67% and the European proportion is 0.33 = 33%. A correlation between a genome proportion and a population proportion would indicate that this genome is overrepresented in this specific population. Pearson’s product moment correlation test and Spearman’s rank correlation test both showed highly significant correlations between Denisova genome and ASN, Denisova genome and EUR, Neandertal genome and ASN and Neandertal genome and EUR. However, correlation tests are sensitive to accumulations of minor effects. Therefore, we focused subsequently on strong effects, i.e. large values of genome proportions and large values of population proportions.

We define an IBD segment to match a particular archaic genome if the genome proportion is ≥30%. Only 10% of the Denisova and 6% of the Neandertal bases (∼10% of the called bases) match the minor allele of the human genome on average. Therefore, we require an odds ratio of 3 to call an IBD segment to match an archaic genome. We found many more IBD segments that match the Neandertal or the Denisova genome than expected randomly. This again supports the statement that the detected short IBD segments are old and some of them date back to times of the ancestors of humans, Neandertals and Denisovans. IBD segments that match the Denisova genome often match the Neandertal genome too, thus these segments cannot be attributed to either one of these genomes. Therefore, we introduce the ‘Archaic genome’ (genome of archaic hominids ancestral to Denisovan and Neandertal) to which IBD segments are attributed if they match both the Denisova and the Neandertal genome. In the Supplementary Information, Section S12.2, we show densities of population proportions for IBD segments that match a particular archaic genome and for those that do not match that genome.

**Figure 4.**
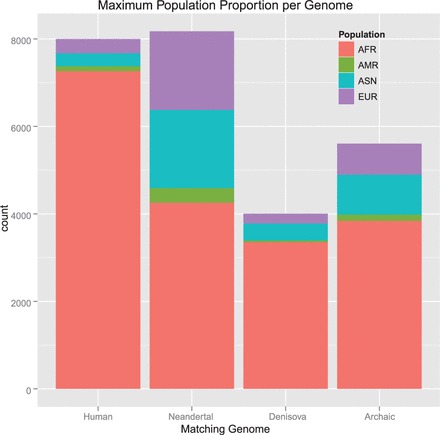
For each IBD segment, the population with maximum proportion is determined. IBD segments are given for each matching genome, where the color indicates the population that has maximum proportion. For the human genome, 8000 random IBD segments are chosen. Almost half of the Neandertal matching IBD segments have ASN or EUR as maximal population proportions. The Archaic genome (Neandertal and Denisovan) shows also an enrichment of IBD segments that are found mostly in ASN or EUR.

**Figure 5.**
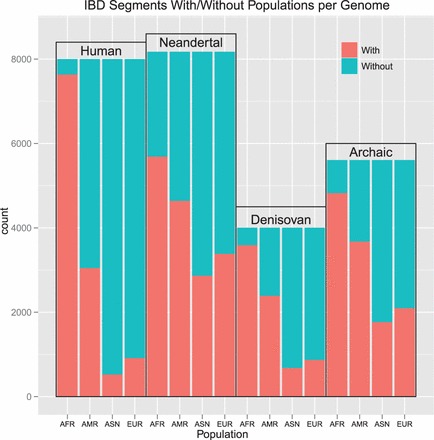
For each genome and each IBD segment, the color indicates whether a population contains this segment (‘With’) or not (‘Without’). For the human genome, 8000 random IBD segments are chosen. IBD segments that match the Neandertal or the Archaic genome are found more often in ASN and EUR than all IBD segments (human). This effect is not as prominent for IBD segments that match the Denisovan genome.

Next we investigated which population has a maximum proportion for an IBD segment that matches a particular genome—the population with the majority of the individuals possessing this segment. Figure 4 shows the population with maximum proportion for each IBD segment. The IBD segments are presented for each genome, where the colors show the populations with maximum proportion for the according IBD segment. Almost half of the Neandertal matching IBD segments have ASN or EUR as maximal population proportions. For the Archaic genome (intersection of Neandertal and Denisovan matching IBD segments), IBD segments dominated by ASN or EUR are also enriched if compared with all IBD segments found in chromosome 1 of the 1000 Genomes Project data (we call the set of these segments ‘human genome’). The enrichment by Asian or European IBD segments is lower for the Denisovan genome, but still significant (see tests in next paragraph). Next we asked which populations contain an IBD segment that matches a particular genome, that is, we asked whether this IBD segment is found in this population. Figure 5 shows for each genome (human and archaic) and each IBD segment whether a population contains this IBD segment. IBD segments that match the Neandertal or the Archaic genome are found more often in ASN and EUR than all IBD segments (human genome). This effect is not as prominent for IBD segments that match the Denisovan genome, but still significant (see tests in next paragraph).

We consider strong effects in terms of population proportions, where a considerable population proportion is ≥20%. Hence, a population has a considerable proportion of an IBD segment if 20% of the individuals that possess the IBD segment belong to this population. IBD segments were classified into (i) those that match or do not match a particular archaic genome and (ii) those that have or do not have a considerable proportion of a certain population. We tested whether these classes are related using Fisher’s exact test for count data. IBD segments matching the Denisova genome are enriched in the Asian (odds ratio of 4.7 with *P* < 1e-308) and the European population (odds ratio of 2.3 and *P* < 2.7e-152). Other thresholds lead to similar odds ratios and *P*-values (see Supplementary Information, Section S14.1). This confirms previous findings, where the authors discovered that European and Asian genomes are enriched by the Denisova genome if compared with AFR (63,66). IBD segments that match the Neandertal genome are enriched in ASN (odds ratio of 14.0 and *P* < 1e-308) and in EUR (odds ratio of 7.5 and *P* < 1e-308). Again, our results are in accordance with previous findings (65,67). In particular, Wall *et al.* (67) report that more Neandertal DNA is found in modern East Asians than in modern EUR. IBD segments that match an ancestral genome are enriched in ASN (odds ratio of 1.3 and *P* < 2.2e-08) and EUR (odds ratio of 1.5 and *P* < 2.1e-29). However, the ancestral (primate) genomes exhibit a considerable overlap with archaic hominid genomes potentially confounding matches with ancestral genomes. Thus, the results on matches with the ancestral genome must be considered with care.

**Figure 6.**
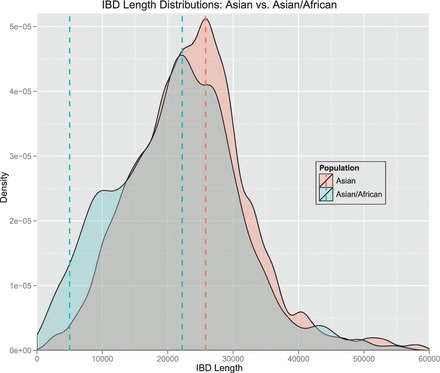
Density of lengths of IBD segments that are private to ASN versus density of IBD segment lengths shared only by ASN and AFR. The Asian global peak is at 25 800 bp (red dashed line), while the global peak for AFR-ASN is at 22 000 bp (blue dashed line). Interestingly, the African-Asian IBD segments are older as the higher density between 3000 and 10 000 bp (blue area) shows.

Next we investigate lengths distributions of IBD segments in different populations. Figure 6 shows the density of lengths of IBD segments that are private to ASN versus the density of IBD segment lengths shared only by ASN and AFR. The Asian global peak is at 25 800 bp (39 kya), while the global peak for AFR-ASN is at 22 000 bp (45.5 kya). The peak at 5000 bp (200 kya) marks the higher density range 3000–10 000 bp (333–100 kya). Thus, HapFABIA is able to reveal old human DNA segments. The peak at 5000 bp and the range 3000–10 000 bp always appear if IBD segments are shared by different populations that include AFR (see Supplementary Information, Section S13.4, Supplementary Figures S17 and S18). If only IBD segments are considered that are not shared by AFR, then the density of IBD lengths is increased between 35 000 bp (28.5 kya) and 55 000 bp (18 kya)—see Supplementary Information, Section S13.4, Supplementary Figure S18. More analyses on IBD segment lengths can be found in the Supplementary Information, Section S13.

We were also interested in lengths distributions of IBD segments that match archaic genomes, in particular, in the lengths of IBD segments between human and archaic genomes. The human IBD segment length is not an appropriate measure for the length of IBD segments between human and archaic genomes. For an IBD segment, we determined the part that matches an archaic genome to obtain the length of IBD between human and archaic genomes. Furthermore, we have to correct the number of generations for the archaic genomes, as they are not from present day, but from ∼40 kya. See Supplementary Information, Section S10.2, for these corrections of the IBD length to the length of IBD with archaic genomes. In the following, we present two examples of analyses, but more can be found in the Supplementary Information, Section S13.5.

**Figure 7.**
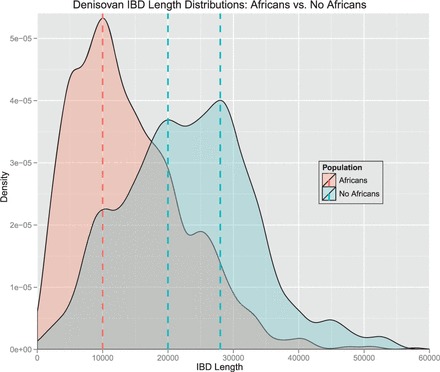
Densities of lengths of IBD segments that match the Denisova genome and are private to AFR versus IBD segments that are not observed in AFR. The peak for AFR is at 10 000 bp, while IBD segment lengths that are not observed in AFR have peaks at 20 000 and 28 000 bp.

Figure 7 shows densities of lengths of IBD segments that match the Denisova genome and are private to AFR versus IBD segments that are not observed in AFR. The peak for AFR is at 10 000 bp (120 kya), while the density of lengths of IBD segments that are not observed in AFR have peaks at 20 000 (70 kya) and 28 000 bp (56 kya). AFR have older segments probably stemming from common ancestors of Denisovans and humans. For the non-African populations, the high densities for longer IBD segments hint at an introgression from Denisovans after migration out of Africa. The Denisovan genome or parts of archaic genomes may also have been introduced by Neandertals after migration out of Africa. Neandertals may have reintroduced parts of archaic genomes that were lost in humans or parts of the Denisovan genome stemming from introgression of one hominid group into another.

**Figure 8.**
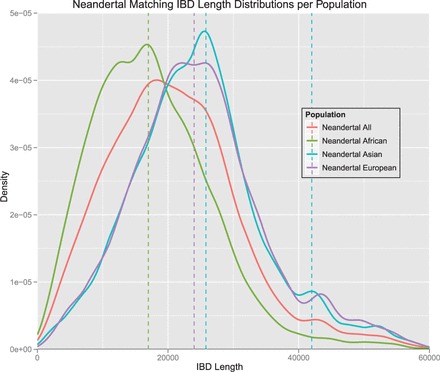
Densities of lengths of IBD segments that match the Neandertal genome and are enriched in a particular population. The dashed lines indicate the density peaks at 17 000 bp for AFR, 25 800 bp for ASN and 24 000 bp for EUR. Further, a smaller peak for both EUR and ASN is visible at 42 000 bp.

**Figure 9.**
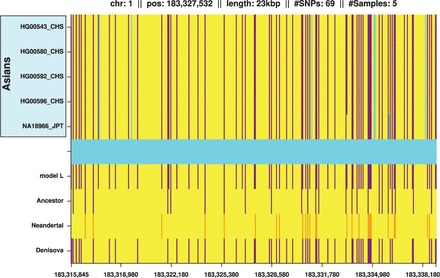
Example of an IBD segment matching the Denisova genome shared exclusively among ASN. The data analyzed by HapFABIA were phased genotypes from chromosome 1 of the 1000 Genomes Project. The rows give all chromosomes that contain the IBD segment and columns consecutive SNVs. If both chromosomes of an individual contain the IBD segment, then two adjacent identical row labels are present. Major alleles are shown in yellow, minor alleles of tagSNVs in violet and minor alleles of other SNVs in cyan. The row labeled ‘model L’ indicates tagSNVs identified by HapFABIA in violet. The rows ‘Ancestor’, ‘Neandertal’ and ‘Denisova’ show bases of the respective genomes in violet if they match the minor allele of the tagSNVs (in yellow otherwise). Neandertal tagSNV bases that are not called are shown in orange.

Figure 8 shows densities of lengths of IBD segments that match the Neandertal genome and are enriched in a particular population. The peak of the lengths distribution of African-matching IBD segments is at 17 000 bp (79 kya). ASN have a density peak at 25 800 bp (59 kya) and EUR a peak at 24 000 bp (62 kya). The densities of IBD segments that match the Neandertal genome have a peak ∼42 000 bp (44 kya) if they are private to EUR or to ASN. The density peak for AFR is clearly separated from the density peaks for EUR and ASN, which match each other well. This hints to introgression from the Neandertals into anatomically modern humans that were the ancestors of EUR and ASN after these humans left Africa. The higher density of short IBD segments, which are prominent in AFR in the range 5000–15 000 bp (220–87 kya), hints at old DNA segments that humans share with the Neandertal genome. A detailed analysis of lengths distributions is presented in the Supplementary Information, Section S13.5. Figure 9 shows a typical example of an IBD segment that matches the Denisova genome and is shared exclusively among ASN.

## CONCLUSION

We have introduced HapFABIA, a method for identifying very short IBD segments that are tagged by rare variants in large sequencing data. In artificial and simulated data, HapFABIA outperformed IBD detection methods such as BEAGLE/fastIBD, PLINK, GERMLINE and DASH. Using the chromosome 1 data from the 1000 Genomes Project, HapFABIA found 160 000 different short IBD segments, most of which were detected in AFR. Short IBD segments that match the Denisova genome are overrepresented in ASN and EUR. While some Denisova-matching IBD segments are exclusively shared among ASN, many are shared—in some cases exclusively—by AFR. Short IBD segments that match the Neandertal genome are overrepresented in ASN and EUR, but are also shared by AFR. HapFABIA is the first tool that can identify very short IBD segments in next-generation sequencing data—a topic which we expect to become increasingly important in genetics.

## SUPPLEMENTARY DATA

Supplementary Information is available at NAR Online.

## FUNDING

Funding for open access charge: European Union by an Industry-Academia Partnerships and Pathways (IAPP) project [324554] (Mr.SymBioMath) within the 7th framework program.

*Conflict of interest statement*. None declared.

## Supplementary Material

Supplementary Data
