# Supplementary material for: HapFABIA: Identification of very short segments of identity by descent characterized by rare variants in large sequencing data
Source: Nucleic Acids Res. 2013 Oct 29;41(22):e202. doi: 10.1093/nar/gkt1013 (PMC3905877; doi:10.1093/nar/gkt1013)
Supplement: Supplementary Data [file supp_41_22_e202__index.html]

HapFABIA: Identification of very short segments of identity by descent characterized by rare variants in large sequencing data — HapFABIA: Identification of very short segments of identity by descent characterized by rare variants in large sequencing data — Supplementary Data 

# HapFABIA: Identification of very short segments of identity by descent characterized by rare variants in large sequencing data

## Supplementary Data

files

**Files in this Data Supplement:**

- Supplementary Data - pdf file
