# Supplementary material for: HapFABIA: Identification of very short segments of identity by descent characterized by rare variants in large sequencing data
Source: Nucleic Acids Res. 2013 Oct 29;41(22):e202. doi: 10.1093/nar/gkt1013 (PMC3905877; doi:10.1093/nar/gkt1013)
Supplement: Supplementary Data [file supp_gkt1013_nar-00462-met-k-2013-File001.pdf]

HapFABIA: Identification of very short segments of identity by descent characterized by rare variants in large sequencing data
— *Supplementary Information* —

Sepp Hochreiter

Institute of Bioinformatics, Johannes Kepler University, Linz, Austria

Institute of Bioinformatics
Johannes Kepler University Linz
A-4040 Linz, Austria

Tel. +43 732 2468 4520
Fax +43 732 2468 4539
http://www.bioinf.jku.at

Contents

S1 Introduction 5
S2 FABIA Biclustering for Identifying IBD Segments in Genotype Data 5
S3 Data Generation 8
  S3.1 Artificial Genotype Data with IBD Segments 8
  S3.2 Sequencing Data with Implanted IBD Segments 9
  S3.3 Forward Simulation Genotype Data with Implanted IBD Segments 11
S4 How Many Individuals Share an IBD Segment? 13
S5 Small Likelihood of Identity by State: Small MAF vs. Many Individuals 14
S6 IBD Segments based on Likelihood and Dosage 15
S7 Identification of IBD Segment tagSNVs by Linkage Disequilibrium 16
S8 Calls, Filters, and Parameter Settings for the Methods 18
  S8.1 Commandline Calls 18
  S8.2 Arguments and Filters for the Different Data Sets 20
S9 Population Groups of the 1000 Genomes Project 25
S10 Calculation of IBD Lengths 26
  S10.1 Correction for the Assumptions of IBD Length Distributions 26
  S10.2 Length Correction for IBD with Archaic Genomes 26
S11 Are Rare Variants Recent or Old? 27
  S11.1 Some Rare Variants are Recent and Some Old 27
  S11.2 Old SNVs Have Been Reported in Other Publications 29
S12 Sharing of IBD Segments Between Populations and Genomes 30
  S12.1 Sharing of IBD Segments Between Continental Groups 30
  S12.2 IBD Segments that Match Archaic Genomes 30
S13 Analyses of Lengths of IBD Segments 37
  S13.1 Relating the IBD Length to Years from Present 37
  S13.2 Correction of the Length-Year Relation For Ancient Genomes 37
  S13.3 Histograms of Lengths for the Different Genomes 37
  S13.4 IBD Segment Lengths of Human Populations 39
  S13.5 Lengths of IBD Segments that Match the Denisova or Neandertal Genome . . 43
    S13.5.1 Lengths of IBD Segments that Match the Denisova Genome 43
    S13.5.2 Lengths of IBD Segments that Match the Neandertal Genome 44
    S13.5.3 Lengths of IBD Segments that Match an Archaic Genome 46
S14 Additional Results 49
  S14.1 Other Thresholds for Matching an Archaic Genome 49
  S14.2 Comparison Results Based on Mean Instead of the Median 49
S15 Examples of IBD Segments that Match Archaic Genomes 58

List of Figures

S1 Shuffling of blocks . . . 10
S2 Implanted segments . . . 12
S3 Population map of the 1000 Genomes Project . . . 25
S4 Age of SNVs according to Fu et al. . . . 29
S5 Density of Asian proportions of Denisova-matching IBD segments . . . 32
S6 Density of European proportions of Denisova-matching IBD segments . . . 32
S7 Density of African proportions of Denisova-matching IBD segments . . . 33
S8 Density of Asian proportions of Neandertal-matching IBD segments . . . 33
S9 Density of European proportions of Neandertal-matching IBD segments . . . 34
S10 Density of African proportions of Neandertal-matching IBD segments . . . 34
S11 Density of Asian proportions of Archaic-genome-matching IBD segments . . . 35
S12 Density of European proportions of Archaic-genome-matching IBD segments . . . 35
S13 Density of African proportions of Archaic-genome-matching IBD segments . . . 36
S14 Histogram human and Neandertal IBD segment lengths . . . 38
S15 Histogram Denisova and Archaic IBD segment lengths . . . 38
S16 IBD lengths private to Asians/Europeans . . . 39
S17 IBD lengths private to population vs. sharing with Africans . . . 40
S18 IBD lengths private to population vs. sharing with Africans . . . 41
S19 IBD lengths private to population vs. sharing with Africans . . . 42
S20 IBD segment lengths of Denisova matching segments . . . 43
S21 Population Specific IBD segment lengths for Denisova matching . . . 44
S22 IBD segment lengths of Neandertal matching segments . . . 45
S23 Population Specific IBD segment lengths for Neandertal matching . . . 46
S24 IBD segment lengths of Archaic matching segments . . . 47
S25 Population Specific IBD segment lengths for Neandertal matching . . . 48
S26 Example 1 of Denisova matching IBD segment . . . 59
S27 Example 2 of Denisova matching IBD segment . . . 60
S28 Example 3 of Denisova matching IBD segment . . . 61
S29 Example 1 of Neandertal matching IBD segment . . . 62
S30 Example 2 of Neandertal matching IBD segment . . . 63
S31 Example 3 of Neandertal matching IBD segment . . . 64
S32 Example 1: Denisova matching, Japanese, part . . . 64
S33 Example 3: Denisova matching, Japanese, part . . . 65

List of Tables

S1 Data sets forward-time simulation 13
S2 IBD segment matching genotype/dosage/likelihood 15
S3 Linkage disequilibrium 17
S4 Beagle/fastIBD filters 21
S5 PLINK arguments and filters 21
S6 GERMLINE parameters and filters 22
S7 Dash parameters and filters 23
S8 HapFABIA parameters and filters 24
S9 Population groups of the 1000 Genomes Project 25
S10 Comparison of IBD detection methods: simulated data, power, mean 50
S11 Comparison of IBD detection methods: simulated data, FDR, mean 51
S12 Comparison of IBD detection methods: simulated data, F1, mean 52
S13 Comparison of IBD detection methods: implanted data, power, mean 53
S14 Comparison of IBD detection methods: implanted data, FDR, mean 53
S15 Comparison of IBD detection methods: implanted data, F1, mean 54
S16 Comparison of IBD detection methods: forward simulation, power, mean 54
S17 Comparison of IBD detection methods: forward simulation, FDR, mean 55
S18 Comparison of IBD detection methods: forward simulation, F1, mean 55
S19 Comparison of IBD detection methods: forward simulation, long segments, power, mean 56
S20 Comparison of IBD detection methods: forward simulation, long segments, FDR, mean 56
S21 Comparison of IBD detection methods: forward simulation, long segments, F1, mean 57

S1 Introduction

This report provides the following supplementary information to the manuscript "HapFABIA: Identification of very short segments of identity by descent characterized by rare variants in large sequencing data":

- details on the FABIA method for identifying short IBD segments in genotype data
- details on generation of artificial and simulated data
- a section discussing how to obtain small likelihood of identity by state (IBS) without identity by descent (IBD): investigation of the trade-off between small minor allele frequency and many individuals sharing a segment
- comparisons of extracted IBD segments based on genotype, haplotype, dosage, and likelihood
- investigation to what extent linkage disequilibrium (LD) can help to identify IBD segments that are tagged by rare variants
- commandline calls, parameter settings, and result filters for each method and each experiment
- discussion whether rare variants are recent or old
- discussion of the result that IBD segments are shared between continental groups
- sharing of IBD segments between populations and genomes
- analyses of IBD lengths distributions
- additional results: other thresholds for matching an archaic genome and comparison results given by the mean instead of the median
- example IBD segments

S2 FABIA Biclustering for Identifying IBD Segments in Genotype Data

FABIA models each IBD segment in genotype data $\boldsymbol{X}$ by an outer product $\boldsymbol{z}\,\boldsymbol{\lambda}^T$ of two vectors $\boldsymbol{\lambda}$ and $\boldsymbol{z}$, where the vector $\boldsymbol{\lambda}$ indicates tagSNVs by non-zero values and the vector $\boldsymbol{z}$ indicates individuals that possess the IBD segment. FABIA can represent a homozygous region of an IBD segment by $z = 2$, that is, two occurrences of an IBD segment in one diploid individual. If we assume genotyping errors which are accounted for by a noise term $\boldsymbol{\Upsilon}$, the FABIA model for genotype data $\boldsymbol{X}$ is

$$\boldsymbol{X} \ = \ \sum_{i=1}^{p} \boldsymbol{z}_i \, \boldsymbol{\lambda}_i^T \ + \ \boldsymbol{\Upsilon} \ = \ \boldsymbol{Z}\,\boldsymbol{\Lambda} \ + \ \boldsymbol{\Upsilon} \ , \tag{S1}$$

where $\boldsymbol{X} \in \mathbb{R}^{l \times n}$ is the genotyping data; $\boldsymbol{Z} \in \mathbb{R}^{l \times p}$ is the matrix that indicates which individuals possess an IBD segment; $\boldsymbol{\Lambda} \in \mathbb{R}^{p \times n}$ indicates IBD segment tagSNVs; $\boldsymbol{\Upsilon} \in \mathbb{R}^{l \times n}$ is an additive noise term; n is the number of SNVs; l is the number of individuals (or chromosomes for phased genotypes); p is the number of IBD segments; $\boldsymbol{\lambda}_i \in \mathbb{R}^n$ is the tagSNV indicator vector for the i-th IBD segment (the i-th row of $\boldsymbol{\Lambda}$); and $\boldsymbol{z}_i \in \mathbb{R}^l$ corresponds to the number of times each of the l individuals contains the i-th IBD segment (the i-th column of $\boldsymbol{Z}$). The additive noise $\boldsymbol{\Upsilon}$ not only covers genotyping errors but also genotypes which cannot be explained by IBD segments. Such unexplained genotypes may arise from recently acquired SNVs, segments observed in only one individual, and IBD segments that are too short, or segments that are shared by too few individuals to be called.

According to Eq. (S1), the j-th genotype $\boldsymbol{x}_j$, i.e., the j-th column of $\boldsymbol{X}$, is

$$\boldsymbol{x}_j \ = \ \sum_{i=1}^{p} \boldsymbol{\lambda}_i \, z_{ij} \ + \ \boldsymbol{\epsilon}_j \ = \ \boldsymbol{\Lambda} \, \tilde{\boldsymbol{z}}_j \ + \ \boldsymbol{\epsilon}_j \ , \tag{S2}$$

where $\boldsymbol{\epsilon}_j$ is the j-th column of the noise matrix $\boldsymbol{\Upsilon}$ and $\tilde{\boldsymbol{z}}_j = (z_{1j}, \ldots, z_{pj})^T$ indicates which IBD segments are present in genotype $\boldsymbol{x}_j$ (j-th column of the matrix $\boldsymbol{Z}$ with length equal to the number of IBD segments p). Recall that $\boldsymbol{z}_i = (z_{i1}, \ldots, z_{il})^T$ in Eq. (S1) corresponds to the number of times each of the l individuals contains the i-th IBD segment.

If we drop the index j which indicates the individual, the formulation in Eq. (S2) facilitates a generative interpretation by a factor analysis model with p factors:

$$\boldsymbol{x} \ = \ \sum_{i=1}^{p} \boldsymbol{\lambda}_i \, \tilde{z}_i \ + \ \boldsymbol{\epsilon} \ = \ \boldsymbol{\Lambda} \, \tilde{\boldsymbol{z}} \ + \ \boldsymbol{\epsilon} \ , \tag{S3}$$

where $\boldsymbol{x} \in \mathbb{R}^n$ is the observed genotype. The vector of factors $\tilde{\boldsymbol{z}} = (\tilde{z}_1, \ldots, \tilde{z}_p)^T$ indicates which IBD segments are present in genotype $\boldsymbol{x}$, where component $\tilde{z}_i$ indicates how often the individual with genotype x possesses the i-th IBD segment. $\boldsymbol{\epsilon} \in \mathbb{R}^n$ is the additive noise, which is normally distributed according to $\mathcal{N}(\boldsymbol{0}, \boldsymbol{\Psi})$ with a diagonal covariance matrix $\boldsymbol{\Psi} \in \mathbb{R}^{n \times n}$. For large sample sizes and binary genotype data, the normal distribution approximates the binomial well (1, p. 130).

Both the vector $\boldsymbol{z}_i$ and the vector $\boldsymbol{\lambda}_i$ should be sparse to describe an IBD segment. Sparse $\boldsymbol{z}_i$ means that only few individuals possess the IBD segment, which implies rare tagSNVs. Sparse $\boldsymbol{\lambda}_i$ means that only few SNVs are tagSNVs, which implies short IBD segments. Sparse $\boldsymbol{z}_i$ can be achieved if components z_{ij} are sparse, that is, if the vector of factors $\tilde{\boldsymbol{z}}_j$ in Eq. (S2) is sparse or, equivalently, the vector $\tilde{\boldsymbol{z}}$ in Eq. (S3). In contrast to standard factor analysis, FABIA's model selection is tailored to sparse factors and sparse parameters, which are essential for IBD detection. Sparseness in the FABIA model is obtained by a component-wise independent Laplace distribution both for the prior on the parameters $\boldsymbol{\lambda}_i$ and for the distribution of the factors $\tilde{\boldsymbol{z}}$ (2):

$$p(\tilde{\boldsymbol{z}}) \ = \ \left(\tfrac{1}{\sqrt{2}}\right)^{p} \prod_{i=1}^{p} e^{- \sqrt{2} \, |\tilde{z}_i|} \tag{S4}$$

$$p(\boldsymbol{\lambda}_i) \ = \ \left(\tfrac{1}{\sqrt{2}}\right)^{n} \prod_{k=1}^{n} e^{- \sqrt{2} \, |\lambda_{ki}|} \ . \tag{S5}$$

The Laplace distribution of the factors leads to an analytically intractable likelihood:

$$p(\boldsymbol{x} \mid \boldsymbol{\Lambda}, \boldsymbol{\Psi}) \;=\; \int p(\boldsymbol{x} \mid \tilde{\boldsymbol{z}}, \boldsymbol{\Lambda}, \boldsymbol{\Psi})\, p(\tilde{\boldsymbol{z}})\, d\tilde{\boldsymbol{z}}\ . \tag{S6}$$

Therefore, the model selection of FABIA is performed by variational expectation maximization (EM) (3, 4, 5, 6, 7). The idea of the variational approach is to express the prior $p(\tilde{\boldsymbol{z}})$ by the maximum

$$p(\tilde{\boldsymbol{z}}) \;=\; \max_{\boldsymbol{\xi}} p(\tilde{\boldsymbol{z}} \mid \boldsymbol{\xi}) \tag{S7}$$

over a model family $p(\tilde{\boldsymbol{z}} \mid \boldsymbol{\xi})$ that is parametrized by the variational parameter $\boldsymbol{\xi}$ or by scale mixtures

$$p(\tilde{\boldsymbol{z}}) \;=\; \int p(\tilde{\boldsymbol{z}} \mid \boldsymbol{\xi})\, d\mu(\boldsymbol{\xi})\ . \tag{S8}$$

A Laplace distribution can be expressed exactly by the maximum of a Gaussian family or by Gaussian scale mixtures (3, 4). Therefore, for each $\boldsymbol{x}$, the maximum $\hat{\boldsymbol{\xi}}$ of the variational parameter $\boldsymbol{\xi}$ allows for representing the Laplacian prior by a Gaussian:

$$\hat{\boldsymbol{\xi}} \;=\; \arg\max_{\boldsymbol{\xi}} p(\boldsymbol{\xi} \mid \boldsymbol{x})\ . \tag{S9}$$

The maximum $\hat{\boldsymbol{\xi}}$ can be computed analytically (see Eq. (S12) below) because the likelihood Eq. (S6) can be computed analytically for each Gaussian.

Eqs. (S10)–(S15) below represent the iterative update rules of the FABIA EM algorithm. If we denote the j-th genotype by $\boldsymbol{x}_j \in \mathbb{R}^n$ with corresponding factors $\boldsymbol{z}_j \in \mathbb{R}^p$, then we obtain the following variational E-step (5):

$$\mathrm{E}\big(\boldsymbol{z}_j \mid \boldsymbol{x}_j\big) \;=\; \big(\boldsymbol{\Lambda}^T\, \boldsymbol{\Psi}^{-1}\, \boldsymbol{\Lambda} \;+\; \boldsymbol{\Xi}_j^{-1}\big)^{-1}\, \boldsymbol{\Lambda}^T\, \boldsymbol{\Psi}^{-1}\, \boldsymbol{x}_j\ , \tag{S10}$$

$$\mathrm{E}\big(\boldsymbol{z}_j\, \boldsymbol{z}_j^T \mid \boldsymbol{x}_j\big) \;=\; \big(\boldsymbol{\Lambda}^T\, \boldsymbol{\Psi}^{-1}\, \boldsymbol{\Lambda} \;+\; \boldsymbol{\Xi}_j^{-1}\big)^{-1} \;+\; \mathrm{E}\big(\boldsymbol{z}_j \mid \boldsymbol{x}_j\big)\, \mathrm{E}\big(\boldsymbol{z}_j \mid \boldsymbol{x}_j\big)^T\ , \tag{S11}$$

where $\boldsymbol{\Xi}_j$ stands for $\mathrm{diag}\,(\boldsymbol{\xi}_j)$. The update for the variational parameter $\boldsymbol{\xi}_j$ is

$$\boldsymbol{\xi}_j \;=\; \mathrm{diag}\left(\sqrt{\mathrm{E}(\boldsymbol{z}_j\, \boldsymbol{z}_j^T \mid \boldsymbol{x}_j)}\right)\ . \tag{S12}$$

The variational M-step is (5)

$$\boldsymbol{\Lambda}^{\mathrm{new}} \;=\; \frac{\frac{1}{l}\sum_{j=1}^{l} \boldsymbol{x}_j\, \mathrm{E}(\boldsymbol{z}_j \mid \boldsymbol{x}_j)^T \;-\; \frac{\alpha}{l}\, \boldsymbol{\Psi}\, \mathrm{sign}(\boldsymbol{\Lambda})}{\frac{1}{l}\sum_{j=1}^{l} \mathrm{E}(\boldsymbol{z}_j\, \boldsymbol{z}_j^T \mid \boldsymbol{x}_j)} \tag{S13}$$

$$\mathrm{diag}\,(\boldsymbol{\Psi}^{\mathrm{new}}) \;=\; \boldsymbol{\Psi}^{\mathrm{EM}} \;+\; \mathrm{diag}\Big(\frac{\alpha}{l}\, \boldsymbol{\Psi}\, \mathrm{sign}(\boldsymbol{\Lambda})(\boldsymbol{\Lambda}^{\mathrm{new}})^T\Big)\ , \tag{S14}$$

$$\boldsymbol{\Psi}^{\mathrm{EM}} \;=\; \mathrm{diag}\bigg(\frac{1}{l}\sum_{j=1}^{l} \boldsymbol{x}_j\, \boldsymbol{x}_j^T \;-\; \boldsymbol{\Lambda}^{\mathrm{new}} \frac{1}{l}\sum_{j=1}^{l} \mathrm{E}\,(\boldsymbol{z}_j \mid \boldsymbol{x}_j)\ \boldsymbol{x}_j^T\bigg). \tag{S15}$$

The parameter α controls the degree of sparseness (an expectation of how rare the tagSNVs are) and can be introduced as a parameter of the Laplacian prior of the factors (5).

The number of bicluster need not be determined a priori if p is chosen large enough. The sparseness constraint will remove spurious biclusters by setting $\boldsymbol{\lambda}$ to the zero vector. In this way, FABIA automatically determines the number of biclusters.

Non-negative factors and loading. We enforce FABIA to produce non-negative $\boldsymbol{\Lambda}$ and non-negative $\boldsymbol{Z}$ values, because indicator matrix $\boldsymbol{\Lambda}$ and count matrix $\boldsymbol{Z}$ are positive. The indicator matrix of tagSNVs $\boldsymbol{\Lambda}$ is 1, if the corresponding SNV is a tagSNV, and 0 otherwise. The matrix $\boldsymbol{Z}$ counts the number of occurrences of IBD segments in individuals or chromosomes. FABIA biclustering does not regard these non-negativity constraints. For HapFABIA, we modified FABIA to ensure that the tagSNV indicator matrix $\boldsymbol{\Lambda}$ is non-negative, which also implies that $\boldsymbol{Z}$ is non-negative. The matrix $\boldsymbol{Z}$ is given by the posterior means $\mathrm{E}(\boldsymbol{z}_j \mid \boldsymbol{x}_j)$ of $\boldsymbol{z}_j$. $\mathrm{E}(\boldsymbol{z}_j \mid \boldsymbol{x}_j)$ is non-negative according to Eq. (S10) with $\boldsymbol{\Xi}_j > 0$ and $\boldsymbol{\Psi}_{jj} > 0$ if both genotype data $\boldsymbol{x}_j$ and tagSNV indicator matrix $\boldsymbol{\Lambda}$ are non-negative. Consequently, the new tagSNV indicator matrix $\boldsymbol{\Lambda}^{\mathrm{new}}$ is non-negative according to Eq. (S13). The prior term $\frac{\alpha}{l}\ \boldsymbol{\Psi}\ \mathrm{sign}(\boldsymbol{\Lambda})$ in the update rule Eq. (S13) is not allowed to change the signs of the tagSNV indicator matrix $\boldsymbol{\Lambda}$ in one update step. Therefore, it is sufficient to initialize $\boldsymbol{\Lambda}$ by positive values to enforce non-negative $\boldsymbol{\Lambda}$ and $\boldsymbol{Z}$.

Sparse matrix algebra for efficient computations. Most entries of the genotype vectors $\boldsymbol{x}_j$ are zero because the major allele is observed. Most entries of the parameter matrix $\boldsymbol{\Lambda}$ are zero because only few SNVs become tagSNVs. We exploit this sparsity to speed up computations and to allow IBD segment detection in large sequencing data. We developed a specialized sparse matrix algebra which only stores and computes with non-zero values. The update rules in Eqs. (S10)–(S15) become much faster. In contrast to most existing sparse matrix software packages, our sparse matrix algebra allows for multiplying two sparse vectors.

Interpretation for analyzing genotype data. The vectors $\boldsymbol{\lambda}_i$ and $\boldsymbol{z}_i$ for the i-th bicluster obtain a new interpretation when detecting short IBD segments. Components of $\boldsymbol{\lambda}_i$ indicate how many individuals possess the corresponding tagSNV relative to individuals that possess the i-th IBD segment. Thus, $\lambda_{ij} = 1$ means the minor allele of j-th SNV is present in all individuals that possess the i-th IBD segment, while $\lambda_{ij} = 0.5$ it means the SNV is only present in 50% of individuals that possess the the i-th IBD segment. This means that components of $\boldsymbol{\lambda}_i$ give the quality of the corresponding tagSNV for detecting the i-th IBD segment. As mentioned previously, components of $\boldsymbol{z}_i$ indicate how often the i-th IBD segment is present in the corresponding multiploid individual. Additionally components of $\boldsymbol{z}_i$ show how well the DNA segment in an individual matches the i-th IBD segment. DNA segments in an individual may contain genotyping errors or may be broken up during meiosis, therefore, the i-th IBD segment need not be matched perfectly.

S3 Data Generation

S3.1 Artificial Genotype Data with IBD Segments

We created artificial phased genotype data with rare variants (MAF <5%) by randomly generating chromosomes. The statistical characteristics (minor allele frequencies and distances between SNVs) were chosen to match the genotyping data from the 1000 Genomes Project. We implanted short IBD segments that are tagged by rare variants. The artificial genotype data consist of 100 and 1,000 diploid individuals (200 and 2,000 chromosomes) and 10,000 SNVs. The average physical distance between adjacent SNVs is 100 bp in order to be close to the 1000 Genomes Project data,

for which the average is 78 bp. The distance between adjacent SNVs was drawn according to an exponential distribution to simulate a Poisson process. Rare variants were obtained by drawing their minor allele from a binomial distribution with a probability 1/25 (4%) resulting in a very sparse matrix with no coincidental IBD segments. Common SNVs were not considered, as we focus on IBD segments that are tagged by rare variants. This means that we generated a 200×10,000 or a 2,000×10,000 matrix. Then SNVs were randomly switched to their minor allele with a probability of 1/25 and IBD segments were implanted. The lengths of IBD segments were chosen to be very short, containing 100 to 200 SNVs on average, which correspond to a physical length of 10–20 kbp. The IBD segment lengths were motivated by the lengths of haplotype blocks (8, 9). For example, Gabriel et al. (10) found that common haplotype blocks have an average length of 9 kbp in Africans and 18 kbp in Europeans and Asians. Each IBD segment possesses a particular number of tagSNVs and is contained in a certain number of chromosomes. An implanted segment in a particular chromosome is shortened by randomly breaking up its beginning and end to simulate recombinations. For data sets artAMis, artBMis, artCMis, and artDMis, genotyping errors are simulated for each implanted segment by randomly switching alleles.

S3.2 Sequencing Data with Implanted IBD Segments

The second data set was constructed by implanting IBD segments into real sequencing data from chromosome 1 of the 1000 Genomes Project. Following Browning and Browning (11), we destroyed existing IBD segments to assess false positive rates. We implanted and then tried to rediscover short IBD segments of 10 or 20 kbp. To ensure that discoveries other than the implanted IBD segments are false discoveries, we destroyed all IBD segments with a length of 5 kbp or larger. For the same reason, detected IBD segments that are shorter than 5 kbp are discarded. Therefore, detected IBD segments are either implanted IBD segments (true positives) or false discoveries (false positives). For destroying IBD segments larger than 5 kbp, we divided chromosome 1 into blocks of 5 kbp and then shuffled the sequential order of these blocks. The 5 kbp blocks from the original data ensure that local linkage disequilibrium still exists. Following Browning and Browning (11) and Su et al. (12), we copied IBD segments of one individual onto several other individuals. In contrast to previous experiments, we implanted very short IBD segments with a length of around 0.01 cM (10 kbp) and 0.02 cM (20 kbp).

Shuffling cannot be done completely at random because then, by chance, blocks that were close in the original chromosome could still be together in the shuffled chromosome. Methods can detect dependencies between such blocks as their SNVs are correlated (e.g. LD exists). Therefore, we require blocks that were close in the original chromosome to be as far apart in the shuffled chromosome as possible. Similarly, we require that blocks that are close in the shuffled chromosome were far apart in the original chromosome. To achieve this, we enumerated the blocks repeatedly from 1 to c, where we will show in the following how to optimize the number of groups c (see top panel in Fig. S1). Next, the s blocks with the same number are grouped, for example, in Fig. S1, the 4 blocks that are enumerated by 1 are put next to each other. Blocks that were adjacent in the original chromosome are separated by s (group size) blocks in the shuffled chromosome (see Fig. S1). Blocks that are adjacent in the shuffled chromosome were separated by c (number of groups) blocks in the original chromosome. Thus, we have to simultaneously maximize the number of groups c and the group size s, therefore, we set $s = c$. The number of blocks b is chromosome length divided by block length while $b = cs = c^2$. Hence, the optimal number of clusters is the squared root of chromosome length divided by block length ($c = \sqrt{b}$):

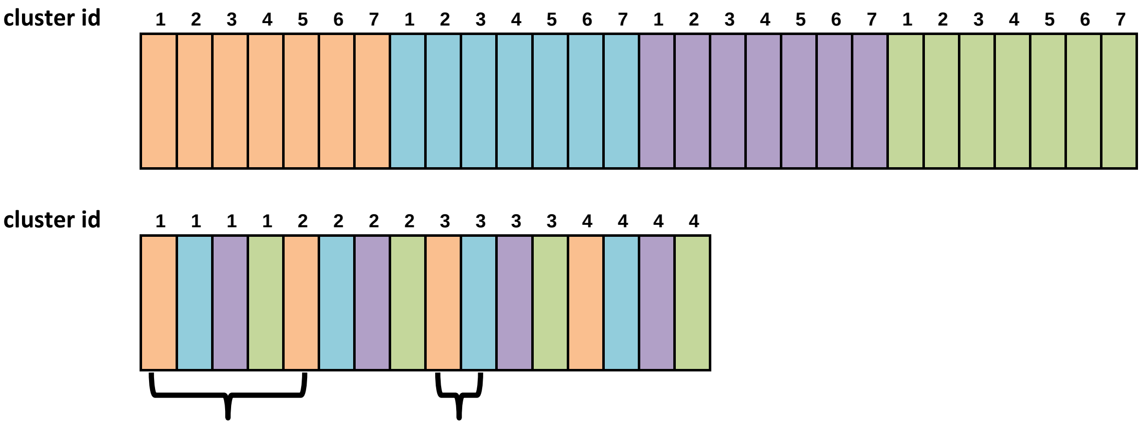

Figure S1: Shuffling of blocks. *Upper panel:* Chromosome is divided into blocks. Then the blocks are enumerated from 1 to c (here 7). *Lower panel:* Blocks with the same number are grouped. Blocks that were close in the original chromosome should not be close in the shuffled chromosome. First bracket: Blocks 1 and 2 of the same color in the upper panel were adjacent in the original chromosome and, therefore, should be well separated in the shuffled chromosome. They are separated by as many blocks as belong to one group minus one (in this example the number of 1's is 4). Second bracket: Blocks of different color labeled "3" are adjacent in the shuffled chromosome and, therefore, should have been separated well in the original chromosome. They are separated in the original chromosome by the number of groups c, marked at the top panel by the number of blocks with the same color (in this example there are 7 groups).

$\sqrt{249,000,000/5,000} = 223$. Consequently, blocks that were adjacent in the original chromosome are now separated in the shuffled chromosome by 1.1 cM ($223 * 5$ kbp= 1.1 Mbp) and vice versa. However, original IBD segments longer than 1 cM may still introduce segments longer than 5 kbp in the shuffled chromosome which will be considered as false positives in this analysis.

In previous simulations like Browning and Browning (11) and Su et al. (12), segments were copied from one individual onto another individual. However, this procedure is not applicable in our setting, for two reasons:

1. The individual from which the segment is extracted may share short IBD segments with other individuals within one 5 kbp block. The number of minor alleles that tag the short IBD segment may even be large. Methods that consider more than two individuals will either miss the implanted segment or detect too many individuals. In the first case, methods may prune the implanted segment off all SNVs outside the short IBD segment. In the second case, methods consider mismatches outside the short IBD segment as sequencing errors or broken segments and consider all individuals that possess the short IBD segment as having the long segment. The problem is that the residual structure within the 5 kbp blocks may be stronger than the structure generated by implanting segments. This problem does not exist if only two individuals are compared to each other but arises if all individuals are compared simultaneously.

2. In most short segments, the majority of observed minor alleles are minor alleles of common SNVs. Therefore, such a segment is tagged by common variants in contrast to our assumptions in this manuscript (see Introduction of the main manuscript).

To implant IBD segments of 10 kbp and 20 kbp length into the shuffled chromosome, we first randomly extract them from the original chromosome. We copy the SNVs within the extracted segment onto SNVs of the shuffled chromosome. Most common variants of the extracted segment are copied onto rare variants of the shuffled chromosome. In the shuffled chromosome most SNVs are rare, however most observed minor alleles stem from common variants. For example, a common SNV with 30% MAF contributes 300 minor alleles to the data set, which requires 300 private SNVs or 30 SNVs with 1% MAF for the same contribution of minor alleles. Copying the nucleotides of the selected segment would result in many adjacent SNVs with a minor allele (top red rectangle in Fig. S2). It may be hard to detect IBD, if only two individuals that possess the segment are considered. However, IBD is detected easily, if multiple individuals are considered, including those that do not possess the IBD segment. The IBD segment clearly sticks out. Therefore, in order to get more realistic data, we only copy the pattern of minor alleles onto the shuffled chromosomes. At locations where the extracted segment has minor alleles, also the implanted segment has its minor alleles (see lower case characters in in the lower rectangle in Fig. S2). To make the break points of the implanted IBD segment recognizable, it has a minor allele at its first and last SNV. Further, a segment is implanted at a region where the SNVs onto which the segment SNVs are copied span about the same genomic region as the extracted segment. This selection of a sufficiently large (in number of bases) region avoids that the SNVs of a 10 kbp or 20 kbp segment are copied onto a 5 kbp or smaller region where the SNVs are denser.

S3.3 Forward Simulation Genotype Data with Implanted IBD Segments

The third data set was constructed by implanting IBD segments into genotype data that has been generated by forward-time simulations. The data is phased per construction, as the forward-time simulation provides the chromosomes for each diploid individual. We now also compare the IBD detection methods on long and medium sized IBD segments (0.5, 1, and 2 Mbp).

In contrast to previous data sets, forward and coalescent simulation ensures linkage disequilibrium on a larger scale and evolutionary relationships between chromosomes. We investigated the coalescent simulation programs MSMS (13), GENOME (14), and COSI (15). We found out that coalescent simulations based on these software packages did not match the characteristics of sequencing data as well as forward-time simulations. In particular the percentage of private / rare SNVs and the distance between SNVs of forward-time simulations were closer to data from sequencing projects (1000 Genomes Project, UK10K, Korean Personal Genome Project) than coalescent simulations. Forward-time simulators are slower than coalescent simulators, but more powerful (16, 17). The forward-time simulator SFS_CODE (18) produced results which were closest to the characteristics of sequencing projects.

With the forward-time simulator SFS_CODE, we generated 147[1] DNA chunks of 300 kbp length each. We used the SFS_CODE commandline

```
sfs_code 1 1 -n 5000 -N 2000 -L 1 300000 -I -r 0.001 \
  -W 2 0 0 0 0.184 0.00040244 -Td 0 1.98 -Td 0.264196 0.12859 \
  -Tg 0.340566 44.75089  -Td 0.340566 0.554513 -Tg 0.39085 282.7192 \
  -TE 0.404845 -o out.txt -e err.txt -F freq.txt
```

[1]We started 150 processes, but 3 processes terminated prematurely.

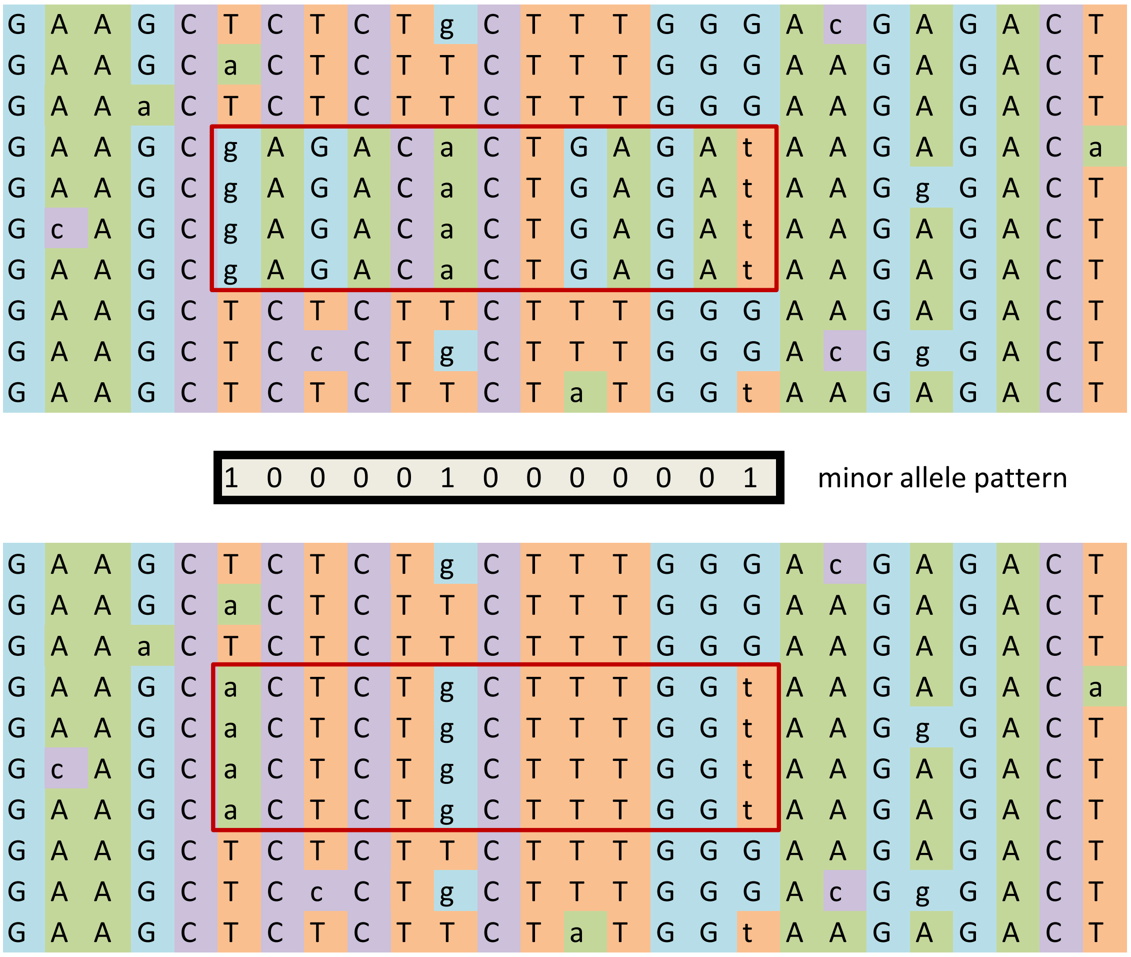

Figure S2: Implanted segments with minor alleles indicated by lower case characters. Rows correspond to individuals and columns to adjacent SNVs. *Upper panel:* Original bases of the segment are implanted. This segment is too easily recognizable because adjacent minor alleles are generated, e.g. the A at position 2 of the segment, which was originally a major allele, would introduce a new minor allele at this position. *Middle panel:* Pattern of minor alleles. *Lower panel:* Only the pattern of minor alleles is implanted. The implanted segment is still similar to the segment of other individuals but tagged/marked by the minor alleles.

which has been taken from Maher et al. (19). Maher et al. modeled a demographic history starting 177 thousand years ago (kya). The model includes an ancient African expansion ($\sim$177 kya, `-Td 0 1.98`), an out-of-Africa bottleneck ($\sim$62 kya, `-Td 0.264196 0.12859`), a founding of Europe bottleneck ($\sim$28 kya, `-Tg 0.340566 44.75089`), an initial phase of exponential growth within Europe ($\sim$28 kya, `-Td 0.340566 0.554513`), and a recent explosive growth phase (starting $\sim$5 kya, `-Tg 0.39085 282.7192`). A DNA chunk length of 300,000 bp (`-L 1 300000`) was generated using a founder population of 2,000 individuals (`-N 2000`), deleterious mutations with a selection coefficient drawn from the Gamma distribution $\Gamma(0.184; 0.00040244)$, a pseudo-infinitely many sites model (`-I`), a burn in phase of 20,000 generations (default $5 \times 2 \times N$), a default population scaled mutation rate of $\theta = 0.001$ per site, and a population scaled recombination rate of $r = 0.001$ per site. 5,000 individuals (`-n 5000`) are sampled from a final population

size of about 140,000.

Joining the 147 DNA chunks led to a DNA strand with 44,094,874 bases. When joining two segments, on average, 35 bases are lost to keep the relative DNA distance of SNVs to their predecessors. We obtained 1,148,822 SNVs for 5,000 randomly sampled individuals. The average number of SNVs in 1,000 individuals is 418,000 with an average distance of 105 bp. This is close to the numbers of the 1000 Genomes Project, where 1,092 individuals have an average SNV distance of 78 bp.

To generate data for an experiment, we first sample 1,000 individuals from the 5,000 individuals. Next we selected an interval containing 10,000 SNVs ($\sim$1 Mbp length) for short IBD segments (10 and 20 kbp) and an interval containing 200,000 SNVs ($\sim$20 Mbp length) for long and medium sized IBD segments (0.5, 1, and 2 Mbp). Then we implant IBD segments into the selected interval, where the IBD segments are taken from individuals that do not belong to the sampled 1,000 individuals. Implantation was performed analogously to the previous experiments that were based on shuffled chromosome 1 data from the 1000 Genomes Project. For implantation, we selected IBD segments that had at least 8 tagSNVs. An IBD segment of length 1 Mbp resulted in 140–250 tagSNVs and a length of 8,000–10,000 SNVs. Table S1 shows an overview over the data sets which are characterized by the length of implanted IBD segments, number of different IBD segments implanted, and how many chromosomes possess a particular IBD segment.

Table S1: Overview of data sets based on forward-time simulation.

	L	F	#I
simA	20	10	1
simB	10	10	1
simC	20	6	1
simD	10	10	20
simE	20	10	5
simAlong	1000	6	1
simBlong	1000	2	1
simClong	2000	6	1
simDlong	2000	2	1
simElong	500	6	1
simFlong	500	2	1

"L" gives the physical length of the IBD segments in kbp, "F" shows how many chromosomes contain the IBD segment, "#I" reports the number of different IBD segments that were implanted.

S4 How Many Individuals Share an IBD Segment?

How often does one find IBD segments that are shared by more than two individuals? The probability of finding IBD segments increases with decreasing segment size (20). Thus, the smaller the IBD segments, the greater the chance of finding multiple individuals who share this segment. In a growing population with N founding haplotypes, the probability that two randomly chosen haplotypes are IBD at a very short random segment (no recombination) is $1/N$ (21). The probability

of k individuals sharing a very small segment is $1/N^{k-1}$. In a cohort of M individuals, there exist $\binom{M}{k}$ sets of k individuals resulting in an expected number of

$$\frac{1}{N^{k-1}} \binom{M}{k} = \frac{M}{k!} \frac{(M-1)}{N} \cdots \frac{(M-k+1)}{N} \tag{S16}$$

segments that are shared by k individuals. This number holds for one DNA location. Genetic drift may have led to more frequent segments than $1/N$ increasing the likelihood of finding IBD segments that are shared by multiple individuals. In summary, many IBD segments will be shared by more than two individuals if (a) the segments are small and (b) the cohort is large.

S5 Small Likelihood of Identity by State: Small MAF vs. Many Individuals

In step 1 of IBD segment extraction from FABIA models as described in subsection "Extraction of IBD Segments from FABIA Models" of the main manuscript, the likelihood of observing identity by state (IBS) without IBD and, therefore, without recombination, is computed. If q is the minor allele frequency (MAF) for one SNV, the probability of observing the minor allele of this SNV in all t individuals is q^t. We assumed that all SNVs have the same MAF q — in the experiments we used the average MAF. According to the main manuscript, the probability of observing k or more counts of model SNVs by chance in an interval of n SNVs is

$$\binom{l}{t} \sum_{i=k}^{n} \binom{n}{i} q^{it} \left(1 - q^t\right)^{n-i} , \tag{S17}$$

where l is the number of individuals and $\binom{l}{t}$ is the number of possibilities to chose t individuals from the l individuals of the study. The number of counts i runs from k to the number of SNVs n.

If we try to minimize this probability, we observe a trade-off between small average minor allele frequency (MAF) q and the number t of individuals that share an IBD segment. For a small IBS likelihood, the average MAF q should be small and t large. However, increasing t also increases the lower bound $\frac{t}{l}$ on q. If we assume minimal $q = \frac{t}{l}$, then above probability Eq. (S17) is governed by the term

$$\left(\frac{t}{l}\right)^{it} \left(1 - \left(\frac{t}{l}\right)^{t}\right)^{n-i} . \tag{S18}$$

For small $q = \frac{t}{l}$ we have $\left(1 - \left(\frac{t}{l}\right)^t\right)^{n-i} \approx 1$. The function $\left(\frac{t}{l}\right)^{it}$ has its minimum at $t = l/e$ independent of i (e is Euler's number). Thus, if the factor $\binom{l}{t}$ is ignored and $t = l/e$ individuals share the minor allele, the evidence for IBD is maximal.

To avoid IBS without IBD by recombination, we focused on rare SNVs (see main manuscript). IBD can be distinguished from IBS without IBD by rare alleles, because for them two independent origins are unlikely, so IBS generally implies IBD, which is not true for common alleles (22, chapter 15.3, p. 441). Hence, the minimum $t = l/e$ will not be attained with rare SNVs. For fewer individuals t than the minimum $t = l/e$, the function $\left(\frac{t}{l}\right)^{it}$ is decreasing. This justifies that more individuals give more evidence for IBD because random minor allele sharing (IBS without IBD) is less likely.

S6 IBD Segments based on Likelihood and Dosage

We compare the IBD segments that HapFABIA detects if it uses genotype, haplotype, dosage, or likelihood data from the 1000 Genomes Project (chromosome 1). For the likelihood, HapFABIA uses one minus the likelihood of observing only the major allele. This value is thresholded by 0.6, i.e. only if the likelihood of observing only the major allele is below 0.4, a value different from zero is provided. For dosage, HapFABIA uses the raw value.

First we present a typical example from the 1000 Genomes Project using data from chromosome 1. For the interval from SNV number 1,000,000 to 1,010,000, 205 IBD segments were detected in genotype data, 206 in haplotype data, 207 in dosage data, and 210 in likelihood data. The number of IBD segments that match between two types of data is 143 for G-H, 155 for G-D, 144 for G-L, 137 for H-D, 136 for H-L, and 136 or D-L (G=genotype, H=haplotype, D=dosage, L=likelihood). For deciding whether two IBD segments match or not, we used hierarchical clustering according to the pseudo-distance measure described in the main manuscript in section "Extraction of IBD Segments from FABIA Models". A match of two original IBD segments is indicated by merging them in hierarchical clustering. The number of IBD segments after hierarchical clustering is 218 for G-H, 205 for G-D, 226 for G-L, 222 for H-D, 235 for H-L, and 238 for D-L. For example, 75% of IBD segments extracted from genotype data can be matched with IBD segments from dosage data and vice versa. HapFABIA's step 4 in the second stage ensures that IBD segments within genotype data or within dosage data are not similar to each other, therefore, during hierarchical clustering only IBD segments from genotype data can be merged with IBD segments from dosage data.

Table S2: Average percentages of IBD segments that match between genotype (G), haplotype (H), dosage (D), and likelihood (L).

	G	H	D	L
G	–	64	74	61
H	71	–	70	62
D	74	64	–	61
L	71	64	71	–

Percentages are given with respect to the row data. For example, 71% of IBD segments detected in haplotype data are also found in genotype data, but only 64% of IBD segments detected in genotype data are also found in haplotype data. Results are based on genotyping data from chromosome 1 of the 1000 Genomes Project.

We computed the average matching of IBD segments extracted from different kind of data across all intervals of chromosome 1 based on the data from the 1000 Genomes Project. Table S2 reports the average percentage of IBD segments that could be matched between data types. The agreement of the detected IBD segments between detections based on different genotype values is high considering the inconsistencies between the different genotype measurements. In the following we describe some of these inconsistencies.

The difference between genotype and haplotype based IBD segment detection is often caused by phasing errors (examples of such phasing errors can be found at `http://www.bioinf.jku.`

at/research/short-IBD). Genotype and dosage agree at about 74% of their IBD segments. However, many inconsistencies between dosage and genotype exist. For example, the record [0|0:1.000:-0.48,-0.48,-0.48] is observed very often, which means that, for dosage, the minor allele is one, while it is zero in the genotype. Each record describes an individual-SNV pair. The first entry is the phased first haplotype, where 1 means that the minor allele is present. To the right of "|", the minor allele of the second haplotype is given. Next, after ":", the dosage is reported and finally the $\log_{10}$ likelihood of the genotypes 0|0, 1|0 or 0|1, and 1|1. Records like [0|0:1.000:-1.16,-0.38,-0.29] are usual as well, where the dosage value contradicts the called genotype. The same dosage may have different genotype values like [0|0:0.550: -0.48,-0.48,-0.48] vs. [0|1:0.550:-0.28,-0.43,-0.96].

The likelihood values show the largest deviation from the genotypes. The likelihood is often missing like in the record [1|1:1.400:0,0,0] or in the record [1|0:1.200:0,0,0]. In many cases the likelihood is equally likely for each genotype, e.g. for [0|0:0.000:-0.48, -0.48,-0.48] or [1|0:1.000:-0.48,-0.48,-0.48]. Disagreements between likelihood and genotype/dosage can often be seen like [1|0:0.950:-0.10,-0.68,-5.00] or [1|0:0.950: -0.10,-0.67,-3.38], where minor alleles have low likelihood but are called. Furthermore, similar likelihood values may have different genotype/dosage values like [0|0:0.000:-0.19, -0.45,-2.24] vs. [0|1:0.900:-0.19,-0.46,-2.51].

In summary the agreement of IBD segments based on genotype, haplotype, dosage, and likelihood is high given the inconsistencies in the data.

S7 Identification of IBD Segment tagSNVs by Linkage Disequilibrium

We investigated to what extent linkage disequilibrium (LD) can help to find IBD segments or to identify haplotype blocks with a focus on rare variants. For rare variants, LD has high variance (23, 24). Therefore, it is difficult to employ LD for identifying IBD segments or haplotype blocks that contain mainly rare variants.

To see how well LD detects tagSNVs of IBD segments, we constructed a classification task using the artificial data sets. SNV pairs are classified as belonging — or not belonging — to an IBD segments. Pairs of IBD segment tagSNVs are true pairs, and others are false pairs. SNV pairs are ranked according to their LD values and the top-ranked pairs are considered to be positive SNV pairs. We are interested in whether IBD segments can be detected using the LD values, rather than evaluating this classification task. Therefore, we counted how many IBD segment tagSNVs are identified via SNV pairs with high LD values — the number of true positives (TP) — and how many non-IBD segment SNVs are falsely detected — the number of false positives (FP). If an IBD segment tagSNV is detected once via a true SNV pair, it counts as a true positive, even if it participates in false pairs. This evaluation takes two issues into account: First, an IBD segment tagSNV needs to be detected only once. Secondly, spurious similarities are penalized as false positives because they mislead a subsequent IBD segment extraction procedure. The power (true positive rate) and the false discovery rate (FDR) can be computed using the IBD segment tagSNVs (true SNVs) and the SNVs belonging to a top-ranked pair (positive SNVs). For each SNV pair, PLINK computes the correlation r^2, which measures the LD independently of allele frequencies. Values for r^2 are only computed within a window of 500 SNVs to consider local agglomerations

of IBD segment tagSNVs — the same window size is used for HapFABIA in the experiments with the artificial data sets. Pairs are first ranked according to r^2 and then the top-ranked 100, 1,000, or 10,000 pairs are selected. Table S3 summarizes 100 runs for each data set and reports the median values for the number of true positives (TP), the number of false positives (FP), the power (recall or the true positive rate), and the false discovery rate (FDR). The FDR is extremely high, with a median of 0.99 (mean 0.99) and a minimal average value of 0.964 for data set artC.

Table S3: Identification of IBD segment tagSNVs based on linkage disequilibrium.

#pairs	100	1,000	10,000	100	1,000	10,000
	artA100			artA		
TP	3	15	36	2	14	42
FP	186	1634	7988	195	1792	8555
power	0.015	0.075	0.18	0.01	0.07	0.21
FDR	0.984	0.991	0.996	0.990	0.992	0.995
	artB100			artB		
TP	4	12	32	5	14	39
FP	181	1628	8038	9608	1784	8560
power	0.02	0.06	0.162	0.025	0.07	0.195
FDR	0.978	0.993	0.996	0.975	0.992	0.995
	artC100			artC		
TP	4	11	33	6	16	40
FP	9659	1332	7921	173	1674	8500
power	0.02	0.055	0.165	0.03	0.08	0.2
FDR	0.970	0.991	0.996	0.964	0.990	0.995
	artD100			artD		
TP	2	6	13	4	9	16
FP	147	1073	7721	167	1543	8414
power	0.02	0.06	0.13	0.04	0.09	0.16
FDR	0.987	0.994	0.998	0.977	0.994	0.998

An SNV is a positive IBD segment tagSNV if it belongs to at least one top-ranking (according to its LD value) SNV pair. Values are given for top-ranking 100, 1,000, and 10,000 SNVs. We report true positives (TP), false positives (FP), power (recall or true positive rate), and false discovery rate (FDR). Each value corresponds to the median of 100 runs for each of the data sets artA100, artA, artB100, artB, artC100, artC, artD100, and artD. The FDR is strikingly high.

If more IBD segments with the same size exist at the same DNA location, even if they contain only common variants, then haplotype block identification may be still possible. However, in the 1000 Genomes Project data we found many IBD segments which contained only few common variants.

S8 Calls, Filters, and Parameter Settings for the Methods

S8.1 Commandline Calls

We used the following commandline calls for the methods that were compared. The calling parameters are different for the different data sets and are described in the next subsection.

- HapFABIA:
  Called in the R software package by

```
R> result <- hapFabia(fileName,p=10,iter=1,alpha=0.03,cyc=50,
+  IBDsegmentLength=50,Lt = 0.1,Zt = 0.2,thresCount=1e-5,
+  mintagSNVsFactor=3/4,pMAF=0.035,haplotypes=TRUE,avSNVsDist=100)
```

- fastIBD:

```
java -Djava.io.tmpdir=/tmp -jar beagle.jar unphased=databeagle \
  fastibd=true missing=N out=beagleres.txt
```

- PLINK:

```
plink --file dataplink --indep 50 5 2 --out dataplink
plink --file dataplink --extract dataplink.prune.in --make-bed \
  --out dataplinkpruned
plink --bfile dataplinkpruned --noweb --genome --allow-no-sex \
  --out plink
plink --bfile dataplinkpruned --noweb --read-genome \
  plink.genome --segment --all-pairs --segment-length 5 \
  --segment-snp 50 --out plink
```

- GERMLINE:
  Content of the file germline_input is

```
1
dataplink.map
dataplink.ped
germline_output
```

  The commandline call is

```
germline -haploid -min_m 0.001 -err_hom 0 -err_het 0 \
  -bits 30 < germline_input
```

- DASH:
  The results of GERMLINE, which are stored in the file germline_output.txt.match, are filtered and then written into the file germline_result_filtered. Then DASH is called by

```
cut -f 1,2,4 germline_result_filtered | dash_cc dataplink.fam \
  dash_output -min 2
```

- MCMC:

```
MCMC_IBDfinder -g datamcmc.genotype -p datamcmc.posmaf -z datamcmc.initz
```

- Relate:
  The content of the parameter file `relate_options` is

```
1     #1=allpairs 0=normal run
8     #pair[0]
9     #pair[1]
0     #double recombination
0     #LD=0=rsq2 LD=1=D
0.001 #min default: 0.025
0.001 # alim[0]
5.0   # alim[1]
0     #doParameter calculation (pars)
0.3   # par[0] = a this is only used if doParameter is set to 1
0.25  # par[1] = k2 this is only used if doParameter is set to 1
0.5   # par[2] = k1 this is only used if doParameter is set to 1
1     #ld_adj
0.01  #epsilon
5     #back default 50
0     #doPrune
0.1   #prune_value
0     #fixA
0.0   #fixA_value
1     #fixK2
0.0   #fixk2_value
1     #calculateA
0.013 #phi_value
0.1   #convergence_tolerance
3     #times_to_converge
8     #times_to_run
100   #back2
```

  The commandline call is

```
relateHMM -o relate_options -g datarelate.geno  -c datarelate.chr \
  -p datarelate.pos -post relate_post.txt -k relate_stat.txt
```

- fastPHASE

```
fastPHASE -Pzp -T1 datafastPHASE.txt
```

or with the `-K` option

```
fastPHASE -Pzp -T1 -K400 datafastPHASE.txt
```

S8.2 Arguments and Filters for the Different Data Sets

Except for HapFABIA, we optimized the calling arguments of the methods and filters on the results on 5 additional data sets for each of the experiments. This optimization should ensure that the parameters and filters are close to those the authors of the methods would use. We aim at removing biases that result from being more familiar with one method and therefore being able to choose a more appropriate parameter setting for an experiment. The parameters of HapFABIA are not optimized but chosen according to characteristics of the data set (e.g. average minor allele frequency) and the lengths of IBD segments that should be extracted.

We report the arguments used at the calls of the different methods in the tables below. The term "artificial" denotes data sets with artificially generated chromosomes into which short artificial IBD segments are implanted: artA100, artA, artAMis, artB100, artB, artBMis, artC100, artC, artCMis, artD100, artD, and artDMis (see main manuscript for details on these data sets). Artificial data sets with "no mismatches" are artA100, artA, artB100, artB, artC100, artC, artD100, artD, while artificial data sets with "mismatches" (up to 6 mismatches) are artAMis, artBMis, artCMis, and artDMis. The term "implanted" denotes data sets based on shuffled 5 kbp blocks of the 1000 Genomes Project into which short real IBD segments are implanted: impA, impB, impC, impD, and impE. The term "simulated" denotes data sets based on forward-time simulation into which short IBD segments (also from the simulation) are implanted: simA, simB, simC, simD, and simE. The term "simulated long" denotes data sets based on forward-time simulation into which short and medium sized IBD segments (also from the simulation) are implanted: simAlong, simBlong, simClong, simDlong, and simElong. As simElong and simFlong contain implanted IBD segments of 0.5 Mbp, filter criteria are adjusted to this length.

fastPHASE was used with option `-T1` to allow only one random start and either without fixing K or with `-K400`. We used this value of K because up to 400 IBD segments were found in a 10,000 SNV interval in the 1000 Genomes Project data.

For BEAGLE/fastIBD, we used for short IBD segment thresholds of 1e-10 (fastIBD-1) and 1e-13 (fastIBD-2). Smaller values lead to power close to 0. If these thresholds do not yield a detection, they are increased by a factor of 10 until IBD segments are detected. Using the filter "length", detected IBD segments are enforced to have a minimal length in kbp. Table S4 gives an overview over the fastIBD parameters and filters used for the different data sets.

For PLINK, we first pruned SNVs using the option `--indep 50 5 2`. LD-based pruning was recommended by the authors of PLINK and was performed in most previous comparisons of IBD methods. We adjusted the minimal segment length (`--segment-length`) and the minimal number of SNVs in segment (`--segment-snp`). For `segment-length`, smaller values than 0.1 did not change the result if compared to the value 0.1. Using the filter "length", detected IBD segments are enforced to have a minimal length in kbp. Table S5 gives an overview over the PLINK parameters and filters used for the different data sets.

Table S4: Beagle/fastIBD filters on probability and minimal IBD length.

Data set	filter 1	filter 2	length
artificial	1.0e-10	1.0e-13	—–
implanted	1.0e-10	1.0e-13	> 5 kbp
simulated	1.0e-10	1.0e-13	—–
simulated long			
simAlong to simDlong	1.0e-50	1.0e-80	—–
simElong and simFlong	1.0e-40	1.0e-60	—–

Table S5: PLINK arguments on minimal segment length and minimal number of SNVs in segment and filter on minimal IBD length.

Data set	segment-length	segment-snp	length
artificial	0.1	20	—–
implanted	0.1	20	> 5 kbp
simulated	5	50	—–
simulated long			
simAlong to simDlong	600	6000	—–
simElong and simFlong	300	3000	—–

Simulated data sets had more private SNVs than the artificial data sets, therefore, segment-length and segment-snp had different optimal values.

For GERMLINE, we adjusted the minimum length for matches (`min_m`), the maximum number of mismatches of homozygous and heterozygous SNVs (`-err_hom` and `-err_het`), and the number of SNVs (`-bits`) in an initial (seed) segment. To avoid an excessive number of false positives, we filtered GERMLINE results to keep only longer segments. For example, with the artificial data, only IBD segments with more than 150 (GERMLINE-1) or more than 200 (GERMLINE-2) SNVs are kept — these values match the sizes of the implanted IBD segments. For implanted IBD segments in the shuffled chromosome 1 of the 1000 Genomes Project, we used `-bits 128`, and `-err_hom 0` as well as `-err_het 0` (we did not simulate sequencing errors). For this data set, GERMLINE extracted 30 million IBD regions and had an extremely high FDR with `-bits 30`. Therefore, we used for this data set stricter filtering criteria for GERMLINE. We kept segments greater or equal to 260 SNVs (GERMLINE-1) or greater or equal to 380 SNVs (GERMLINE-2) in order to reduce the FDR — these lengths were found to be optimal for this experiment.

For simulated data sets simB and simC with 10 kbp long implanted IBD segments , we called GERMLINE with `-bits 80`, which gives an average physical length of the seed of 8 kbp as the average distance between SNVs is 105 bases. To reduce the FDR we kept segments greater or equal to 70 SNVs (GERMLINE-1) or greater or equal to 90 SNVs (GERMLINE-2). For simulated

data sets simA, simC, and simE with 20 kbp long implanted IBD segments , we called GERMLINE with `-bits 170`, which gives an average physical length of the seed of 17 kbp. To reduce the FDR we kept segments greater or equal to 150 SNVs (GERMLINE-1) or greater or equal to 180 SNVs (GERMLINE-2). Though the filter of GERMLINE-2 is only slightly longer than the seed, the power dropped drastically in the experiments. Using the filter "length", detected IBD segments are enforced to have a minimal length in kbp. Table S6 gives an overview over the GERMLINE parameters and filters used for the different data sets.

Table S6: GERMLINE parameters and filters.

Data set	min_m	err_hom	err_het	bits	filter 1	filter 2	length
artificial							
no mismatches	0.001	0	0	30	150	200	—
mismatches	0.001	6	6	30	150	200	—
implanted	0.001	0	0	128	260	380	> 5 kbp
simulated							
simA, simC, simE	0.001	0	0	170	150	180	—
simB, simD	0.001	0	0	80	70	90	—
simulated long							
simAlong to simDlong	0.5	0	0	1000	7000	5000	—
simElong and simFlong	0.3	0	0	700	4000	3000	—

Minimum length for match (min_m), maximum mismatches of homozygous and heterozygous SNVs (`-err_hom` and `-err_het`), number of SNVs in IBD segment for seed (`-bits`), filter on the length of found segments in number of SNVs (filter 1 and filter 2), filter on minimal IBD segment length in kbp (length). For implanted data sets `-bits 30` resulted in more than 100 million detected segments which lead to an FDR of 1, therefore we set `-bits 128`. For the simulated data sets we used `-bits 80` for IBD segment length 10 kbp and `-bits 170` for 20 kbp (the average distance between SNVs is 105 bases). If we used more stringent filters for the simulated data like `-bits 128` or 150 for the filter on simB or simD, then we had power of zero (all 10 kbp segments were filtered out).

DASH is based on the output of filtered GERMLINE-1 results and filtered for a minimal number of individuals in a cluster (`-min`). Using the filter "length", detected IBD segments are enforced to have a minimal length in kbp. Table S7 gives an overview over the DASH parameters and filters used for the different data sets.

The default FABIA parameters are `p=10` (10 biclusters), `alpha=0.03` (default sparseness value), `cyc=50` (number of cycles of the EM algorithm), `iter=1` (number of iterations). For IBD segment extraction the default settings are `IBDsegmentLength=50` kbp (typical length of IBD segments), `thresCount=1e-5` (p-value for tagSNV counts in a bin to detect IBD segments and to distinguish IBS from IBD), `mintagSNVsFactor=3/4` (minimal IBD segment overlap between two chromosomes to call an IBD segment). Furthermore, `Lt=0.1` (percentage of top bicluster SNVs considered) and `Zt=0.2` (percentage of top bicluster samples considered). Variable pMAF is the estimated average minor allele frequency. Further filters are minimal number of individuals ("min") possessing an IBD segment and minimal physical length in kbp ("length") of IBD seg-

Table S7: Dash parameters and filters.

Data set	min	length
artificial	2	—
implanted	5	> 5 kbp
simulated	2	—
simulated long	2	—

`-min` enforces a minimal number of individuals in each cluster and “length” gives the minimal physical length of IBD segments in kbp.

ments. For the 1000 Genomes Project data, we performed more iterations. The value `pMAF=0.035` was estimated on data from the 1000 Genomes Project by counting how many of $n = 500$ SNV bins contain 10 or more minor alleles in an individual. We know the true `pMAF=0.04` only for the artificial data. Table S8 gives an overview of the HapFABIA parameters and filters used for the different data sets.

Table S8: HapFABIA parameters and filters.

Data set	iter	IBDsegmentLength	pMAF	min	length
artificial					
artA–artCMis	1	50	0.04	2	—
artD, artD100, artDMis	2	50	0.04	2	—
implanted					
impA–impC, impE	1	50	0.035	5	> 5 kbp
impD	2	50	0.035	5	> 5 kbp
simulated long					
simA–simC, simE	1	50	0.035	2	—
simD	2	50	0.035	2	—
simulated long					
simAlong–simDlong	1	5000	0.035	2	—
simElong, simFlong	1	1000	0.035	2	—
1000 Genomes	40	50	0.035	2	—

`iter` is the number of iterations of FABIA, where 10 biclusters are extracted in each iteration. `IBDsegmentLength` is the typical length of IBD segments (in kbp) for extraction and simultaneously determines the tagSNVs accumulation threshold. This parameter is based on the assumption of an average distance of 100 bases between SNVs: `avSNVsDist=100`. `pMAF` is the average minor allele frequency for determining the tagSNVs accumulation threshold. "min" gives the minimal number of individuals that must possess the IBD segment (see DASH). For the artificial data, `pMAF=0.04` was known. For data sets "D", we implanted more IBD segments, therefore increased `iter` to 2. More iterations than the chosen `iter` value did not change the results because no more biclusters are found. For the 1000 Genomes project, in most cases smaller `iter` values, like `iter=20`, would be sufficient as no more biclusters are found in later iterations.

S9 Population Groups of the 1000 Genomes Project

The subpopulations of the 1000 Genomes Project are given in Tab. S9 and the locations, where the individuals reside, are shown on the map in Fig. S3.

Table S9: Overview of population groups of the 1000 Genomes Project.

Pop	#Ind	Grp	Location
ASW	122	AFR	Americans with African ancestry from SW US
YRI	176	AFR	Yoruba in Ibadan, Nigeria
LWK	194	AFR	Luhya from Webuye, Kenya
CLM	120	AMR	Colombians in Medellin, Colombia
MXL	132	AMR	Mexicans from Los Angeles, California
PUR	110	AMR	Puerto Ricans
CEU	170	EUR	Utah residents with ancestry from northern and western Europe
FIN	186	EUR	Finnish
GBR	178	EUR	British from England and Scotland
IBS	28	EUR	Iberians from Spain
TSI	196	EUR	Toscani in Italy
CHB	194	ASN	Han Chinese from Beijing
CHS	200	ASN	Han Chinese from South
JPT	178	ASN	Japanese from Tokyo, Japan

The column labeled “Pop” gives the population, “#Ind” reports the number of individuals, “Grp” gives the continental group, and “Location” the location from where the individuals stem.

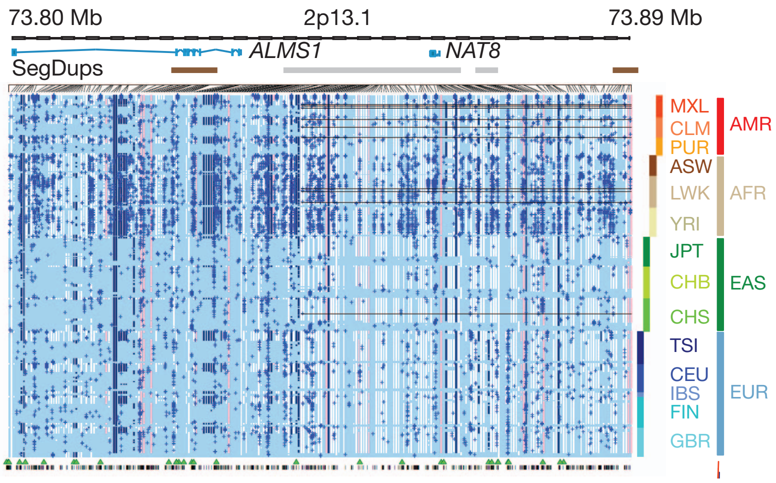

Figure S3: Map indicating the locations of the populations the 1000 Genomes Project phase I. Figure taken from (25).

S10 Calculation of IBD Lengths

The raw lengths were computed as the length of the maximal IBD sharing between two individuals that possess the IBD segment. This resulted in overestimation of the lengths, which are corrected as described below.

Furthermore, we correct IBD segment lengths for IBD with archaic genomes, as archaic genomes only match a part of the IBD segment.

S10.1 Correction for the Assumptions of IBD Length Distributions

The raw IBD segment lengths are computed as the maximal IBD sharing of any two individuals that possess the IBD segment. The exponential distribution of IBD lengths as explained in Subsection S13.1 was derived for pairs of haplotypes (21, 26, 27, 28). Thus, our raw IBD segment lengths are longer than the IBD lengths for pairs of haplotypes. To apply the assumption of exponentially distributed IBD segment lengths requires the correction of the raw IBD segment lengths. We observed a second cause for raw IBD segments being longer than expected by the exponential distribution. The more individuals share an IBD segment, the more likely it is to find two individuals that share random minor alleles which would falsely extend the IBD segment.

Therefore, we correct the raw lengths of IBD segments by locating the first tagSNV from the left (upstream) which is shared by 3/4 of the individuals that possess the IBD segment. This tagSNV is the left break point for the IBD segment. Analogously, we determine the right break point by the first tagSNV from the right (downstream) that is shared by 3/4 of the individuals. The distance between these break points is the (corrected) length of an IBD segment.

S10.2 Length Correction for IBD with Archaic Genomes

We are interested in IBD between human and archaic genomes. However, the human IBD segment length is not an appropriate measure for the length of IBD with archaic genomes because only a part of the IBD segment may match an archaic genome (see Figures S31, S32, and S33).

We correct the IBD segment lengths to obtain the IBD lengths between human and archaic genomes. The corrected length of an IBD segment is the length of the "archaic part" that matches a particular archaic genome. First, the left (upstream) break point of the "archaic part" of an IBD segment genome is detected. This left break point is defined as the first location in the IBD segment from the left (upstream), where at least 4 out of 9 tagSNVs match the archaic genome. From the right (downstream), the right break point of the "archaic part" of an IBD segment was detected analogously. Since not all bases of the Neandertal genome were called, we modified the definition of the break points. For the Neandertal genome, a break point requires at least 5 or 6 bases of the 9 tagSNVs to be called of which 2 or 3, respectively, have to match the Neandertal genome. If either the left or right break point of an "archaic part" could not be found, then this IBD segment does not contain an "archaic part".

Matching of an IBD segment and an archaic genome for IBD segment lengths analyses was defined as:

1. at least 15% of the tagSNVs of the IBD segment must match the archaic genome,

2. 30% of tagSNVs in the “archaic part” of the IBD segment must match the archaic genome, and

3. the “archaic part” of the IBD segment must at least contain 9 tagSNVs.

S11 Are Rare Variants Recent or Old?

Rare variants are found to be old which might seem to contradict results of other researchers. In the following two subsections, we show that our conclusion that rare variants can be old does not contradict previous findings. In the first subsection, we argue that only because rare variants are in general recent does not contradict that some rare variants are old. In the second subsection, we support the viewpoint that rare variants are old by other publications.

S11.1 Some Rare Variants are Recent and Some Old

That rare variants are, in general, recent does not contradict our results. On the contrary, that the majority of IBD segments extracted by HapFABIA is found in Africans can explain why three times as many variants with 0.5–5% minor allele frequency are found in Africans as in European or Asians in the 1000 Genomes Project (25). In particular, if rare variants are caused by a recent population growth, then the numbers found by the 1000 Genomes Project (25) are difficult to interpret. We want to discuss two reasons why rare variant may be old.

(I) Many rare variants are old, however, most rare variants may be recent, e.g. because of a recent growth in population. We did not consider private variants and have a bias toward less rare variants because, the more individuals share an IBD segment, the higher its significance, the more likely it is detected by HapFABIA. In our analysis, only 39% of the rare variants (not counting private variants) are in IBD segments. Therefore, the remaining majority of rare and private variants might be recent.

The vast majority of IBD segments is found in Africans. This would explain why Africans have more rare and low frequent variants than Europeans or Asians. The publication of the 1000 Genomes Project (25) reports:

> “individuals from populations with substantial African ancestry (YRI, LWK and ASW) carry up to three times as many low-frequency variants (0.5–5% frequency) as those of European or East Asian origin,”

Table S14 in the supplementary information of the publication of the 1000 Genomes Project (25) provides the number of derived variants per individual in each population in its last rows (DAF denotes “derived allele frequency”, i.e. the frequency of the mutation):

	African			European				
	ASW	LWK	YRI	CEU	FIN	GBR	IBS	TSI
DAF <0.5%	116,216	152,267	122,978	30,034	32,918	31,944	36,129	33,425
DAF 0.5-5%	558,996	649,303	683,239	143,987	146,737	146,802	155,270	146,066
	Asian			admixed American				
	CHB	CHS	JPT	CLM	MXL	PUR		
DAF <0.5%	36,081	40,974	45,107	45,184	38,654	48,892		
DAF 0.5-5%	119,668	118,137	120,115	189,257	163,737	214,430		

Recent variants are supposed to be derived variants. The table shows about four times more variants with derived allele frequency of 0.5-5% in Africans than in other populations (if we ignore the admixed Americans that have African admixture). For derived allele frequency $< 0.5\%$, we observe 2.5 times more variants in Africans than in Europeans or Asians.

(II) Many rare variants are old, however, compared to common SNPs, they are recent, i.e. the temporal relations remain, but variants are dated further back. We state in the manuscript that many rare variants are old and from times before humans migrated out of Africa. However, many common SNPs are even older and stem from common ancestors of human and chimpanzee. This means that many rare variants are old, but compared to common SNPs they are recent.

That common SNPs are old is supported by findings of the Chimpanzee Consortium. In Hacia et al. (29) it was found that, of 397 human SNP sites, 214 were ancestral (shared with common ancestors of chimpanzee and human). Of the ancestral SNPs, 1/4 had the minor allele as ancestral allele. For the chimpanzee genome (30), it was found that

> "Of $\sim$7.2 million SNPs mapped to the human genome in the current public database, we could assign the alleles as ancestral or derived in 80% of the cases according to which allele agrees with the chimpanzee genome sequence"

and that

> "a significant proportion of derived alleles have high frequencies: 9.1% of derived alleles have frequency $\geq$ 80%."

According to (30, Suppl. Fig. S9) about 25% of the derived alleles have frequency $\geq$ 50% in which case the minor allele is the ancestral allele.

That some SNPs are very old and that some haplotypes are shared between humans and chimpanzee was also found in Leffler et al. (31):

> "We conducted a genome-wide scan for long-lived balancing selection by looking for combinations of SNPs shared between humans and chimpanzees. In addition to the major histocompatibility complex, we identified 125 regions in which the same haplotypes are segregating in the two species,"

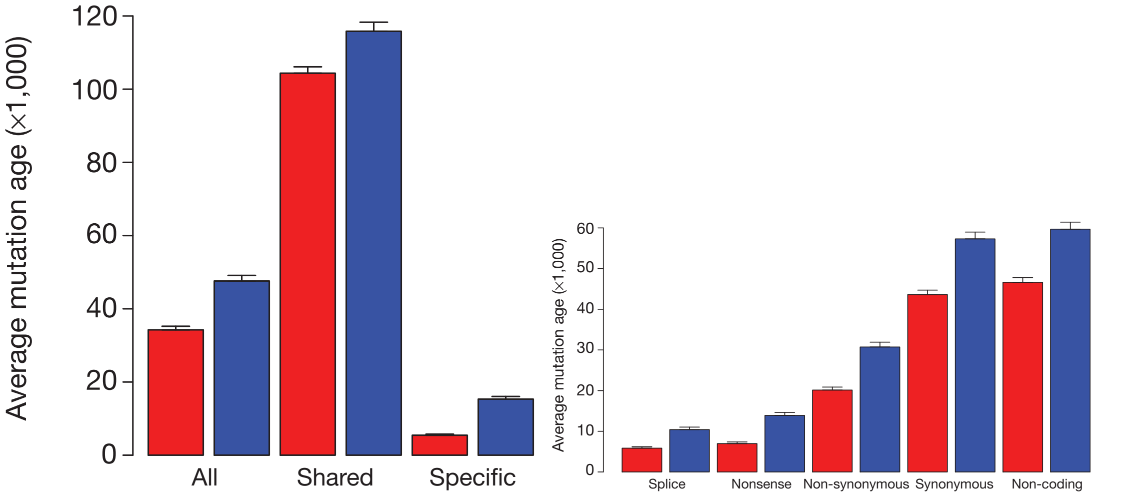

Figure S4: Left: Average age of all SNVs, "Shared" SNVs found both in European Americans (red) and in African Americans (blue), and SNVs found in only one population ("Specific"). Right: Average age for different functional types of variants. Both plots are taken from Fu et al. (32).

S11.2 Old SNVs Have Been Reported in Other Publications

In a recent publication (32), it was found that

> "The average age across all SNVs was 34,200 $\pm$ 900 years ($\pm$ s.d.) in European Americans and 47,600 $\pm$ 1,500 years in African Americans, and these estimates were robust to sequencing errors [...]".

The authors further write

> "SNVs shared between European Americans and African Americans were significantly older (104,400 years and 115,800 years for European Americans and African Americans, respectively)".

These figures are visualized as bar plots in Fig. S4.

S12 Sharing of IBD Segments Between Populations and Genomes

S12.1 Sharing of IBD Segments Between Continental Groups

We found that most IBD segments are observed in only one continental group, which are, in most cases, Africans. However, for other continental groups, we saw a considerable sharing of IBD segments across continental groups. The sharing between continental groups seems to contradict statements in other publications. For example, Gravel et al. (33) report that sharing of SNVs between continental groups is low.

Sharing between continental groups has been reported in the publication of the 1000 Genomes Project (25). In our nomenclature, the terms “low-frequency variants” and “rare variants” from (25) are unified to the term “rare variants”. The 1000 Genomes Project (25) use the term “ancestry groups” to denote EUR, EAS (ASN in our nomenclature), AFR, and AMR according to their Fig. 2b. The authors of the 1000 Genomes Project (25) write:

> “By contrast, 17% of low-frequency variants in the range 0.5–5% were observed in a single ancestry group, and 53% of rare variants at 0.5% were observed in a single population (Fig. 2b).”

This means that 83% of the low-frequency variants are shared between continental groups and 47% (almost half) of the rare variants are shared between continental groups.

Another example taken from the 1000 Genomes Project paper (25) is:

> “Some sharing patterns are surprising; for example, 2.5% of the f_2 FIN–X variants are shared with YRI or LWK populations.”

f_2 variants are those variants for which their minor allele is observed in exactly 2 individuals. f_2 variants are rarer than most SNVs that tag our extracted IBD segments because, on average, 6 individuals possess an IBD segment. According to (33), sharing between continental groups decreases with rarer variants. Thus, in our extracted IBD segments, we would expect to find sharing between continental groups considerably more often than for f_2 variants.

Furthermore, the same publication (25) reports:

> “By contrast, between populations with no recent shared ancestry, f_2 variants are present on very short haplotypes, for example, an average of 11 kb for FIN–YRI f_2 variants (median between ancestry groups excluding admixture is 15 kb), and are therefore likely to reflect recurrent mutations and chance ancient coalescent events.”

This statement also supports our findings of IBD sharing between continental groups, but we could exclude recurrent mutations because IBD segments are shared between multiple individuals.

S12.2 IBD Segments that Match Archaic Genomes

We tested whether IBD segments that match particular archaic genomes to a large extent are found more often in certain populations than expected randomly. For each IBD segment, we compute two

values: The first value is the proportion of tagSNVs that match a particular archaic genome, which we call "genome proportion" of an IBD segment (e.g. "Denisova proportion"). The second value is the proportion of individuals that possess an IBD segment and are from a certain population as opposed to the overall number of individuals that possess this IBD segment. We call this value the "population proportion" of an IBD segment (e.g. "Asian proportion"). Consider the following illustrative examples: If an IBD segment has 20 tagSNVs of which 10 match Denisova bases with their minor allele, then we obtain 10/20=0.5=50% for the Denisova proportion. If an IBD segment is observed in 6 individuals of which 4 are Africans and 2 Europeans, then the African proportion is 4/6=0.67=67% and the European proportion is 0.33=33%.

We define an IBD segment to match a particular archaic genome if the genome proportion is 30% or higher. We plot densities of population proportions for IBD segments that match a particular archaic genome and for those that do not match that genome. Figure S5 shows the density of Asian proportions of Denisova-matching IBD segments (pink) vs. density of Asian proportions of non-Denisova-matching IBD segments (cyan). Figure S6 shows analogous densities for Europeans. Denisova-matching IBD segments are observed more often in Asians and Europeans than non-matching IBD segments. This can be seen by the higher densities of matching IBD segments (pink) compared to densities of non-matching IBD segments (cyan) if the population proportions are not very close to zero — or, conversely, it can be seen at the lower peak at zero (less IBD segments that match the Denisova genome without Asians or European sharing). Many Denisova-matching IBD segments are shared exclusively among Asians which is indicated by the high density at a population proportion of 1 (pink density in Fig. S5). Population proportion of 1 means that IBD segments are only shared among Asians. Figs. S8 and S9 show analogous densities as in Figs. S5 and S6, but for the Neandertal genome. The differences we already observed for Denisova are even more prominent for Neandertal: Neandertal-matching IBD segments are observed even more often in Asians and Europeans than non-matching IBD segments. The higher densities (pink) are now more clearly observable if the population proportion is not close to zero — or conversely, it can be seen at the lower peak at zero (less IBD segments that match the Neandertal genome without Asians or European sharing). For Asians, the peak at 1 in Fig. S8 is very prominent, representing IBD segments that are shared exclusively among Asians. IBD segment sharing exclusively within one population is very common, as the blue peaks at 1 in Fig. S5 and Fig. S8 show. Figs. S7 and S10 show the same densities for Africans. Both the density of African proportions of Denisova-matching and the same density for Neandertal-matching IBD segments have two peaks: one at a low and one at a high proportion of Africans. For Neandertal-matching IBD segments, the density at low proportions of Africans is even larger than for high proportions. Thus, IBD segments that match archaic genomes are either shared by a very low or a very high proportion of Africans. The low proportion of African density peak hints at admixture of ancestors of modern humans and Denisovans / Neandertals outside of Africa. The density peak at high proportions of Africans may be be archaic DNA segments shared by hominid groups.

IBD segments that match the "Archaic genome" are the IBD segments that match both the Denisova and Neandertal genome. Population proportion densities for the "Archaic genome" are presented in Figs. S11, S12, and S13 for Asians, Europeans, and Africans, respectively. For the "Archaic genome" we see the same figure as for the Neandertal and Denisova genome: the African density is bimodal that means either it is dominated by Africans or it contains no or only few Africans.

We found that Japanese share parts of the Denisovan genome. An example of an IBD segment

that is shared by Japanese and that matches the Denisovan genome can be found in Fig. S26. Further examples can be seen in Figures S32 and S33.

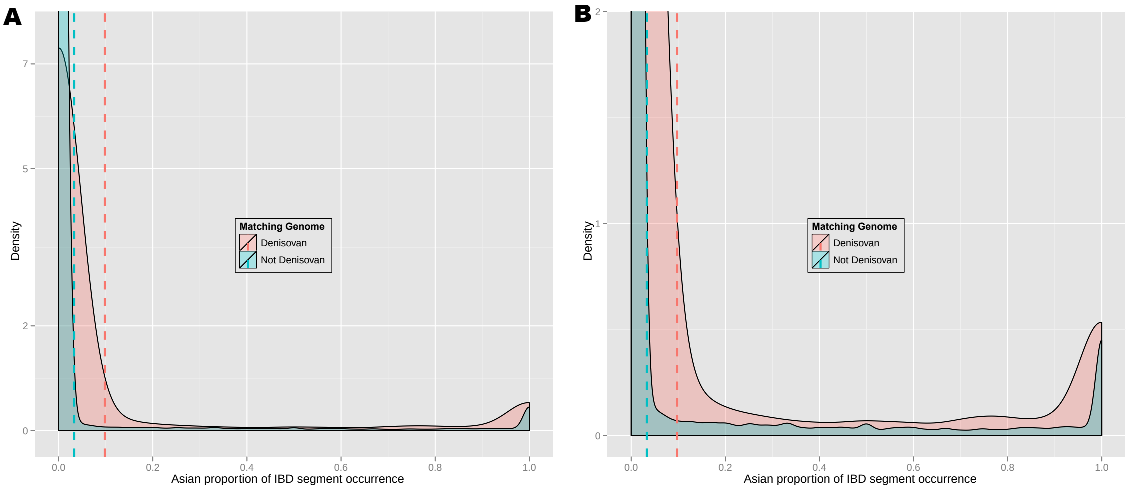

Figure S5: **Panel A**: Density of Asian proportions of Denisova-matching IBD segments (pink) vs. density of Asian proportions of non-Denisova-matching IBD segments (cyan). IBD segments were extracted from phased genotyping data of chromosome 1 of the 1000 Genomes Project. Dotted lines indicate the respective means. **Panel B**: The same density as in Panel A but zoomed in.

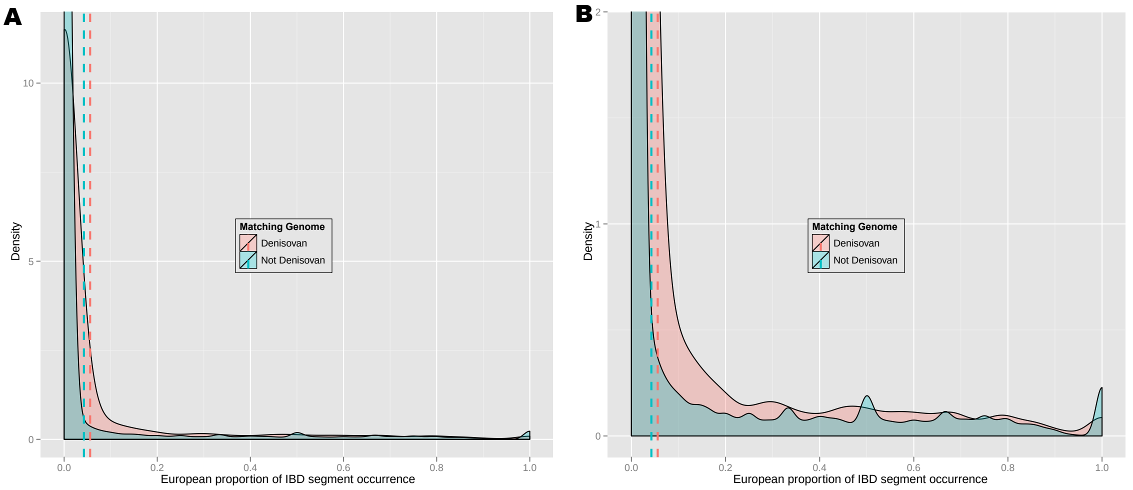

Figure S6: **Panel A**: Density of European proportions of Denisova-matching IBD segments (pink) vs. density of European proportions of non-Denisova-matching IBD segments (cyan). IBD segments were extracted from phased genotyping data of chromosome 1 of the 1000 Genomes Project. Dotted lines indicate the respective means. **Panel B**: The same density as in Panel A but zoomed in.

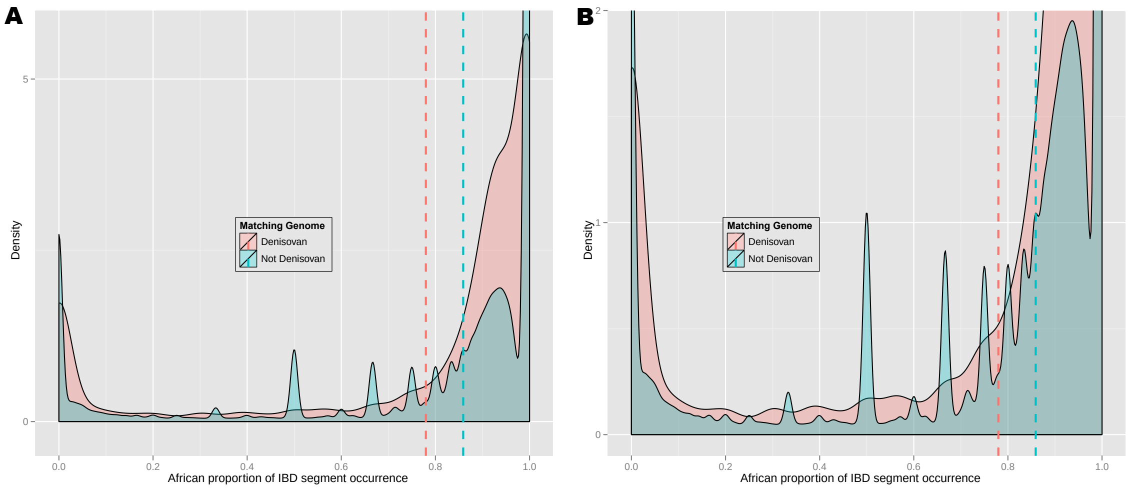

Figure S7: **Panel A**: Density of African proportions of Denisova-matching IBD segments (pink) vs. density of African proportions of non-Denisova-matching IBD segments (cyan). IBD segments were extracted from phased genotyping data of chromosome 1 of the 1000 Genomes Project. Dotted lines indicate the respective means. Peaks for non-Denisova-matching IBD segments are found at 0.5, 0.66, 0.33, 0.75, and 0.8, which corresponds to 1/2, 2/3, 1/3, 3/4, 4/5 (number of Africans / all individuals that have the IBD segment). The density of African proportions of Denisova-matching IBD segments has two peaks: one at a low and one at a high proportion of Africans. **Panel B**: The same density as in Panel A but zoomed in.

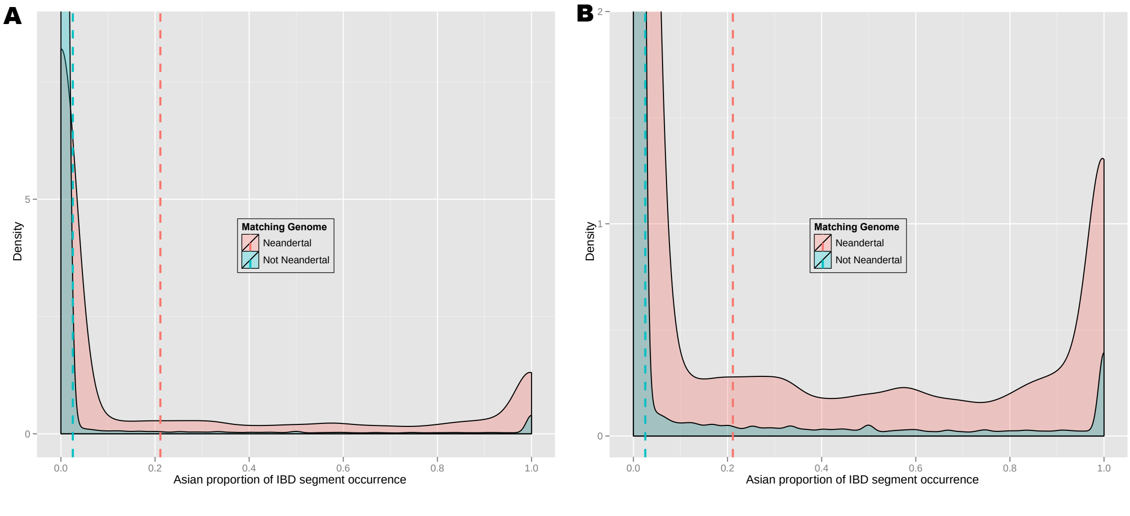

Figure S8: **Panel A**: Density of Asian proportions of Neandertal-matching IBD segments (pink) vs. density of Asian proportions of non-Neandertal-matching IBD segments (cyan). IBD segments were extracted from phased genotyping data of chromosome 1 of the 1000 Genomes Project. Dotted lines indicate the respective means. Many Neandertal-enriched IBD segments are shared mainly or exclusively by Asians as the proportion close to 1 shows. **Panel B**: The same density as in Panel A but zoomed in.

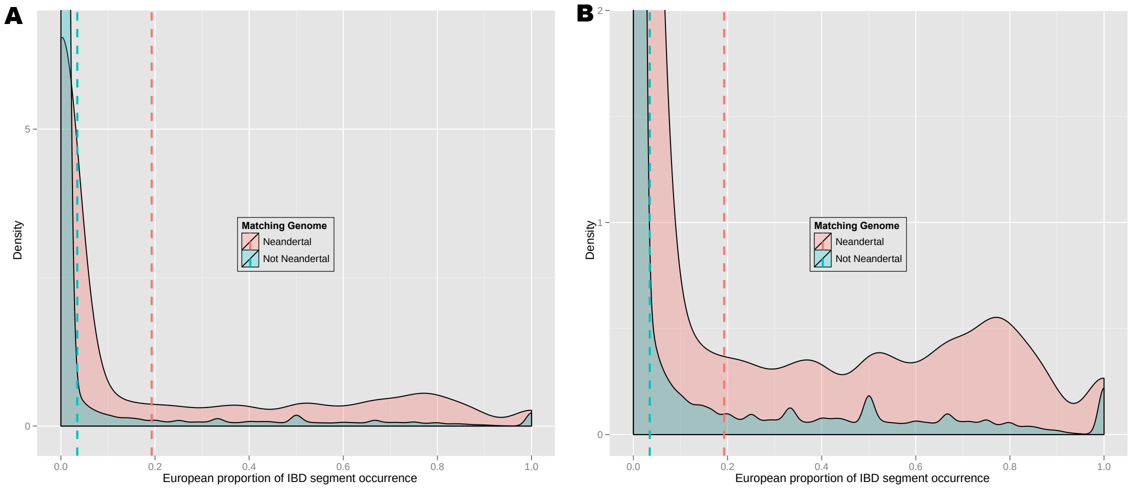

Figure S9: **Panel A**: Density of European proportions of Neandertal-matching IBD segments (pink) vs. density of European proportions of non-Neandertal-matching IBD segments (cyan). IBD segments were extracted from phased genotyping data of chromosome 1 of the 1000 Genomes Project. Dotted lines indicate the respective means. Many Neandertal-enriched IBD segments are shared mainly or exclusively by Asians as the proportion close to 1 shows. Neandertal-enriched IBD segments are shared by Europeans but fewer segments are shared exclusively among Europeans than for Asians (see Fig. S8). **Panel B**: the same density zoomed in.

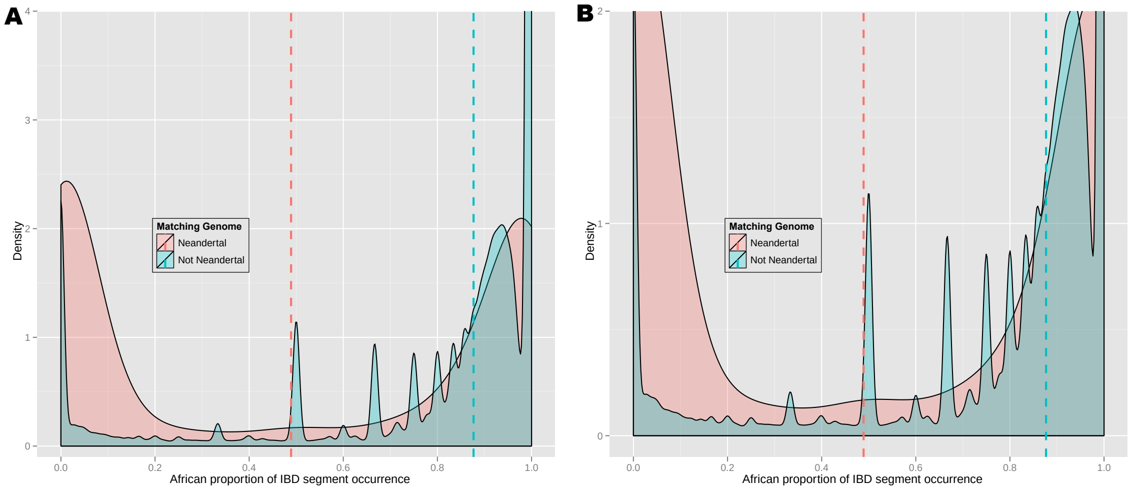

Figure S10: **Panel A**: Density of African proportions of Neandertal-matching IBD segments (pink) vs. density of African proportions of non-Neandertal-matching IBD segments (cyan). IBD segments were extracted from phased genotyping data of chromosome 1 of the 1000 Genomes Project. Dotted lines indicate the respective means. Peaks for non-Neandertal-matching IBD segments are found at 0.5, 0.66, 0.33, 0.75, and 0.8, which corresponds to 1/2, 2/3, 1/3, 3/4, 4/5 (number of Africans / all individuals that have the IBD segment). The density of African proportions of Neandertal-matching IBD segments has two peaks: one at a low and one at a high proportion of Africans. The density of low proportions of Africans is even larger than for high proportions. **Panel B**: the same density zoomed in.

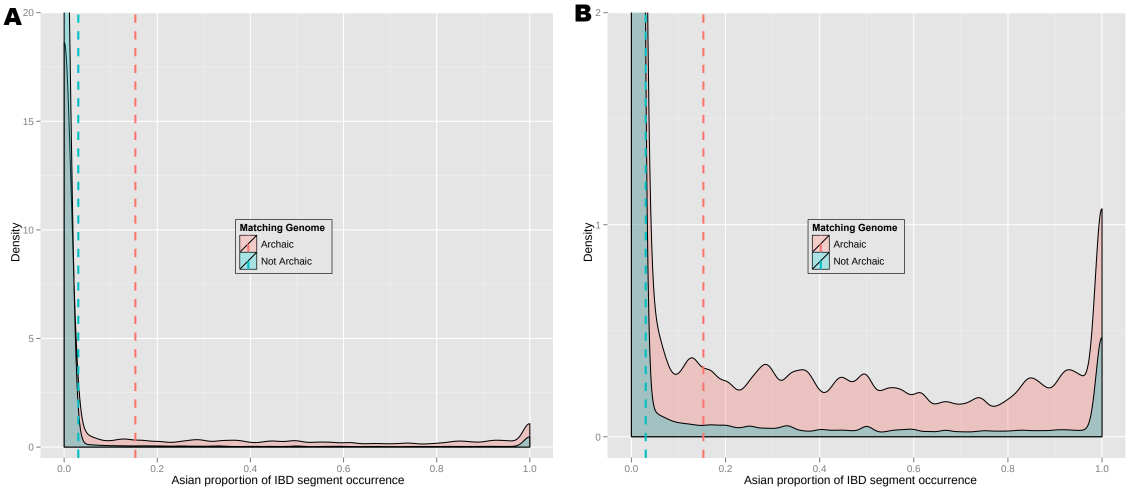

Figure S11: **Panel A**: Density of Asian proportions of Archaic-genome-matching IBD segments (pink) vs. density of Asian proportions of non-Archaic-genome-matching IBD segments (cyan). IBD segments were extracted from phased genotyping data of chromosome 1 of the 1000 Genomes Project. Dotted lines indicate the respective means. **Panel B**: the same density zoomed in.

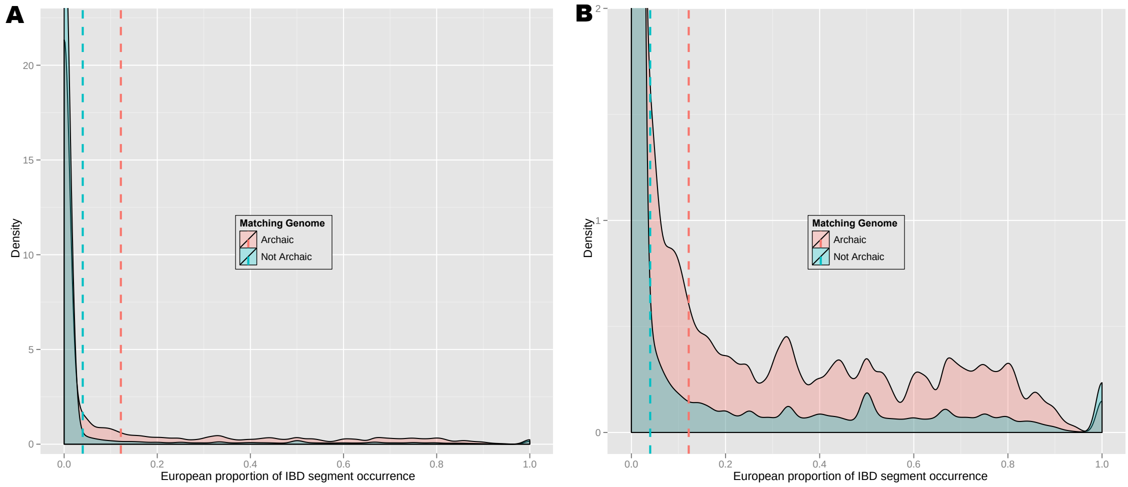

Figure S12: **Panel A**: Density of European proportions of Archaic-genome-matching IBD segments (pink) vs. density of European proportions of non-Archaic-genome-matching IBD segments (cyan). IBD segments were extracted from phased genotyping data of chromosome 1 of the 1000 Genomes Project. Dotted lines indicate the respective means. Many Archaic-genome-enriched IBD segments are shared mainly or exclusively by Asians as the proportion close to 1 shows. **Panel B**: the same density zoomed in.

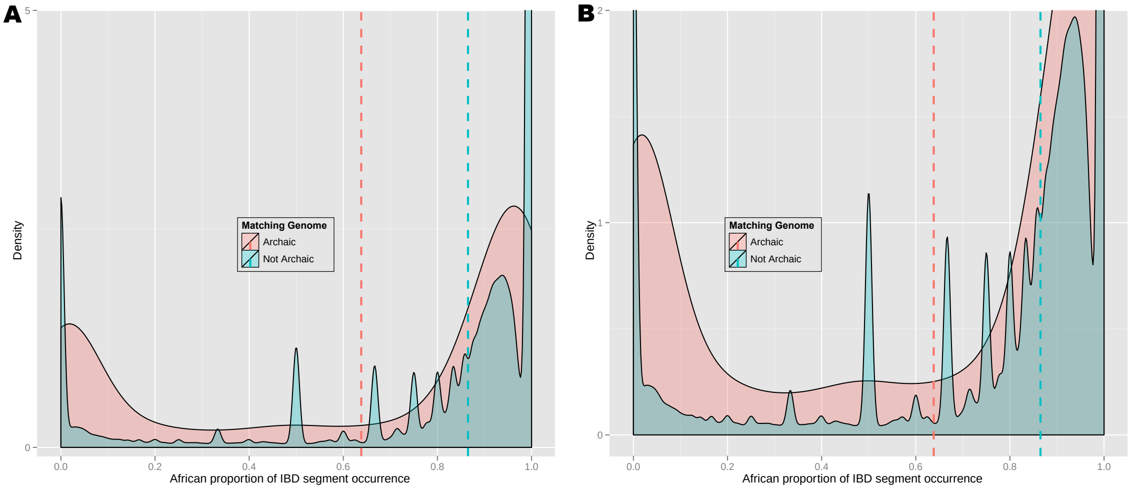

Figure S13: **Panel A**: Density of African proportions of Archaic-genome-matching IBD segments (pink) vs. density of African proportions of non-Archaic-genome-matching IBD segments (cyan). IBD segments were extracted from phased genotyping data of chromosome 1 of the 1000 Genomes Project. Dotted lines indicate the respective means. Peaks for non-Archaic-genome-matching IBD segments are found at 0.5, 0.66, 0.33, 0.75, and 0.8, which corresponds to 1/2, 2/3, 1/3, 3/4, 4/5 (number of Africans / all individuals that have the IBD segment). The density of African proportions of Archaic-genome-matching IBD segments has two peaks: one at a low and one at a high proportion of Africans. **Panel B**: the same density zoomed in.

S13 Analyses of Lengths of IBD Segments

S13.1 Relating the IBD Length to Years from Present

The lengths of an IBD segment are exponentially distributed with a mean of $100/(2g)$ cM (centiMorgans), where g is the number of generations which separate the two haplotypes that share a segment from their common ancestor (21, 26, 27, 28, 34). We follow Ulgen and Li (35), who recommend to use a cM-to-Mbp ratio of 1. However, the recombination rate, the cM-to-Mbp ratio, varies from 0 to 9 along a chromosome (36). An average IBD segment length of 10 kbp corresponds to a most recent common ancestor 5,000 generations or 100 thousand years ago (kya) using 20 years per generation, and 20 kbp corresponds to 2,500 generations or 50 kya. Therefore, individuals sharing short IBD segments provide insight into the origin and migration of modern humans.

Age estimations of the IBD segments should be considered with care. Computing the time since the last common ancestor depends on many assumptions like the time span of a generation, the ratio of centiMorgans and megabases which we assumed to be 1, and the distribution of IBD lengths.

S13.2 Correction of the Length-Year Relation For Ancient Genomes

The genomes of the Neandertals and the Denisovans are older than present human genomes, therefore the assumptions that g generations separate the first haplotype and g the second haplotype from a common ancestor are wrong.

The Neandertal bones from which the DNA was extracted were dated to 38,310 and 44,450 years before present day by carbon-14 accelerator mass spectrometry (37). This gives an average of 40 kya, which corresponds to 2,000 generations. The corrected formula for the average IBD segment length would be $100/(2g - 2000)$ cM. The Denisovan finger bone from which the Denisovan DNA was sequenced is also dated back about 40 kya. Thus, 10 kbp correspond to 6,000 generations or to 120 kya and 20 kbp to 3,500 generations or 70 kya. The correction described above adds 1,000 generations or 20 kya as compared to the number if two present day genomes would have been compared, that is, if human IBD would be considered.

S13.3 Histograms of Lengths for the Different Genomes

Figure S14 show the histograms of IBD segment lengths for all IBD segments (human genome) and for IBD segments that match the Neandertal genome. For the human genome a peak is at 24,200 bp (41 kya), whereas, for the Neandertal genome, peaks are at 18,500 (74 kya) and 43,000 bp (43 kya). It can be seen that IBD segments that match the Neandertal genome are shorter, thus also older, though there is also an enrichment for longer IBD segments around 43,000 bp.

Figure S15 show the histograms of IBD segment lengths for all IBD segments that match the Denisova and the “Archaic” genome (“Archaic genome” contains IBD segments that match both the Denisova and Neandertal genome). For the Denisova genome, we have peaks at 24,200 bp (61 kya) and 9,000 bp (131 kya), whereas, for the Archaic genome, peaks are at 11,200 bp (109 kya), 24,200 bp (61 kya), and 41,000 bp (44 kya). It can be seen that IBD segments that match the

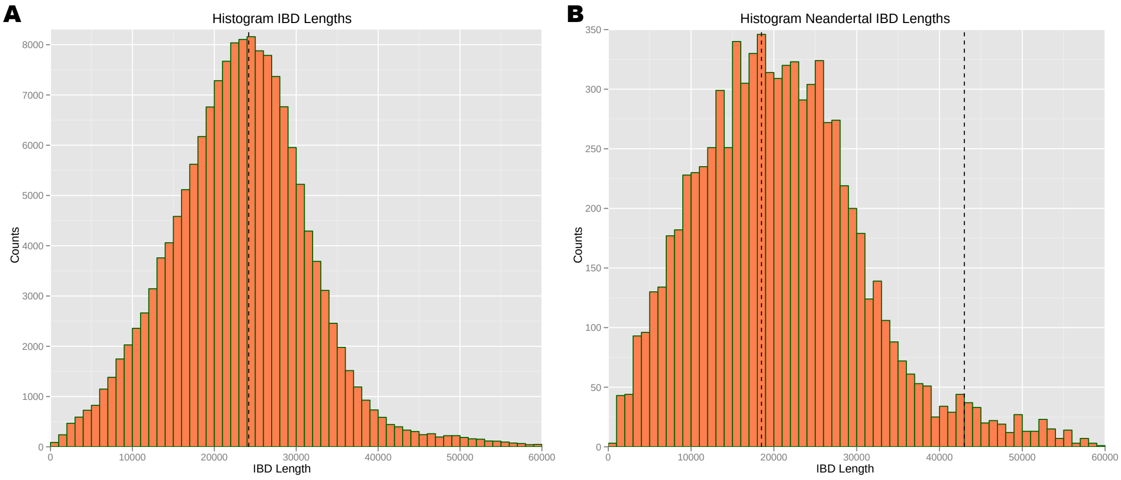

Figure S14: **Panel A**: Histogram of the IBD segment lengths for all IBD segments found in the 1000 Genomes Project data (human genome). The global peak is at 24,200 bp. **Panel B**: Histogram of the IBD segment lengths for IBD segments that match the Neandertal genome. Peaks at 18,500 bp and 43,000 bp are indicated.

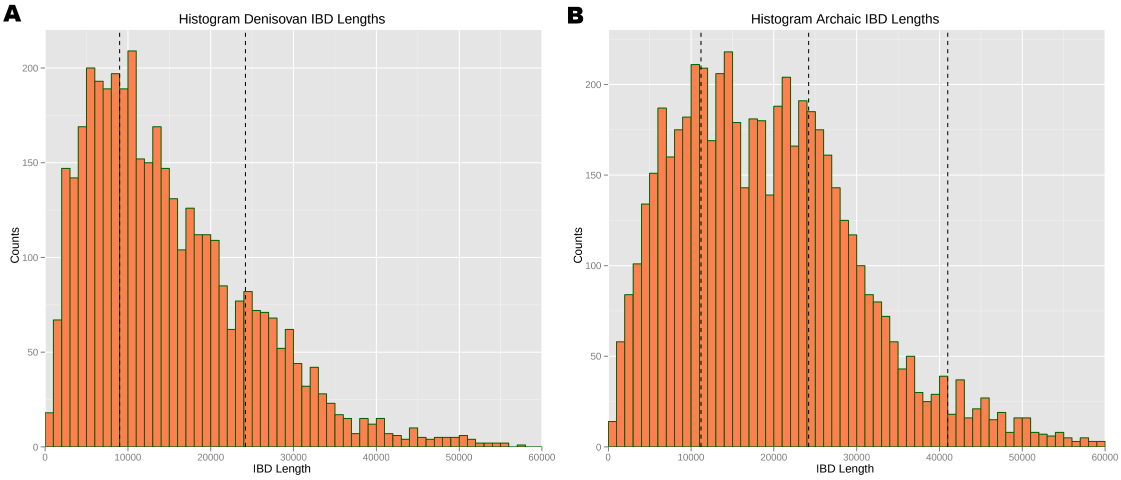

Figure S15: **Panel A**: Histogram of the IBD segment lengths for IBD segments that match the Denisova genome. Peaks at 9,000 bp and 24,200 bp are indicated. **Panel B**: Histogram of the IBD segment lengths for IBD segments that match both the Neandertal and the Denisova genome ("Archaic genome"). Peaks at 11,200 bp, 24,200 bp , and 41,000 bp are indicated.

Neandertal genome are shorter, thus also older, though there is also an enrichment for longer IBD segments around 43,000 bp.

S13.4 IBD Segment Lengths of Human Populations

Figure S16 shows the density of the lengths of IBD segments that are private to Asians vs. the density of the lengths of IBD segments that are private to Europeans. Since IBD segments are private to each population, the densities are based on disjoint IBD segment sets. Asians show a global peak at 25,800 bp (39 kya in the past from present), while Europeans show a global peak at 24,200 bp (41 kya). Europeans have also smaller peaks at 17,000 bp (59 kya) and 29,000 bp (34.5 kya).

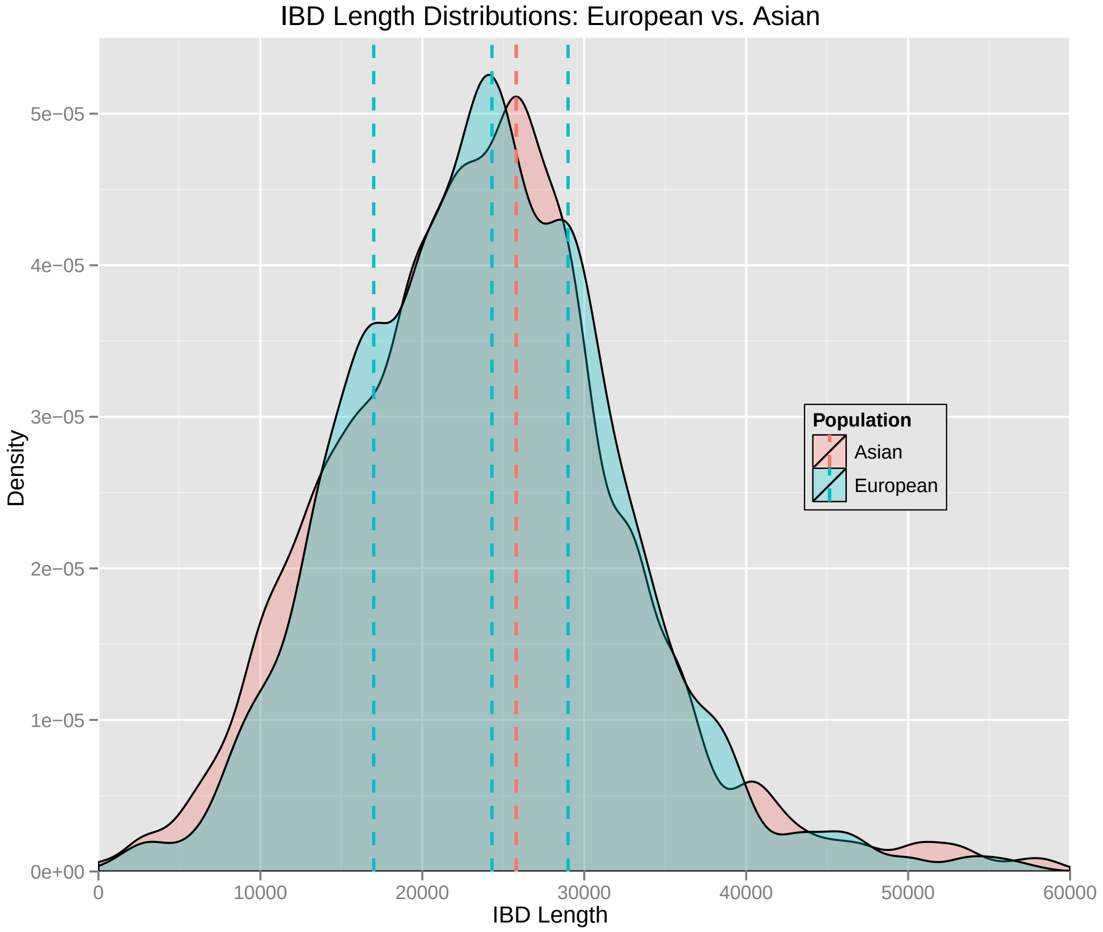

Figure S16: Density of lengths of IBD segments that are private to Asians vs. the analogous density for Europeans. Interesting peaks are marked by dashed lines. Asians have a global peak at 25,800 bp (red), while Europeans have the global peaks at 24,200 bp (blue). Europeans have smaller peaks at 17,000 bp and 29,000 bp (blue).

Figure S17A shows the density of lengths of IBD segments that are private to Asians vs. lengths of IBD segments that are only shared between Asians and Africans. Figure S17B shows the same plot for Europeans instead of Asians. A small difference is visible in the global peaks of lengths distributions of IBD segments that are private to a population and those shared with

Africans (blue dashed lines): 25,800 bp (39 kya) vs. 22,200 (45 kya) for Asians and 24,200 bp (41 kya) vs. 22,000 (45.5 kya) for Europeans. Interestingly, even though the IBD segments used in both plots are disjoint, African-sharing IBD segment lengths distributions agree in their major peak at about 22,000 bp (45.5 kya) and in a peak at 5,000 bp (200 kya). The global Asian peak is at 25,800 bp (red dashed line in Fig. S17A), while the global peak for Europeans is at 24,200 bp (red dashed line in Fig. S17B). Europeans in Fig. S17B have additional peaks at 16,500 bp and 28,900 bp. The peak at 5,000 bp is in a range where African-sharing IBD segment lengths are enriched. This range is from 3,000 bp (333 kya) to 10,000 bp (100 kya). We follow up on the 5,000 bp peak by investigating the lengths of IBD segments that are shared by Asians, Europeans, and Africans.

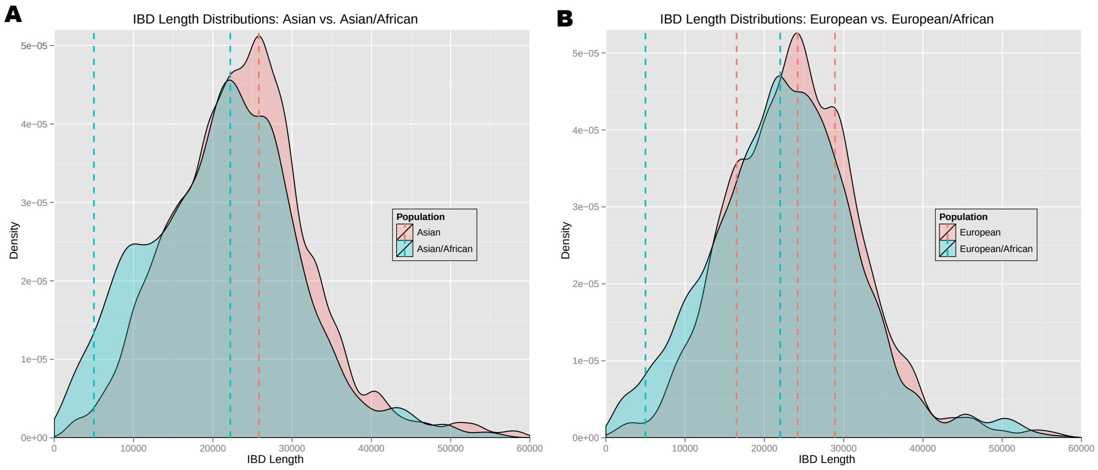

Figure S17: **Panel A**: Density of lengths of IBD segments that are private to Asians vs. density of IBD segment lengths shared only by Asians and Africans. **Panel B**: Density of lengths of IBD segments that are private to Europeans vs. density of IBD segment lengths shared only by Europeans and Africans. Both for African-Asian and for African-European IBD segments, peaks coincide at 5,000 bp and at about 22,000 bp (blue dashed lines). The global Asian peak is at 25,800 bp (red dashed line in panel A), while the global peak for Europeans is at 24,200 bp (red dashed line in panel B). Europeans (in panel B) have additional peaks at 16,500 bp and 28,900 bp.

Figure S18A shows the density of lengths of IBD segments that are private to Asians vs. lengths of IBD segments that are shared between Asians, Europeans, and Africans. Figure S18B shows the same plot for Europeans instead of Asians. Of course, the peaks for Asians and Europeans are the same as in the previous plot. The peak at 5,000 bp (200 kya) is also visible for lengths of IBD segments that are shared by all populations and which marks the higher density range 3,000–10,000 bp (333–100 kya). IBD segments that are shared by all populations seem to be very old. Fu et al. (32) found that the SNVs shared between European Americans and African Americans date back 104,400 and 115,800 years, respectively. We would date our rare tagSNVs even further back. However, the estimated age of the IBD segments should be considered with care. The computation of the time since the last common ancestor depends on many assumptions like the length of a generation, the recombination rate (ratio between centiMorgans and megabases), and the distribution of IBD lengths.

Next we investigated the effect on the lengths distribution if IBD segments are removed that

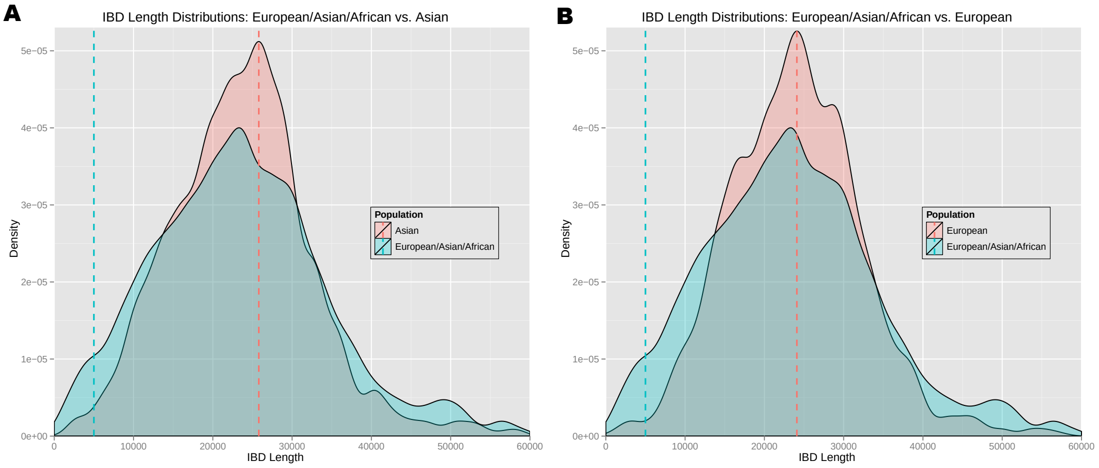

Figure S18: **Panel A**: Density of lengths of IBD segments that are private to Asians vs. density of IBD segment lengths shared by Asians, Europeans, and Africans. **Panel B**: Density of lengths of IBD segments that are private to Europeans vs. density of IBD segment lengths shared by Asians, Europeans, and Africans. The peak at 5,000 bp is again visible marking the range 3,000–10,000 bp, where the density of lengths of shared IBD segments is larger (blue).

are shared by Africans. Figure S19A shows the density of lengths of IBD segments that are private to Asians vs. the density of IBD segment lengths shared by Asians and Europeans, but not by Africans. In Fig. S18B, the same plot as in Fig. S18A is shown, but now compared to IBD segments that are private to Asians. In Fig. S18A the density for IBD segments that are shared with Africans, has again a peak at 5,000 bp and high values in the range 3,000–10,000 bp (blue). In Fig. S18B, this peak vanishes, because IBD segments that are shared with Africans are removed. A density peak at 50,000 bp (20 kya) becomes very prominent for the lengths of IBD segments that are shared between Europeans and Asians, but not with Africans. The density of lengths of IBD segments shared between Europeans and Asians (blue) is higher between 35,000 bp (28.5 kya) and 55,000 bp (18 kya) than all lengths densities of IBD segment that are shared by Africans
.

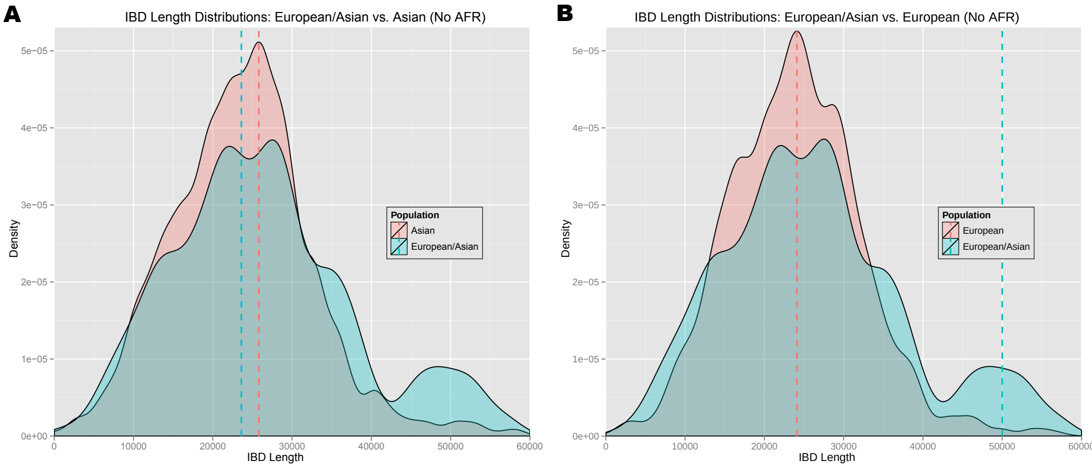

Figure S19: **Panel A**: Density of lengths of IBD segments that are private to Asians vs. density of IBD segment lengths shared by Asians and Europeans but not by Africans that means, African-sharing IBD segments are removed as opposed to Fig. S18A. **Panel B**: The same plot as in panel A but, but IBD segments that are shared by Europeans and Asians are compared to IBD segments that are private to Europeans. African-sharing IBD segments are removed as opposed to Fig. S18B. Both panels show that removing IBD segments shared with Africans in turn remove the peak at 5,000 bp and the high density range 3,000–10,000 bp which were visible in Fig. S18. In panel B a peak at 50,000 bp (blue) becomes very prominent for lengths of IBD segments shared between Europeans and Asians (no Africans).

S13.5 Lengths of IBD Segments that Match the Denisova or Neandertal Genome

We investigated the age of IBD segments that match the Denisova or Neandertal genome. Toward this end, we consider the lengths distributions of IBD segments that match the Denisova or the Neandertal genome.

S13.5.1 Lengths of IBD Segments that Match the Denisova Genome

Figure S20A shows the density of the lengths of all IBD segments (human genome) vs. the density of the lengths of IBD segments that match the Denisovan genome. The human IBD segment lengths density has a peak at 24,200 bp (41 kya), while the lengths distribution of IBD with the Denisovan genome has a peak at 9,000 bp (131 kya). Clearly, segments of IBD with the Denisovan genome are shorter than among humans (red density above blue at the left hand side). We are interested in how different populations share the Denisovan genome. Figure S20B shows densities of lengths of IBD segments that match the Denisova genome and are enriched by a particular population. For Africans, the density peak is at 8,500 bp (137 kya). Asians have a clear enrichment of IBD lengths in the range 18,000–28,000 bp (75–56 kya). Europeans show three peaks at 18,000 (75 kya), 25,500 (59 kya), and 40,000 (45 kya). This density seems to reveal that there was a gene flow from Denisovans into the Asian genome outside Africa. However, also Europeans show some hints of introgression from the Denisovans after migration out of Africa.

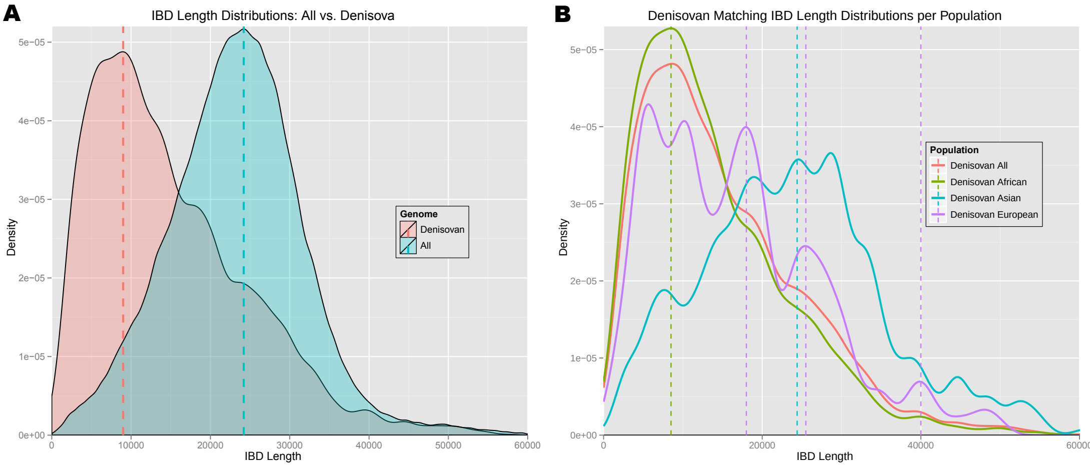

Figure S20: **Panel A**: Density of the lengths of all IBD segments vs. the lengths of IBD segments that match the Denisova genome. The dashed lines indicate the peaks at 9,000 bp for Denisovan and 24,200 bp for human genome. **Panel B**: Densities of lengths of IBD segments that match the Denisova genome and are enriched in a particular population. The dashed lines indicate the density peaks at 8,500 bp for for Africans; 24,400 bp for Asians (range 18,000–28,000 bp); 18,000, 25,500, and 40,000 bp for Europeans.

These peaks can be seen more clearly if only IBD segments are considered that are private to a population. Figure S21A shows densities of lengths of IBD segments that match the Denisova genome and are private to a population. For all populations together, the peak of the lengths density is at 8,500 bp (137 kya); for IBD segments private to Africans, it is at 9,700 (123 kya);

for IBD segments private to Europeans, it is at 14,800 (87 kya); and for IBD segments private to Asians, it is at 27,000 (57 kya). The density of IBD lengths that match the Denisova genome and are private to Asians is also high around 40,000 bp (45 kya). Figure S21B shows densities of lengths of IBD segments that match the Denisova genome and are private to Africans vs. IBD segments that are not observed in Africans. The peak for Africans is at 10,000 bp (120 kya), while the density of lengths of IBD segments that are not observed in Africans has peaks at 20,000 (70 kya) and 28,000 bp (56 kya). Africans have older segments probably stemming from common ancestors of Denisovans and humans. For non-African populations, the high densities for longer IBD segments hint at an introgression from Denisovans after migration out of Africa. Parts of the Denisovan or archaic genomes may also have been introduced by Neandertals after migration out of Africa. Neandertals may have re-introduced parts of archaic genomes that were lost in humans or parts of the Denisovan genome stemming from introgression of one hominid group into another.

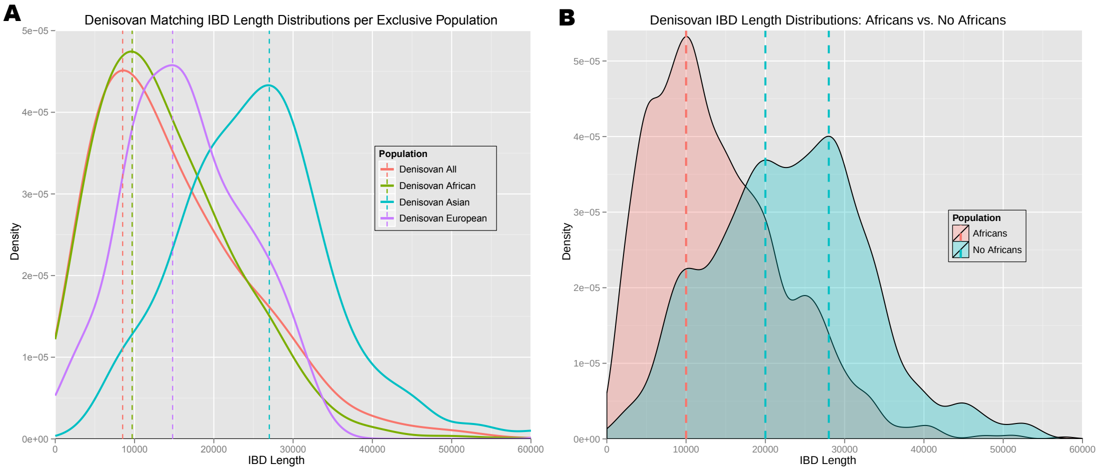

Figure S21: **Panel A**: Densities of lengths of IBD segments that match the Denisova genome and are private to a population. The peaks are at 8,500 for ll IBD segments matching the Denisova genome, 9,700 for Africans, 14,800 for Europeans, and 27,000 for Asians. **Panel B**: Densities of lengths of IBD segments that match the Denisova genome and are private to Africans vs. IBD segments that are not observed in Africans. The peak for Africans is at 10,000 bp, while IBD segment lengths that are not observed in Africans have peaks at 20,000 and 28,000 bp.

S13.5.2 Lengths of IBD Segments that Match the Neandertal Genome

Figure S22A shows the density of the lengths of all IBD segments (human genome) vs. the density of the lengths of IBD segments that match the Neandertal genome. The human IBD segment lengths density has a peak at 24,200 bp (41 kya), while the lengths distribution of IBD segments with the Neandertal genome (the part of the IBD segment that matches the Neandertal genome) has a global peak at 18,000 bp (75 kya) and a smaller peak at 43,000 bp (43 kya). Figure S22B shows densities of lengths of IBD segments that match the Neandertal genome and are enriched in a particular population. The peak of the lengths distribution for IBD segments that are shared by Africans is at 17,000 bp (79 kya). Asians have a density peak at 25,800 bp (59 kya) and Europeans have a peak at 24,000 bp (62 kya). Both the densities of lengths of IBD segments that are private to Europeans and that are private to Asians and match the Neandertal genome have a peak around

42,000 bp (44 kya). The density peak for Africans is clearly separated from the density peaks for Europeans and Asians which match each other. This hints to introgression from the Neandertals into anatomically modern humans that were the ancestors of Europeans and Asians after these humans left Africa. The higher density of short IBD segments which are prominent in Africans in the range 5,000–15,000 bp (220–87 kya) hint at old DNA segments that humans share with the Neandertal genome.

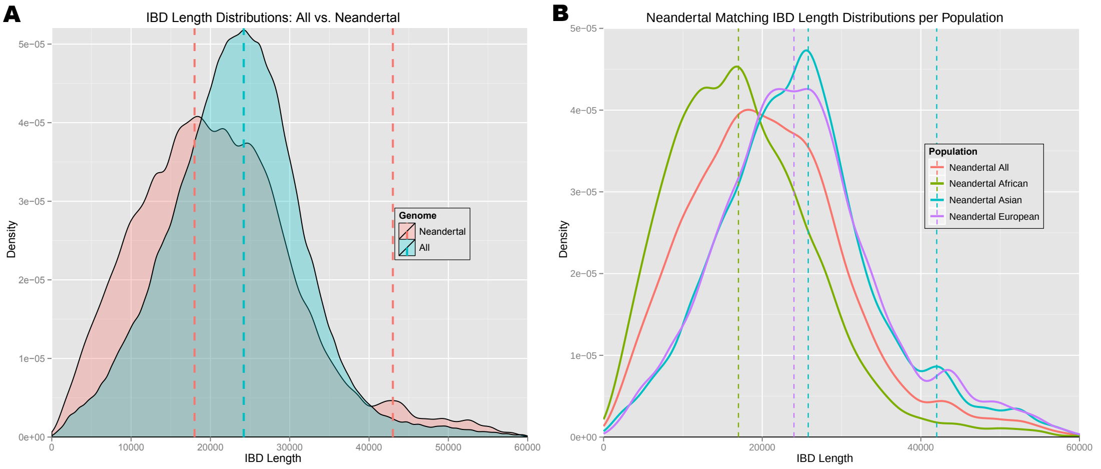

Figure S22: **Panel A**: Density of the lengths of all IBD segments vs. the lengths of IBD segments that match the Neandertal genome. The dashed lines indicate the peaks of densities at 18,000 bp and 24,200 bp as well as the smaller peak at 43,000 bp. **Panel B**: Densities of lengths of IBD segments that match the Neandertal genome and are enriched in a particular population. The dashed lines indicate the density peaks at 17,000 bp for Africans, 25,800 bp for Asians, and 24,000 bp for Europeans. Further a smaller peak for both Europeans and Asians is visible at 42,000 bp.

Next we consider IBD segments that are private to a population. Figure S23A shows densities of lengths of IBD segments that match the Neandertal genome and are private to a population. The peaks are at 15,500 bp (84.5 kya) for Africans, at 19,500 bp (71 kya) for all humans, at 22,800 bp (64 kya) for Europeans, and at 23,800 bp (62 kya) for Asians. The densities of Asians and Europeans agree very well to each other. The peak close to 43,000 bp is no longer visible and seems to have been caused by IBD segments that are shared between Asians and Europeans. Therefore, we are interested in IBD segments that match the Neandertal genome and that are private to Africans and those which are not observed in Africans. Figure S23B shows densities of lengths of IBD segments that match the Neandertal genome and are private to Africans vs. IBD segments that are not observed in Africans. The peak for Africans is at 17,000 bp (79 kya), while IBD segments that are not observed in Africans have a global lengths density peak at 23,800 (62,000 years) and a smaller peak at 21,000 bp (68 kya). Interestingly, the peak near 43,000 bp (43 kya) is again visible. Thus, this peak is from IBD segments that are shared between Europeans and Asians. Most prominently, non-African IBD segments that match the Neandertal genome are enriched in the range 40,000–55,000 bp (45–38 kya).

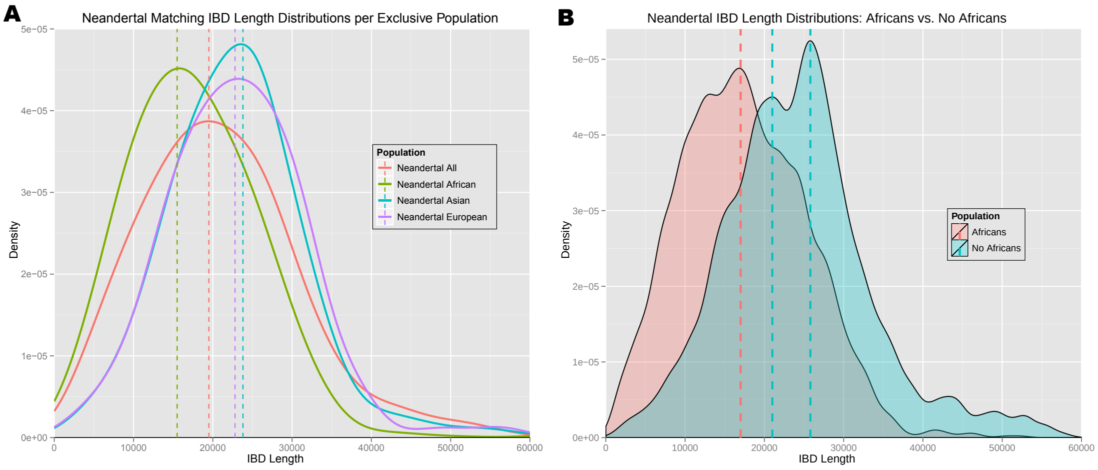

Figure S23: **Panel A**: Densities of lengths of IBD segments that match the Neandertal genome and are private to a population. The peaks are at 15,500 bp for Africans, 19,500 bp for all humans, 22,800 bp for Europeans, and 23,800 bp for Asians. **Panel B**: Densities of lengths of IBD segments that match the Neandertal genome and are private to Africans vs. IBD segments that are not observed in Africans. The peak for Africans is at 17,000 bp, while IBD segments that are not observed in Africans have a global lengths density peak at 23,800 and a smaller peak at 21,000 bp. The peak near 43,000 bp is again visible. Most prominently, non-African IBD segments that match the Neandertal genome are enriched in the range 40,000–55,000 bp (blue density).

S13.5.3 Lengths of IBD Segments that Match an Archaic Genome

IBD segments that match the "Archaic genome" are IBD segments that match both the Neandertal and the Denisova genome. The archaic genome segments stem either from a genome of archaic hominids which were ancestors of Neandertals and Denisovans or stem from introgression of one hominid group into another. Hammer et al. (38) write

> "The observation that populations from many parts of the world, including Africa, show evidence of introgression of archaic variants (6, 16, 19) suggests that genetic exchange between morphologically divergent forms may be a common feature of human evolution."

Elizabeth Pennisi (39) writes:

> "The analyses paint a complex picture of mingling among ancient human groups, Pääbo reported. The data suggest inbreeding in Neandertals, a large Denisovan population, and mixing between Denisovans and an even earlier mystery species."

Figure S24A shows the density of the lengths of all IBD segments (human genome) vs. the density of the lengths of IBD segments that match the Archaic genome. For the human genome, the global density peak is at 24,200 bp (61 kya). For IBD with the Archaic genome, the global density peak is at 11,200 bp (103 kya), but a smaller peak at 41,000 bp (103 kya) can be observed. Figure S24B shows densities of lengths of IBD segments that match the Archaic genome and are enriched in a particular population. IBD lengths density peaks can be seen at 12,000 bp (103 kya)

for most populations, 22,000 bp (65.5 kya) for Europeans, and 24,400 bp (61 kya) for Asians. The global peak for Africans is not separated from the global peaks of non-Africans, as we observed in Neandertal- or Denisova-matching IBD segments. Interestingly, a smaller peak is visible for both Europeans and Asians at 42,000 bp (44 kya). This peak at 42,000 bp has already been observed in Neandertal-matching IBD segments. There may be Neandertal matching IBD segments that do not match the single Denisova genome that has been sequenced, but might match other Denisova genomes. Therefore, some Neandertal matching segments might be also old and would belong to the "Archaic genome" matching IBD segments.

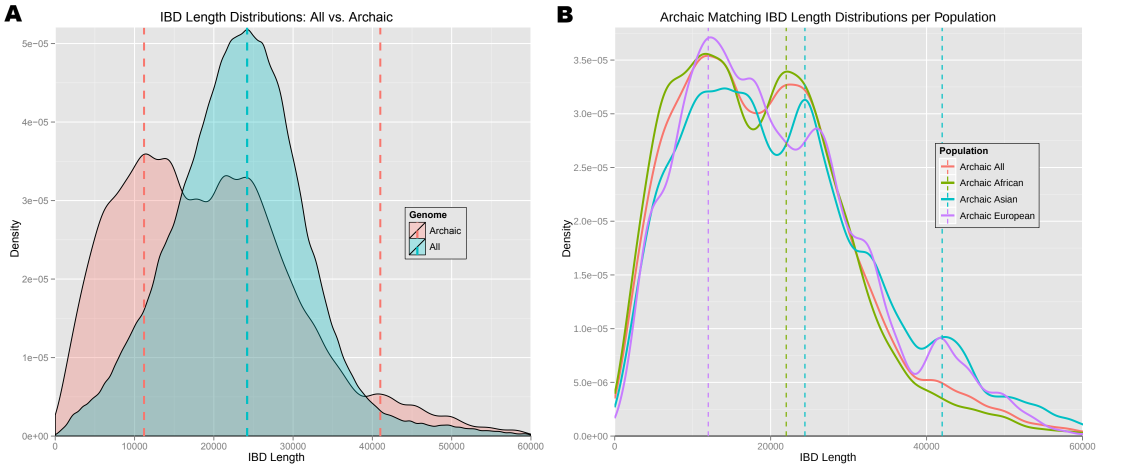

Figure S24: **Panel A**: Density of the lengths of all IBD segments vs. the lengths of IBD segments that match the Archaic genome. The dashed lines indicate the peaks of densities at 11,200 bp and 24,200 bp as well as the smaller peak at 41,000 bp. **Panel B**: Densities of lengths of IBD segments that match the Archaic genome and are enriched in a particular population. The dashed lines indicate the density peaks at 12,000 bp for most populations, 22,000 bp for Europeans, and 24,400 bp for Asians. Further, a smaller peak is visible for both Europeans and Asians at 42,000 bp.

Next we consider IBD segments that are private to a population. Figure S25A shows densities of lengths of IBD segments that match the Archaic genome and are private to a population. The peaks are at 13,000 bp (97 kya) for humans (1000 Genomes Project), 13,600 bp (93.5 kya) for Europeans, 19,500 bp (71 kya) for Asians, and 21,000 (68 kya) for Africans. A peak appears at 46,000 bp (42 kya) for IBD segments that are private to Europeans. Figure S25B shows densities of lengths of IBD segments that match the Archaic genome and are private to Africans vs. IBD segments that are not observed in Africans. The peak for Africans is at 22,000 bp (65.5 kya), while lengths of IBD segments that are not observed in Africans have a global peak at 14,700 bp (88 kya). However, Africans have also a peak at 14,300 bp (90 kya), while IBD segments that are not observed in Africans have a peak at 21,000 (68 kya). These peaks match quite well on a global scale. Most prominently, non-African IBD segments that match the Archaic genome are enriched in the range 33,000–55,000 bp (50–38 kya). This enrichment seem to be caused by events after humans migrated out of Africa. The introgression from the Neandertal into ancestors of modern humans may also have introduced a part of the Denisovan genome that has been contained in the Neandertal genome. We would consider this part of the human genome as Archaic genome. Another explanation would be that introgression from the Neandertal into human has

introduced archaic genome parts that were lost in the human population in Africa, but still match the Denisovan genome.

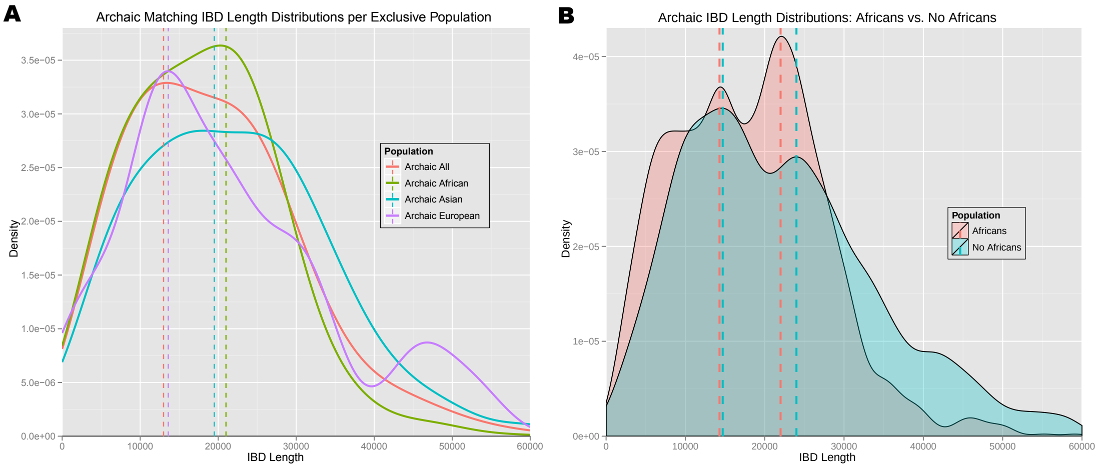

Figure S25: **Panel A**: Densities of lengths of IBD segments that match the Archaic genome and are private to a population. The peaks are at 13,000 bp for humans, 13,600 bp for Europeans, 19,500 bp for Asians, and 21,000 for Africans. A peak appears at 46,000 bp for Europeans. **Panel B**: Densities of lengths of IBD segments that match the Archaic genome and are private to Africans vs. IBD segments that are not observed in Africans. The peak for Africans is at 22,000 bp, while the lengths of IBD segments that are not observed in Africans have a global peak at 14,700 bp. However, Africans have also a peak at 14,300 bp, while IBD segments that are not observed in Africans have a peak at 21,000 bp. These peaks match quite well. Most prominently, non-African IBD segments that match the Archaic genome are enriched in the range 33,000–55,000 bp (blue density).

S14 Additional Results

S14.1 Other Thresholds for Matching an Archaic Genome

In the main manuscript, we defined an IBD segment to match a particular archaic genome if the genome proportion is 30% or higher. Instead of 30%, we also tested thresholds of 40% and 50%, which resulted in odds ratios of 5.4 and 5.8 with p-values $<$ 1.9e-272 and $<$ 1.3e-252, respectively, for the dependency between the Asian population and the Denisova genome. Hence, we obtain the same figures with other thresholds for defining an IBD segment to match an archaic genome.

S14.2 Comparison Results Based on Mean Instead of the Median

We report the results of the comparisons using the mean instead of the median. The p-values are still based on the Wilcoxon rank sum test over the 100 experiments.

Table S10: Comparison of IBD detection methods for short segments on simulated data in terms of power (true positive rate, sensitivity, or recall).

Method	artA100		artB100		artC100		artD100	
	mean	p	mean	p	mean	p	mean	p
HapFABIA	0.84	——	0.60	——	0.78	——	0.56	——
fastIBD-1	0.98	5.5e-16	0.23	5.8e-12	0.28	4.4e-18	0.21	3.9e-18
fastIBD-2	0.96	3.4e-13	0.09	2.5e-15	0.11	3.9e-18	0.06	3.9e-18
PLINK	0.98	3.9e-18	0.96	4.8e-18	0.99	3.9e-18	0.97	3.9e-18
GERMLINE-1	0.28	4.0e-18	0.79	8.4e-06	0.76	9.3e-02	0.32	3.9e-18
GERMLINE-2	0.16	3.9e-18	0.53	4.1e-02	0.49	7.0e-18	0.17	3.9e-18
DASH	0.20	3.9e-18	0.75	3.9e-18	0.71	3.9e-18	0.27	3.9e-18
Method	artAMis		artBMis		artCMis		artDMis	
	mean	p	mean	p	mean	p	mean	p
HapFABIA	0.82	——	0.53	——	0.77	——	0.56	——
fastIBD-1	0.98	6.8e-17	0.27	1.0e-07	0.31	3.9e-18	0.20	3.9e-18
fastIBD-2	0.95	1.9e-12	0.12	2.4e-13	0.11	3.9e-18	0.06	3.9e-18
PLINK	0.97	1.1e-17	0.95	4.0e-18	0.99	3.9e-18	0.97	3.9e-18
GERMLINE-1	0.31	5.3e-18	0.78	7.7e-09	0.75	2.6e-02	0.32	3.9e-18
GERMLINE-2	0.14	3.9e-18	0.52	4.3e-01	0.47	4.8e-18	0.16	3.9e-18
DASH	0.22	3.9e-18	0.76	3.3e-08	0.69	3.9e-18	0.26	3.9e-18
Method	artA		artB		artC		artD	
	mean	p	mean	p	mean	p	mean	p
HapFABIA	0.83	——	0.46	——	0.81	——	0.53	——
fastIBD-1	0.99	4.6e-18	0.97	5.3e-18	0.99	3.9e-18	0.98	3.9e-18
fastIBD-2	0.97	1.7e-13	0.82	3.3e-13	0.96	2.2e-17	0.93	3.9e-18
PLINK	0.98	1.0e-17	0.99	4.4e-18	0.99	3.9e-18	0.99	3.9e-18
GERMLINE-1	0.51	3.5e-13	0.84	5.3e-13	0.83	1.0e+00	0.52	2.2e-02
GERMLINE-2	0.33	3.9e-16	0.60	2.3e-03	0.60	1.5e-16	0.33	3.9e-18
DASH	0.47	3.9e-18	0.81	3.9e-18	0.79	3.9e-18	0.47	3.9e-18

Columns labeled "mean" show the mean power over 100 experiments. Columns labeled "p" provide the p-values of a Wilcoxon rank sum test over the 100 experiments with the null hypothesis that HapFABIA and another method yield the same value for the power. HapFABIA has significantly lower power than other methods but only by approximately a factor of 2. PLINK has the highest power.

Table S11: Comparison of IBD detection methods for short segments on simulated data in terms of false discovery rate (1 - precision).

Method	artA100		artB100		artC100		artD100	
	mean	p	mean	p	mean	p	mean	p
HapFABIA	0.07	——	0.07	——	0.12	——	0.15	——
fastIBD-1	0.98	3.9e-18	0.99	5.5e-18	0.95	3.9e-18	0.95	3.9e-18
fastIBD-2	0.88	3.9e-18	0.97	4.3e-18	0.83	3.9e-18	0.91	3.9e-18
PLINK	0.72	4.0e-18	0.61	6.0e-18	0.42	4.0e-18	0.53	3.9e-18
GERMLINE-1	0.999	3.9e-18	0.997	4.0e-18	0.99	3.9e-18	0.99	3.9e-18
GERMLINE-2	0.999	3.9e-18	0.997	4.0e-18	0.98	3.9e-18	0.99	3.9e-18
DASH	0.999	3.9e-18	0.997	4.0e-18	0.99	3.9e-18	0.99	3.9e-18
Method	artAMis		artBMis		artCMis		artDMis	
	mean	p	mean	p	mean	p	mean	p
HapFABIA	0.06	——	0.04	——	0.11	——	0.14	——
fastIBD-1	0.98	3.9e-18	0.99	3.5e-18	0.95	3.9e-18	0.95	3.9e-18
fastIBD-2	0.88	3.9e-18	0.95	1.9e-18	0.85	3.9e-18	0.91	3.9e-18
PLINK	0.72	4.2e-18	0.61	3.9e-18	0.41	3.9e-18	0.53	3.9e-18
GERMLINE-1	0.999	3.9e-18	0.997	3.9e-18	0.99	3.9e-18	0.99	3.9e-18
GERMLINE-2	0.999	3.9e-18	0.996	3.9e-18	0.99	3.9e-18	0.99	3.9e-18
DASH	0.999	3.9e-18	0.997	3.9e-18	0.99	3.9e-18	0.99	3.9e-18
Method	artA		artB		artC		artD	
	mean	p	mean	p	mean	p	mean	p
HapFABIA	0.1944	——	0.1654	——	0.4513	——	0.5282	——
fastIBD-1	0.9997	3.9e-18	0.9995	3.9e-18	0.9977	3.9e-18	0.9957	3.9e-18
fastIBD-2	0.9972	3.9e-18	0.9953	3.9e-18	0.9779	3.9e-18	0.9718	3.9e-18
PLINK	0.9959	3.9e-18	0.9919	3.9e-18	0.9609	3.9e-18	0.9295	3.9e-18
GERMLINE-1	0.9999	3.9e-18	0.9998	3.9e-18	0.9991	3.9e-18	0.9989	3.9e-18
GERMLINE-2	0.9999	3.9e-18	0.9998	3.9e-18	0.9991	3.9e-18	0.9990	3.9e-18
DASH	0.9999	3.9e-18	0.9998	3.9e-18	0.9991	3.9e-18	0.9990	3.9e-18

Columns labeled "mean" show the mean false discovery rate (FDR) over 100 experiments. Columns labeled "p" provide the p-values of a Wilcoxon rank sum test over the 100 experiments with the null hypothesis that HapFABIA and another method yield the same value for the FDR. HapFABIA has significantly lower FDRs. Striking are the extremely high FDRs of all methods except HapFABIA for 1,000 individuals.

Table S12: Comparison of IBD detection methods for short segments on simulated data in terms of the F1 score.

Method	artA100		artB100		artC100		artD100	
	mean	p	mean	p	mean	p	mean	p
HapFABIA	0.87	——	0.65	——	0.82	——	0.67	——
fastIBD-1	0.04	3.9E-18	0.02	5.8E-16	0.09	3.9E-18	0.08	3.9E-18
fastIBD-2	0.20	3.9E-18	0.04	1.0E-15	0.11	3.9E-18	0.07	3.9E-18
PLINK	0.43	4.0E-18	0.55	7.9E-03	0.73	3.2E-14	0.63	1.8E-06
GERMLINE-1	0.00	3.9E-18	0.01	5.8E-16	0.03	3.9E-18	0.02	3.9E-18
GERMLINE-2	0.00	3.9E-18	0.01	5.8E-16	0.03	3.9E-18	0.02	3.9E-18
DASH	0.00	1.1E-17	0.01	5.8E-16	0.03	3.9E-18	0.02	3.9E-18
Method	artAMis		artBMis		artCMis		artDMis	
	mean	p	mean	p	mean	p	mean	p
HapFABIA	0.87	——	0.60	——	0.82	——	0.67	——
fastIBD-1	0.05	3.9E-18	0.03	1.5E-15	0.09	3.9E-18	0.09	3.9E-18
fastIBD-2	0.20	3.9E-18	0.07	3.5E-15	0.11	3.96-18	0.07	3.9E-18
PLINK	0.43	4.4E-18	0.55	9.1E-02	0.73	6.4E-13	0.63	2.8E-08
GERMLINE-1	0.00	3.9E-18	0.01	3.1E-15	0.03	3.9E-18	0.02	3.9E-18
GERMLINE-2	0.00	3.9E-18	0.01	3.1E-15	0.03	3.9E-18	0.02	3.9E-18
DASH	0.00	3.9E-18	0.01	3.1E-15	0.03	3.9E-18	0.02	3.9E-18
Method	artA		artB		artC		artD	
	mean	p	mean	p	mean	p	mean	p
HapFABIA	0.7504	——	0.4457	——	0.6329	——	0.492	——
fastIBD-1	0.0005	6.2E-18	0.0009	1.5E-13	0.0046	3.9E-18	0.009	3.9E-18
fastIBD-2	0.0056	6.2E-18	0.0092	9.9E-14	0.0432	3.9E-18	0.055	3.9E-18
PLINK	0.0081	6.2E-18	0.0160	1.5E-13	0.0752	3.9E-18	0.132	3.9E-18
GERMLINE-1	0.0001	6.2E-18	0.0004	1.5E-13	0.0018	3.9E-18	0.002	3.9E-18
GERMLINE-2	0.0000	6.2E-18	0.0004	1.5E-13	0.0018	3.9E-18	0.002	3.9E-18
DASH	0.0001	6.2E-18	0.0004	1.5E-13	0.0017	3.9E-18	0.002	3.9E-18

Columns labeled “mean” show the mean F1 score over 100 experiments. Columns labeled “p” provide the p-values of a Wilcoxon rank sum test over the 100 experiments with the null hypothesis that HapFABIA and another method yield the same value for the F1 score. The F1 score is the harmonic mean of precision (1 - FDR) and power and has an optimal value of 1 for perfect IBD detection. HapFABIA performs significantly better than all other methods on all data sets except artBMis for which the performance of PLINK is not significantly worse.

Table S13: Comparison of IBD detection methods for short segments on real data with implanted IBD segments in terms of power.

Method	impA		impB		impC		impD		impE	
	mean	p	mean	p	mean	p	mean	p	mean	p
HapFABIA	0.623	——	0.4408	——	0.5282	——	0.4227	——	0.6032	——
fastIBD-1	0.175	2.8e-11	0.0771	5.8e-08	0.9406	2.2e-14	0.0821	4.0e-18	0.1352	4.6e-18
fastIBD-2	0.042	2.4e-14	0.0234	3.8e-10	0.8386	1.3e-09	0.0332	4.0e-18	0.0329	4.0e-18
PLINK	0.998	1.3e-17	0.9883	1.8e-17	0.9995	3.5e-18	0.9858	4.0e-18	0.9972	4.0e-18
GERMLINE-1	0.667	7.1e-01	0.4081	4.3e-01	0.6402	3.5e-02	0.4137	3.6e-01	0.6576	1.0e-02
GERMLINE-2	0.265	5.9e-11	0.1429	7.3e-08	0.1948	7.7e-09	0.1547	4.3e-18	0.2640	1.1e-17
DASH	0.601	2.9e-03	0.3781	2.7e-02	0.6055	5.3e-03	0.3622	4.0e-02	0.6070	3.9e-01

Columns labeled "mean" show the mean power over 100 experiments. Columns labeled "p" provide the p-values of a Wilcoxon rank sum test over the 100 experiments with the null hypothesis that HapFABIA and another method yield the same value for the power. PLINK has an extremely high power, followed by GERMLINE-1 and HapFABIA.

Table S14: Comparison of IBD detection methods for short segments on real data with implanted IBD segments in terms of false discovery rate (FDR).

Method	impA		impB		impC		impD		impE	
	mean	p	mean	p	mean	p	mean	p	mean	p
HapFABIA	0.8376	——	0.94320	——	0.91237	——	0.4469	——	0.5826	——
fastIBD-1	0.9994	2.9e-14	0.99986	2.5e-08	0.99810	6.3e-10	0.9971	4.0e-18	0.9978	4.0e-18
fastIBD-2	0.9997	1.2e-14	0.99992	3.8e-10	0.99677	1.8e-10	0.9977	4.0e-18	0.9990	4.0e-18
PLINK	0.9997	7.8e-14	0.99983	7.9e-06	0.99981	8.7e-10	0.9967	4.0e-18	0.9984	4.0e-18
GERMLINE-1	0.9996	7.8e-14	0.99988	5.8e-06	0.99978	6.3e-10	0.9976	4.0e-18	0.9982	4.0e-18
GERMLINE-2	0.9997	5.1e-14	0.99991	3.5e-08	0.99985	1.4e-11	0.9980	4.0e-18	0.9984	4.0e-18
DASH	0.9998	1.2e-14	0.99992	4.7e-10	0.99990	1.6e-12	0.9983	4.0e-18	0.9989	4.0e-18

Columns labeled "mean" show the mean false discovery rate (FDR) over 100 experiments. Columns labeled "p" provide the p-values of a Wilcoxon rank sum test over the 100 experiments with the null hypothesis that HapFABIA and another method yield the same value for the FDR. HapFABIA has significantly lower FDRs than other methods. The FDRs of other methods than HapFABIA are too large for feasible IBD detection. The precision (1 - FDR) of HapFABIA is more than 150 times larger than the precision of other methods.

Table S15: Comparison of IBD detection methods for short segments on real data with implanted IBD segments in terms of the F1 score.

Method	impA		impB		impC		impD		impE	
	mean	p	mean	p	mean	p	mean	p	mean	p
HapFABIA	0.2326	——	0.0969	——	0.1445	——	0.469	——	0.4785	——
fastIBD-1	0.0012	5.4e-14	0.0003	2.5e-08	0.0038	6.3e-10	0.006	4.0e-18	0.0044	4.0e-18
fastIBD-2	0.0006	1.8e-14	0.0002	3.8e-10	0.0064	1.8e-10	0.004	4.0e-18	0.0020	4.0e-18
PLINK	0.0006	1.6e-13	0.0003	7.9e-06	0.0004	8.7e-10	0.007	4.0e-18	0.0032	4.0e-18
GERMLINE-1	0.0008	1.6e-13	0.0002	5.8e-06	0.0004	6.3e-10	0.005	4.0e-18	0.0037	4.0e-18
GERMLINE-2	0.0007	8.0e-14	0.0002	3.5e-08	0.0003	1.4e-11	0.004	4.0e-18	0.0032	4.0e-18
DASH	0.0004	1.8e-14	0.0002	4.7e-10	0.0002	1.6e-12	0.003	4.0e-18	0.0022	4.0e-18

Columns labeled "mean" show the mean F1 score over 100 experiments. Columns labeled "p" provide the p-values of a Wilcoxon rank sum test over the 100 experiments with the null hypothesis that HapFABIA and another method yield the same value for the F1 score. HapFABIA performs significantly better than all other methods on all data sets.

Table S16: Comparison of IBD detection methods for short segments on forward simulation data with implanted IBD segments in terms of power.

Method	simA		simB		simC		simD		simE	
	mean	p	mean	p	mean	p	mean	p	mean	p
HapFABIA	0.687	——	0.58	——	0.69	——	0.56	——	0.71	——
fastIBD-1	0.142	5e-16	0.14	4e-12	0.45	2e-07	0.15	4e-18	0.15	4e-18
fastIBD-2	0.050	4e-16	0.05	4e-13	0.26	5e-14	0.06	4e-18	0.06	4e-18
PLINK	0.396	4e-08	0.13	3e-12	0.30	9e-11	0.12	4e-18	0.38	1e-17
GERMLINE-1	0.937	2e-11	0.78	2e-04	0.94	2e-10	0.77	6e-18	0.94	8e-18
GERMLINE-2	0.003	2e-15	0.01	2e-12	0.03	3e-15	0.01	4e-18	0.02	4e-18
DASH	0.911	9e-10	0.76	2e-04	0.90	4e-07	0.75	6e-18	0.91	2e-16

Columns labeled "mean" show the mean power over 100 experiments. Columns labeled "p" provide the p-values of a Wilcoxon rank sum test over the 100 experiments with the null hypothesis that HapFABIA and another method yield the same value for the power. GERMLINE-1, DASH, and HapFABIA have a high power.

Table S17: Comparison of IBD detection methods for short segments on forward simulation data with implanted IBD segments in terms of false discovery rate.

Method	simA		simB		simC		simD		simE	
	mean	p	mean	p	mean	p	mean	p	mean	p
HapFABIA	0.4174	——	0.1194	——	0.4696	——	0.0252	——	0.2170	——
fastIBD-1	0.9999	8e-17	0.9999	7e-18	0.9998	2e-17	0.9987	4e-18	0.9994	4e-18
fastIBD-2	0.9999	2e-16	0.9999	3e-18	0.9996	6e-17	0.9986	4e-18	0.9993	4e-18
PLINK	0.9994	7e-17	0.9992	6e-18	0.9997	2e-17	0.9861	4e-18	0.9968	4e-18
GERMLINE-1	0.9998	6e-17	0.9999	7e-18	0.9999	2e-17	0.9981	4e-18	0.9991	4e-18
GERMLINE-2	0.9900	2e-15	0.9799	9e-20	0.9898	5e-17	0.9887	2e-18	0.9791	9e-18
DASH	0.9998	6e-17	0.9999	9e-19	0.9999	2e-17	0.9991	4e-18	0.9991	4e-18

Columns labeled “mean” show the mean false discovery rate (FDR) over 100 experiments. Columns labeled “p” provide the p-values of a Wilcoxon rank sum test over the 100 experiments with the null hypothesis that HapFABIA and another method yield the same value for the FDR. HapFABIA has significantly lower FDRs than other methods. The FDRs of other methods than HapFABIA are too large for feasible IBD detection.

Table S18: Comparison of IBD detection methods for short segments on forward simulation data with implanted IBD segments in terms of the F1 score.

Method	simA		simB		simC		simD		simE	
	mean	p	mean	p	mean	p	mean	p	mean	p
HapFABIA	0.5714	——	0.5619	——	0.4654	——	0.708	——	0.728	——
fastIBD-1	0.0003	2e-16	0.0001	4e-12	0.0005	4e-16	0.003	4e-18	0.001	4e-18
fastIBD-2	0.0002	4e-16	0.0001	4e-12	0.0007	1e-15	0.003	4e-18	0.001	4e-18
PLINK	0.0013	2e-15	0.0016	8e-13	0.0006	3e-16	0.025	4e-18	0.006	4e-18
GERMLINE-1	0.0004	2e-16	0.0002	3e-11	0.0002	4e-16	0.004	4e-18	0.002	4e-18
GERMLINE-2	0.0001	2e-15	0.0002	2e-12	0.0003	3e-15	0.002	4e-18	0.002	4e-18
DASH	0.0004	2e-16	0.0002	4e-13	0.0002	4e-16	0.003	4e-18	0.002	4e-18

Columns labeled “mean” show the mean F1 score over 100 experiments. Columns labeled “p” provide the p-values of a Wilcoxon rank sum test over the 100 experiments with the null hypothesis that HapFABIA and another method yield the same value for the F1 score. HapFABIA performs significantly better than all other methods on all data sets.

Table S19: Comparison of IBD detection methods for long segments on forward simulation data with implanted IBD segments in terms of power.

Method	simAlong		simBlong		simClong		simDlong		simElong		simFlong	
	m	p	m	p	m	p	m	p	m	p	m	p
HapFABIA	0.96	—	0.94	—	0.98	—	0.97	—	0.93	—	0.93	—
fastIBD-1	0.95	6e-03	0.76	1e-14	0.97	6e-06	0.81	1e-16	0.94	6e-01	0.77	3e-10
fastIBD-2	0.91	3e-11	0.61	2e-15	0.94	3e-14	0.70	8e-17	0.89	7e-02	0.69	3e-12
PLINK	0.99	2e-15	0.96	3e-16	0.99	9e-17	0.98	2e-16	0.99	6e-18	0.99	4e-18
GERMLINE-1	0.91	2e-10	0.87	5e-11	0.96	2e-09	0.93	8e-13	0.86	1e-06	0.80	3e-10
GERMLINE-2	0.91	2e-10	0.88	3e-10	0.96	2e-09	0.93	8e-13	0.91	4e-04	0.88	8e-10
DASH	0.89	2e-12	—	—	0.96	3e-12	—	—	0.00	4e-18	—	—

Columns labeled "m" show the mean power over 100 experiments. Columns labeled "p" provide the p-values of a Wilcoxon rank sum test over the 100 experiments with the null hypothesis that HapFABIA and another method yield the same value for the power. PLINK has an extremely high power, followed by HapFABIA. In summary, all methods have high power.

Table S20: Comparison of IBD detection methods for long segments on forward simulation data with implanted IBD segments in terms of false discovery rate.

Method	simAlong		simBlong		simClong		simDlong		simElong		simFlong	
	m	p	m	p	m	p	m	p	m	p	m	p
HapFABIA	0.10	—	0.01	—	0.07	—	0.02	—	0.01	—	0.01	—
fastIBD-1	0.07	7e-02	0.11	2e-11	0.04	5e-02	0.04	7e-10	0.30	5e-18	0.54	2e-17
fastIBD-2	0.04	3e-01	0.03	1e-00	0.02	8e-02	0.00	1e-00	0.10	5e-14	0.07	4e-08
PLINK	0.99	4e-18	0.99	4e-18	0.98	4e-18	0.99	4e-18	0.99	4e-18	0.99	4e-18
GERMLINE-1	0.01	6e-02	0.00	1e-00	0.00	3e-01	0.00	1e-00	0.75	4e-18	0.91	4e-18
GERMLINE-2	0.01	6e-02	0.01	1e-00	0.00	4e-01	0.00	1e-00	0.96	4e-18	0.99	4e-18
DASH	0.00	3e-02	—	—	0.00	2e-02	—	—	0.00	1e-00	—	—

Columns labeled "m" show the mean false discovery rate (FDR) over 100 experiments. Columns labeled "p" provide the p-values of a Wilcoxon rank sum test over the 100 experiments with the null hypothesis that HapFABIA and another method yield the same value for the FDR. The FDR of PLINK is too large to be feasible for IBD detection. All other methods have a very low FDR for long IBD segments (simAlong - simDlong). For medium sized IBD segments (simElong, simFlong) GERMLINE has a large FDR. HapFABIA has significantly lower FDR for the medium sized IBD segments (simElong and simFlong).

Table S21: Comparison of IBD detection methods for long segments on forward simulation data with implanted IBD segments in terms of the F1 score.

Method	simAlong		simBlong		simClong		simDlong		simElong		simFlong	
	m	p	m	p	m	p	m	p	m	p	m	p
HapFABIA	0.91	—	0.95	—	0.94	—	0.97	—	0.96	—	0.95	—
fastIBD-1	0.94	2e-01	0.80	5e-14	0.96	8e-02	0.88	1e-13	0.80	2e-17	0.55	4e-17
fastIBD-2	0.93	4e-02	0.73	5e-15	0.96	5e-02	0.82	3e-14	0.89	9e-11	0.77	2e-13
PLINK	0.03	6e-18	0.01	2e-17	0.05	4e-18	0.02	6e-18	0.00	4e-18	0.00	5e-18
GERMLINE-1	0.94	6e-01	0.90	4e-10	0.98	9e-01	0.96	2e-11	0.39	4e-18	0.16	5e-18
GERMLINE-2	0.94	6e-01	0.92	2e-09	0.98	9e-01	0.95	2e-11	0.08	4e-18	0.03	5e-18
DASH	0.93	3e-01	—	—	0.98	9e-01	—	—	0.00	4e-18	—	—

Columns labeled "m" show the mean F1 score over 100 experiments. Columns labeled "p" provide the p-values of a Wilcoxon rank sum test over the 100 experiments with the null hypothesis that HapFABIA and another method yield the same value for the F1 score. However, for simBlong, simDlong, simElong, and simFlong HapFABIA has a significantly higher F1 score than other methods.

S15 Examples of IBD Segments that Match Archaic Genomes

Figure S26 shows a typical example of an IBD segment that matches the Denisova genome and is shared exclusively among Asians. Figure S27 shows an IBD segment that matches the Denisova genome and is shared by Africans and admixed Americans, and Fig. S28 shows an IBD segment that matches the Denisova genome and is shared across all populations. Furthermore, Fig. S28 reveals three phasing errors in the individuals where a yellow line starts from the left hand side: HG01271, NA12890, and NA19137. Figure S29 shows an example of an IBD segment that matches the Neandertal genome and is shared by Europeans and admixed American. Figure S30 shows an example of an IBD segment that matches the Neandertal genome and is shared by Europeans and Asians. Figure S31 shows an IBD segment that matches the Neandertal genome and is found exclusively in Africans. Another phasing error is visible in this figure in individual NA19317. Interestingly, IBD segments containing parts of the Denisova genome or the Neandertal genome are often shared by Africans.

Figure S32 shows an example of an IBD segment that matches the Denisova genome and is shared by Japanese. Further it can be seen that only a part of the IBD segment matches the Denisova genome. Figure S33 shows a second example of an IBD segment that matches the Denisova genome and is shared by Japanese. Again only a part of the IBD segment matches the Denisova genome.

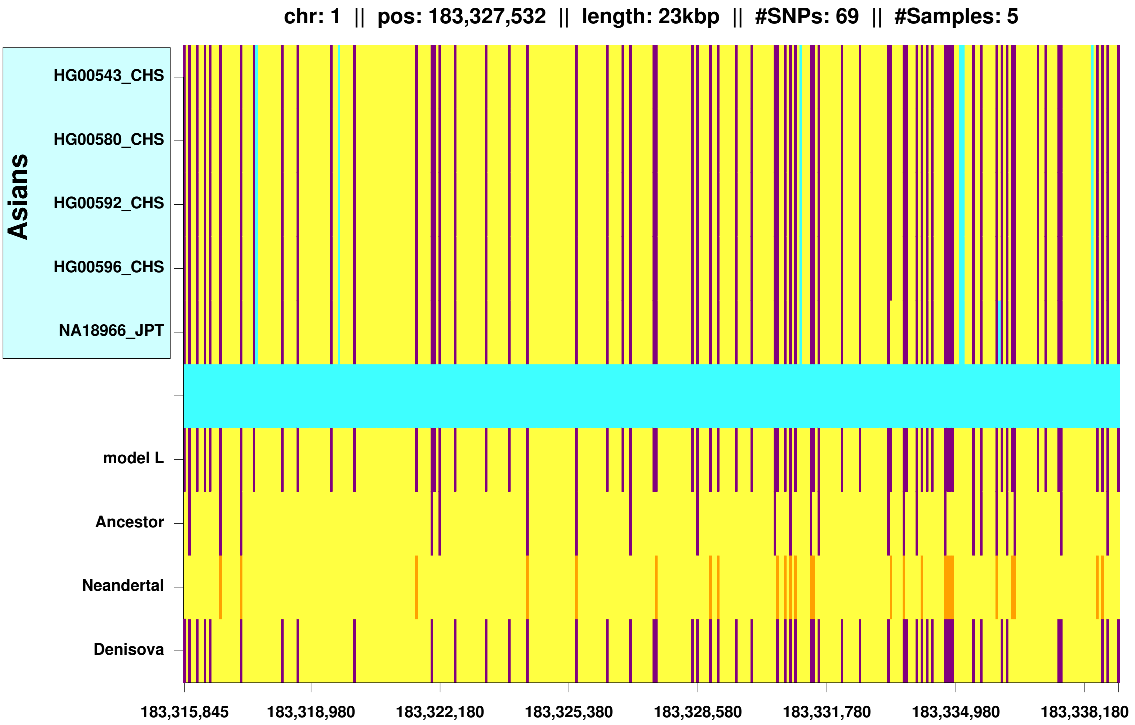

Figure S26: Example of an IBD segment matching the Denisova genome shared exclusively among Asians. The data analyzed by HapFABIA were phased genotypes from chromosome 1 of the 1000 Genomes Project. The rows give all chromosomes that contain the IBD segment and columns consecutive SNVs. If both chromosomes of an individual contain the IBD segment then two adjacent identical row labels are present. Major alleles are shown in yellow, minor alleles of tagSNVs in violet, and minor alleles of other SNVs in cyan. The row labeled “model L” indicates tagSNVs identified by HapFABIA in violet. The rows “Ancestor”, “Neandertal”, and “Denisova” show bases of the respective genomes in violet if they match the minor allele of the tagSNVs (in yellow otherwise). Neandertal tagSNV bases that are not called are shown in orange.

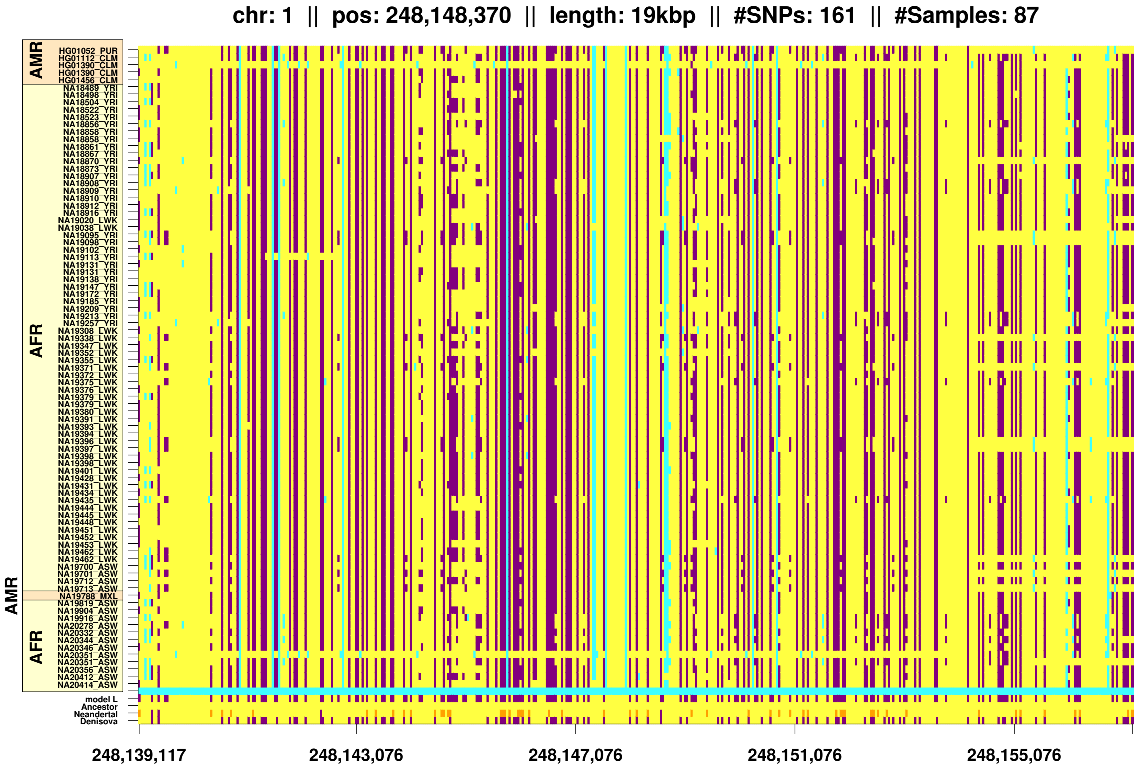

Figure S27: Example of an IBD segment matching the Denisova genome shared by Africans and admixed Americans. See Fig. S26 for a description.

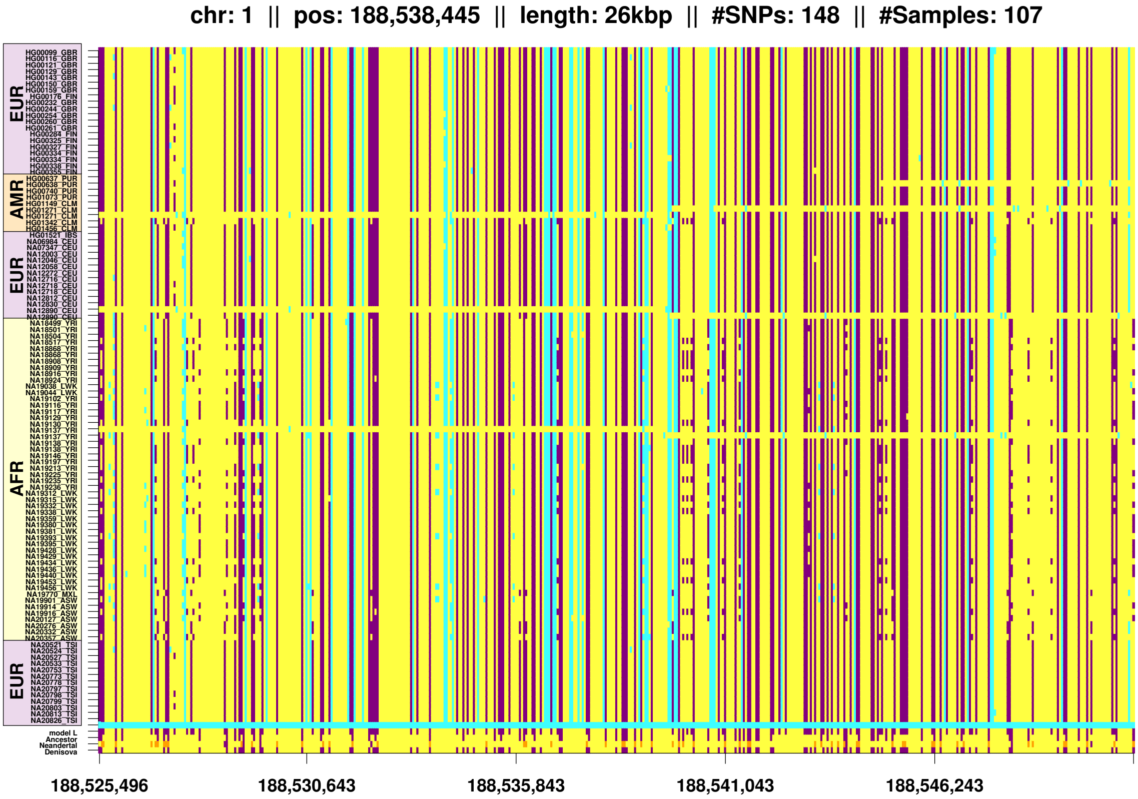

Figure S28: Example of an IBD segment matching the Denisova genome shared by Africans, Europeans, and admixed Americans. Phasing errors can be seen at individuals that share only some part of the IBD segments (partly empty rows): HG01271, NA12890, and NA19137. See Fig. S26 for a description.

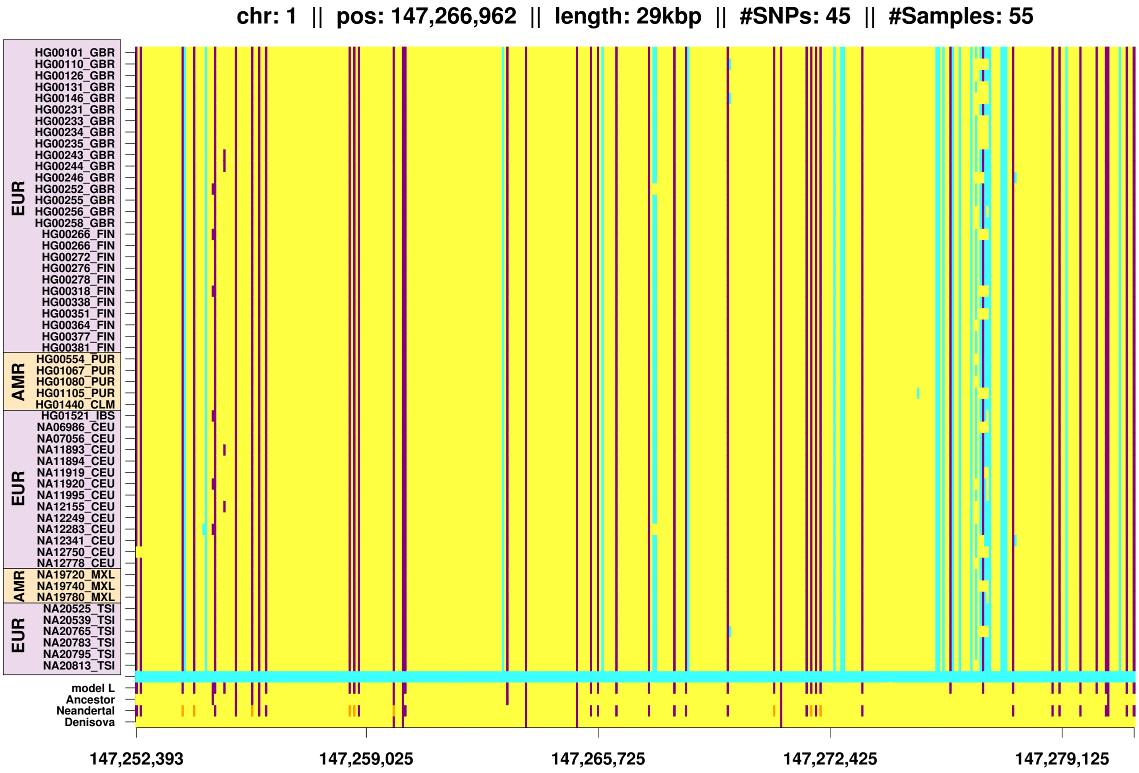

Figure S29: Example of an IBD segment matching the Neandertal genome shared by Europeans and admixture Americans. See Fig. S26 for a description.

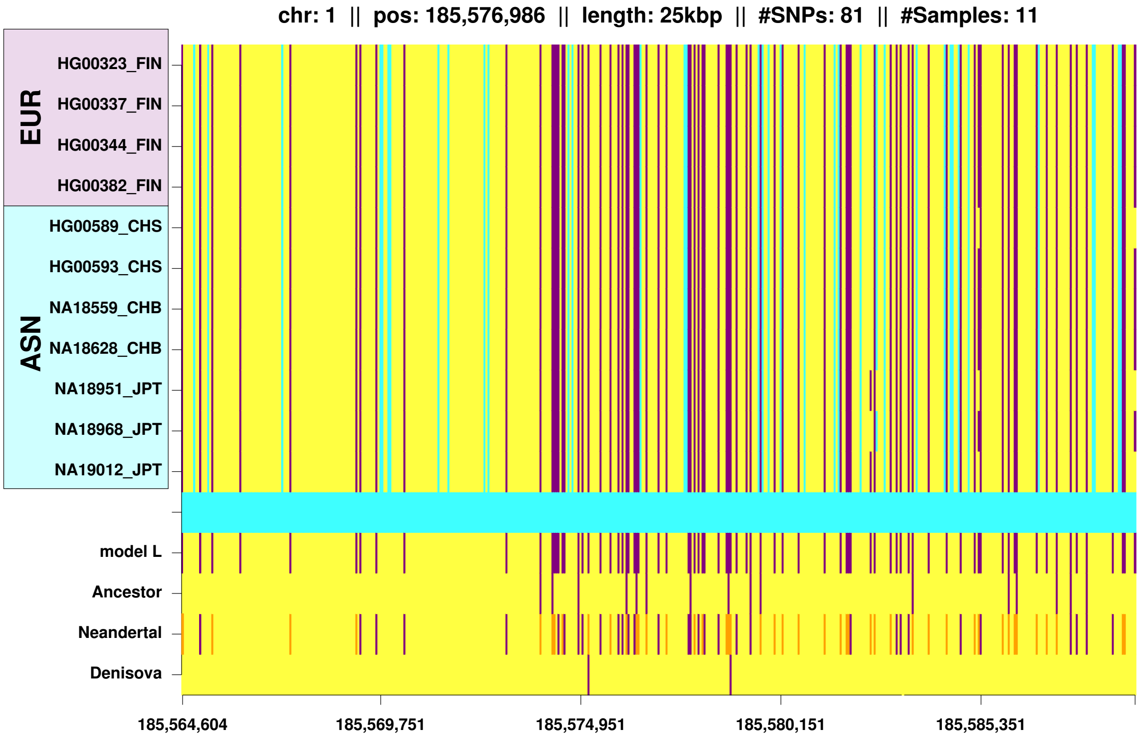

Figure S30: Example of an IBD segment matching the Neandertal genome shared by Europeans and Asians. See Fig. S26 for a description.

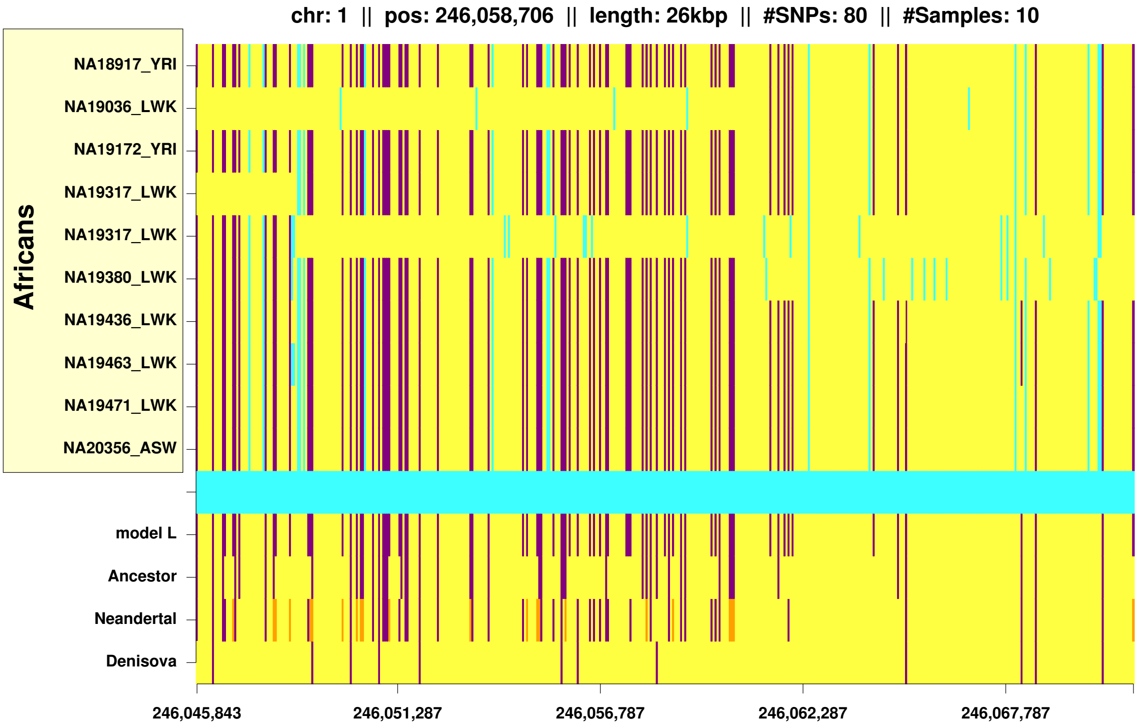

Figure S31: Example of an IBD segment matching the Neandertal genome shared exclusively among Africans. A phasing error is visible at individual NA19317. See Fig. S26 for a description.

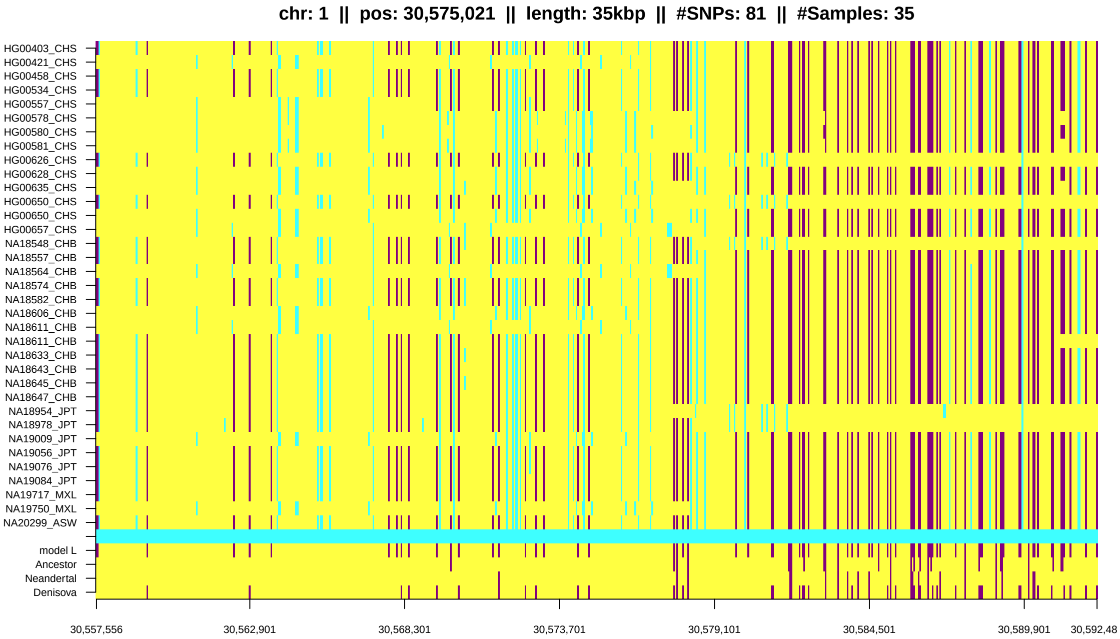

Figure S32: Example 1 of an IBD segment that matches the Denisovan genome and is shared by Japanese. Only a part of the IBD segment matches the Denisovan genome. See Fig. S26 for a description.

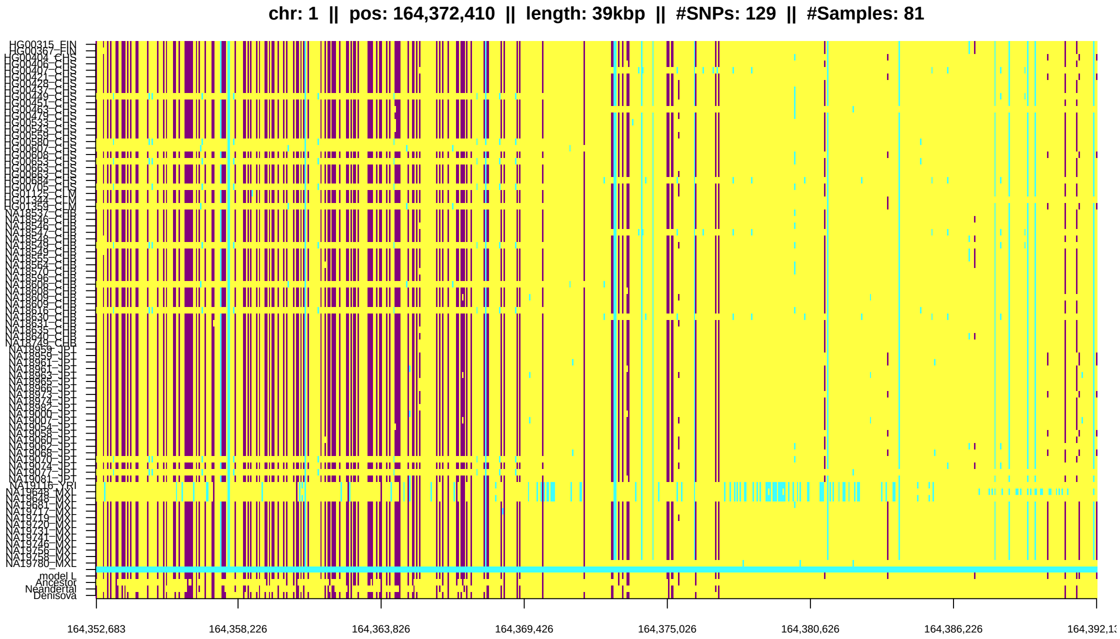

Figure S33: Example 2 of an IBD segment that matches the Denisovan genome and is shared by Japanese. Only a part of the IBD segment matches the Denisovan genome. See Fig. S26 for a description.

References

1. Box, G. E. P., Hunter, W. G., and Hunter, J. S. (1978) Statistics for Experimenters: An Introduction to Design, Data Analysis, and Model Building, Wily, .

2. Hyvärinen, A. and Oja, E. (1999) A fast fixed-point algorithm for independent component analysis. *Neural Comput.,* **9**(7), 1483–1492.

3. Girolami, M. (2001) A Variational Method for Learning Sparse and Overcomplete Representations. *Neural Comput.,* **13**(11), 2517–2532.

4. Palmer, J., Wipf, D., Kreutz-Delgado, K., and Rao, B. (2006) Variational EM algorithms for non-Gaussian latent variable models. *Advances in Neural Information Processing Systems,* **18**, 1059–1066.

5. Hochreiter, S., Bodenhofer, U., Heusel, M., Mayr, A., Mitterecker, A., Kasim, A., VanSanden, S., Lin, D., Talloen, W., Bijnens, L., et al. (2010) FABIA: factor analysis for bicluster acquisition. *Bioinformatics,* **26**(12), 1520–1527.

6. Clevert, D.-A., Mitterecker, A., Mayr, A., Klambauer, G., Tuefferd, M., DeBondt, A., Talloen, W., Göhlmann, H. W. H., and Hochreiter, S. (2011) cn.FARMS: a latent variable model to detect copy number variations in microarray data with a low false discovery rate. *Nucleic Acids Res.,* **39**(12), e79.

7. Klambauer, G., Schwarzbauer, K., Mayr, A., Clevert, D.-A., Mitterecker, A., Bodenhofer, U., and Hochreiter, S. (2012) cn.MOPS: mixture of Poissons for discovering copy number variations in next generation sequencing data with a low false discovery rate. *Nucleic Acids Res.,* **40**(9), e69.

8. Slatkin, M. (2008) Linkage disequilibrium — understanding the evolutionary past and mapping the medical future. *Nat. Rev. Genet.,* **9**, 477–485.

9. Wall, J. D. and Pritchard, J. K. (2003) Haplotype blocks and linkage disequilibrium in the human genome. *Nat. Rev. Genet.,* **4**(8), 587–597.

10. Gabriel, S. B., Schaffner, S. F., Nguyen, H., Moore, J. M., Roy, J., Blumenstiel, B., Higgins, J., DeFelice, M., Lochner, A., Faggart, M., et al. (2002) The Structure of Haplotype Blocks in the Human Genome. *Science,* **296**(5576), 2225–2229.

11. Browning, B. L. and Browning, S. R. (2011) A fast, powerful method for detecting identity by descent. *Am. J. Hum. Genet.,* **88**(2), 173–182.

12. Su, S.-Y., Kasberger, J., Baranzini, S., Byerley, W., Liao, W., Oksenberg, J., Sherr, E., and Jorgenson, E. (2012) Detection of identity by descent using next-generation whole genome sequencing data. *BMC Bioinformatics,* **13**, 121.

13. Ewing, G. and Hermisson, J. (2010) MSMS: a coalescent simulation program including recombination, demographic structure and selection at a single locus. *Bioinformatics,* **26**(16), 2064–2065.

14. Liang, L., Zöllner, S., and Abecasis, G. R. (2007) GENOME: a rapid coalescent-based whole genome simulator. *Bioinformatics,* **23**(12), 1565–1567.

15. Schaffner, S. F., Foo, C., Gabriel, S., Reich, D., Daly, M. J., and Altshuler, D. (2005) Calibrating a coalescent simulation of human genome sequence variation. *Genome Res.,* **15**, 1576–1783.

16. Hoban, S., Bertorelle, G., and Gaggiotti, O. E. (2012) Computer simulations: tools for population and evolutionary genetics. *Nat. Rev. Genet.,* **13**, 110–122.

17. Peng, B., Amos, C. I., and Kimmel, M. (2007) Forward-Time Simulations of Human Populations with Complex Diseases. *PLoS Genet.,* **3**(3), e47.

18. Hernandez, R. D. (2008) A flexible forward simulator for populations subject to selection and demography. *Bioinformatics,* **24**(23), 2786–2787.

19. Maher, M. C., Uricchio, L. H., Torgerson, D. G., and Hernandez, R. D. (2013) Population genetics of rare variants and complex diseases. In *arXiv* number 1302.2710 in [q-bio.PE] WWW.

20. Meuwissen, T. H. E. and Goddard, M. E. (2001) Prediction of identity by descent probabilities from marker-haplotypes. *Genet. Sel. Evol.,* **33**(6), 605–634.

21. Browning, S. R. (2008) Estimation of pairwise identity by descent from dense genetic marker data in a population sample of haplotypes. *Genetics,* **178**(4), 2123–2132.

22. Strachan, T. and Read, A. P. (2004) Human Molecular Genetics, Garland Science/Taylor & Francis Group, 3rd edition.

23. Zaitlen, N., Kang, H. M., and Eskin, E. (2009) Linkage Effects and Analysis of Finite Sample Errors in the HapMap. *Hum. Hered.,* **68**, 73–86.

24. Barrett, J. C. and Cardon, L. R. (2006) Evaluating coverage of genome-wide association studies. *Nat. Genet.,* **38**(6), 659–662.

25. The 1000 Genomes Project Consortium (2012) An integrated map of genetic variation from 1,092 human genomes. *Nature,* **491**(7422), 56–65.

26. Gusev, A., Palamara, P. F., Aponte, G., Zhuang, Z., Darvasi, A., Gregersen, P., and Pe'er, I. (2012) The architecture of long-range haplotypes shared within and across populations. *Mol. Biol. Evol.,* **29**(2), 473–486.

27. Thomas, A., Skolnick, M. H., and Lewis, C. M. (1994) Genomic mismatch scanning in pedigrees. *Math. Med. Biol.,* **11**(1), 1–16.

28. Thomas, A., Camp, N. J., Farnham, J. M., Allen-Brady, K., and Cannon-Albright, L. A. (2008) Shared genomic segment analysis. Mapping disease predisposition genes in extended pedigrees using SNP genotype assays. *Ann. Hum. Genet.,* **72**(Pt 2), 279–287.

29. Hacia, J. G., Fan, J.-B., Ryder, O., Jin, L., Edgemon, K., Ghandour, G., Mayer, R. A., Sun, B., Hsie, L., Robbins, C. M., et al. (1999) Determination of ancestral alleles for human single-nucleotide polymorphisms using high-density oligonucleotide arrays. *Nat. Genet.,* **22**(2), 164–167.

30. The Chimpanzee Sequencing and Analysis Consortium (2005) Initial sequence of the chimpanzee genome and comparison with the human genome. *Nature,* **437**, 69–87.

31. Leffler, E. M., Gao, Z., Pfeifer, S., Ségurel, L., Auton, A., Venn, O., Bowden, R., Bontrop, R., Wall, J. D., Sella, G., et al. (2013) Multiple instances of ancient balancing selection shared between humans and chimpanzees. *Science,* **339**(6127), 1578–1582.

32. Fu, W., O'Connor, T. D., Jun, G., Kang, H. M., Abecasis, G., Leal, S. M., Gabriel, S., Altshuler, D., Shendure, J., Nickerson, D. A., et al. (2013) Analysis of 6,515 exomes reveals the recent origin of most human protein-coding variants. *Nature,* **493**(7431), 216–220.

33. Gravel, S., Henn, B. M., Gutenkunst, R. N., Indap, A. R., Marth, G. T., Clark, A. G., Yu, F., Gibbs, R. A., The 1000 Genomes Project, and Bustamante, C. D. (2011) Demographic history and rare allele sharing among human populations. *P. Natl. Acad. Sci. USA,* **108**(29), 11983–11988.

34. Palamara, P. F., Lencz, T., Darvasi, A., and Pe'er, I. (2012) Length Distributions of Identity by Descent Reveal Fine-Scale Demographic History. *Am. J. Hum. Genet.,* **91**(5), 809–822.

35. Ulgen, A. and Li, W. (2005) Comparing single-nucleotide-polymorphism marker-based and microsatellite marker-based linkage analyses. *BMC Genet.,* **6**, S13.

36. Yu, A., Zhao, C., Fan, Y., Jang, W., Mungall, A. J., Deloukas, P., Olsen, A., Doggett, N. A., Ghebranious, N., Broman, K. W., et al. (2001) Comparison of human genetic and sequence-based physical maps. *Nature,* **409**, 951–953.

37. Green, R. E., Krause, J., Briggs, A. W., Maricic, T., Stenzel, U., Kircher, M., Patterson, N., Li, H., Zhai, W., Fritz, M. H., et al. (2010) A draft sequence of the Neandertal genome. *Science,* **328**(5979), 710–722.

38. Hammer, M. F., Woerner, A. E., Mendez, F. L., Watkins, J. C., and Wall, J. D. (2011) Genetic evidence for archaic admixture in Africa. *P. Natl. Acad. Sci. USA,* **10**(37), 15123–15128.

39. Pennisi, E. (2013) More Genomes From Denisova Cave Show Mixing of Early Human Groups. *Science,* **340**(6134), 799.
